# Effect of rPET Content and Preform Heating/Cooling Conditions in the Stretch Blow Molding Process on Microcavitation and Solid-State Post-Condensation of vPET-rPET Blend: Part I—Research Methodology and Results

**DOI:** 10.3390/ma17215233

**Published:** 2024-10-27

**Authors:** Paweł Wawrzyniak, Waldemar Karaszewski, Artur Różański

**Affiliations:** 1 Faculty of Automotive and Construction Machinery Engineering, Warsaw University of Technology, 84 Ludwika Narbutta Street, 02-524 Warsaw, Poland; 2 Faculty of Mechanical Engineering and Ship Technology, Gdańsk University of Technology, 11/12 Gabriela Narutowicza Street, 80-233 Gdańsk, Poland; waldemar.karaszewski@pg.edu.pl; 3 Centre of Molecular and Macromolecular Studies, Polish Academy of Sciences, 112 Sienkiewicza Street, 90-363 Łódz, Poland; artur.rozanski@cbmm.lodz.pl

**Keywords:** PET recycling, SBM process, microcavitation, solid state post-condensation, power of ANOVA test in DOE, positron annihilation lifetime spectroscopy (PALS)

## Abstract

Polyethylene terephthalate (PET) is widely used in bottle production due to its cost-effectiveness and low environmental impact. The first part of this article describes the research and statistical analysis methodology of the influence of the virgin PET (vPET) and recycled PET (rPET) content in the vPET-rPET blend, as well as the preform heating/cooling conditions in the stretch blow molding (SBM) process on the microscopic bottle properties. Microscopic properties such as crystallinity, density, viscosity, relaxation degree of the amorphous phase, and microcavitation in PET were examined. This study reveals that microcavity and solid-state post-condensation effects occur during PET deformation in the SBM process. The increase in free volume, indicating microcavitation, was confirmed by measuring positron annihilation lifetime spectroscopy (PALS). PALS and density of the amorphous phase studies prove a reduction in the dimensions of the free volumes, with a simultaneous significant increase in their number and ellipsoidization. It can be associated with crystallite rotation in a temperature-dependent non-crystalline matrix. The occurrence of solid-state post-condensation effects was confirmed by measuring the intrinsic viscosity. The conclusions resulting from the analysis of the microstructure affecting the mechanical strength of the material were validated by pressure resistance tests of the bottles.

## 1. Introduction

In recent years, reusing polymer materials has become increasingly important due to environmental protection. This is crucial in the case of a circular economy, i.e., one in which materials should remain in circulation as long as possible to significantly minimize waste generation. Poly(ethylene terephthalate) (PET) is extensively utilized in packaging for carbonated and non-carbonated drinks because of its cost-effectiveness and minimal carbon footprint [1,2,3]. Thus, the use of recycled PET (rPET) for the manufacture of such products seems to be a necessity. Nevertheless, the content of rPET causes significant changes in the internal structure of PET after the stretch blow molding (SBM) process (forming bottles/packages), and this structure is closely related to their physicochemical properties [3,4]. PET recyclate (rPET) in the form of granulate (to manufacture preforms (https://www.hanex.com.pl/en/preforms/ (accessed on 19 September 2024)) with rPET content used in this study) was obtained using the material recycling method to comply fully with Directive 94/62/EU (https://eur-lex.europa.eu/eli/dir/1994/62/oj (accessed on 19 September 2024)).

The problem of polymeric waste disposal has gained significant attention because of its environmental impact. In 2021, PET packaging constituted 12% of the world’s solid waste [3] (in 2020, just 23% of 7297.7 kilotons of consumed PET were recycled [5]). Tackling this issue demands a strategic approach to managing polymeric packaging waste (it is estimated that around 1 million bottles/min are produced worldwide [6]). PET bottles have several eco-friendly features, including strong barrier properties, and are made from a single material, which enhances their recyclability. The FDA (Food and Drug Administration) states that modern recycling advancements have rendered post-consumer recycled PET a safe and practical option for beverage packaging [3].

Other articles provide a comprehensive review of the stretch blow molding process using both cold and hot molds [7,8,9,10,11,12]. In the first two publications, the production stages of PET containers in the SBM and the phase transitions of the processed PET were discussed in detail [7,8]. In the next two papers, the impact of preform reheating and hot mold stretch blow molding parameters on PET container properties was examined [9,10]. It was noticed that polymer orientation and crystallization processes have the greatest impact on the mechanical and thermal properties of PET bottles obtained in the injection stretch blow molding (ISBM) process. Therefore, the final properties of PET bottles, including thermal stability and pressure resistance, are primarily influenced by factors such as the initial structure of the PET preform, its geometry and temperature distribution, the blow mold’s geometry and temperature distribution, and specific SBM process parameters, notably the pre-blow start delay relative to the stretching rod’s position. Previous papers [11,12] extensively discussed the effects of blow mold temperature in the SBM process and the hot filling method on selected properties of PET bottles. The blow kinetics study revealed a material flow gradient along the bottle wall’s thickness during blowing, which can be directly linked to the air temperature between the blow mold and the bottle wall. Analyzing PET’s density, crystallinity, and amorphous phase relaxation demonstrated that microcavitation, associated with the orientation of the polymer microstructure, is initiated during polymer deformation in SBM.

In general, optimizing the design of PET and rPET beverage bottles involves three main aspects: material, process, and physical structure. While material and process optimization research is extensive, physical-based structural optimization primarily focuses on enhancing mechanical properties. Ideally, a holistic approach that integrates all three optimization aspects is preferred, although it is essential to acknowledge the increasing complexity of engineering involved [13].

Due to the large scope of the research, the entire work was divided into two parts to increase its clarity. In addition, a literature review was developed in a separate article [14], which discussed in detail the current knowledge on the phenomena of cavitation and post-condensation occurring in the PET material. The main aim of the present work is to describe the research and statistical analysis methodology of the influence of rPET content and preform heating/cooling conditions in the SBM process on the microscopic preform and bottle material properties (crystallinity, density (ρ), intrinsic viscosity (IV), relaxation of the amorphous phase, and microcavitation (M.E.)). The microcavitation phenomenon based on the PALS analysis and causal relationship between solid-state post-condensation and microcavitation processes is a new approach for rPET containers made by the blowing process. The conclusions resulting from the analysis of the microstructure affecting the mechanical strength of the material were validated by pressure resistance tests of the containers. For this purpose, experiments employing response surface methodology were performed for the three analyzed factors (independent variables), i.e., rPET content, lamp heating power, and fan cooling power, for bottle material, and experiments employing the same methodology for the two analyzed factors (independent variables), i.e., rPET content, and SBM treated as one independent process, for microscopic properties of bottle material relative to microscopic properties of preform material. ANOVA (analysis of variance) and power of ANOVA in the design of experiments (DOE) were carried out. The sample size was based on the authors’ previous research [11,12]. Additionally, a valuable value of this part of the work is shown in Table S2, an original tabular presentation of the interpretation of two-factor cross-effects in multivariate statistical analyses, i.e., the influence of the value and sign of the two-way interaction AxB on the interpretation of the effect of Factor A depending on the level of Factor B and vice versa. The description and interpretation of the results of the statistical analysis are given in the second part of this paper [15].

This part and the second part of the work are also unique in terms of the use of positron annihilation lifetime spectroscopy (PALS) to measure microcavitation in rPET material. PALS is an effective analytical method for PET [16,17,18,19,20], known for its capacity to assess the size of free volume voids in amorphous materials and imperfections within the crystalline domains of PET [21]. For over four decades, PALS has been employed as an experimental technique to measure local free volume [22,23,24,25]. PALS provides insights into cavity size within a material by correlating with the time between positron irradiation and positron annihilation.

## 2. Research Methodology

### 2.1. Research Plan

**Figure 1:**
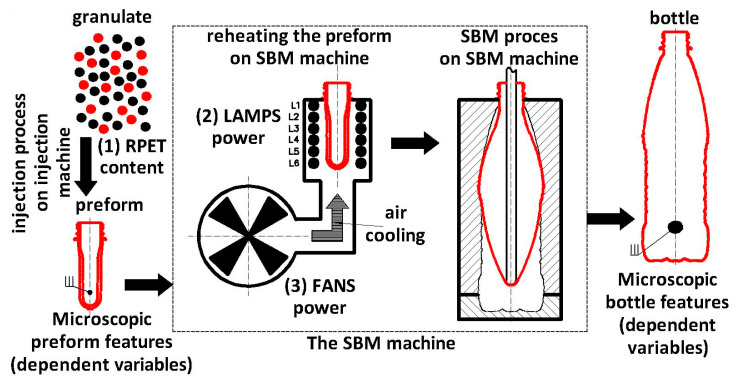
A schematic explanation of the SBM process with the selection of independent variables (rPET content, heating lamp power, cooling fan power, and SBM process treated as a whole) and dependent variables as microscopic features of the preform.

A schematic explanation of the SBM process with the selection of independent variables (rPET content, heating lamp power (power of LAMPS), cooling fan power (power of FANS), and SBM process treated as a whole) and dependent variables as microscopic features of the preform, and the bottle is shown in Figure 1.

**Figure A1:**
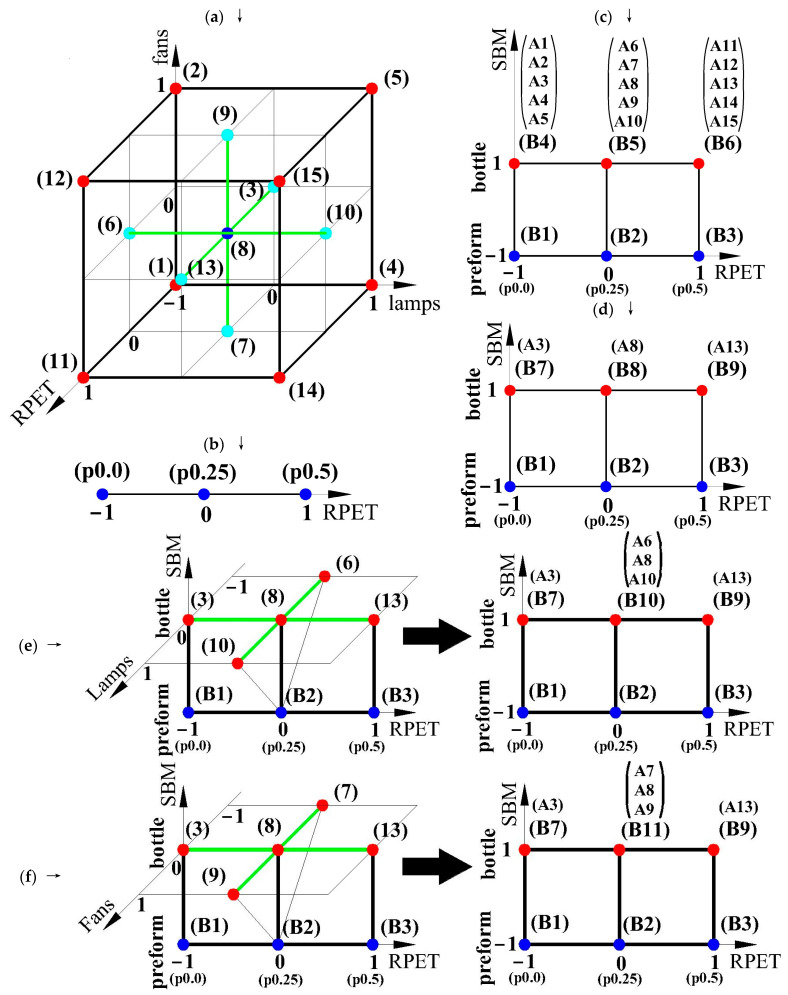
Graphic representation of the test plans of measurement series in Table 1: (**a**) CCF test plan for bottle material (numbers in parentheses correspond to the “A” series numbers); (**b**) test plan for a single-factor trivalent study of the impact of rPET content on the properties of preform material; (**c**) study plan for a two-factor study of the impact of the SBM process (bivalent factor) and rPET content (three-valued factor) on the “relative” properties of the bottles in relation to the properties of the preforms (for each rPET content, the lamp power and fan power settings were not fixed and changed (“ALL” plan)); (**d**) study plan of a two-factor study of the impact of the SBM process (bivalent factor) and rPET content (three-valued factor) on the “relative” properties of the bottles in relation to the properties of the preforms (for individual rPET content, the lamp power and fan power settings were fixed on the central values from the plan shown in Table 1 as A8 series (“RPET” plan)); (**e**) study plan for a two-factor study of the impact of the SBM process (bivalent factor) and rPET content (three-valued factor) on the “relative” properties of the bottles in relation to the properties of the preforms (for individual rPET content, the lamp power settings were changing, while the fan power settings were fixed to the central value from the plan shown in Table 1 as A8 series (“RPET + LAMPS” plan)); (**f**) study plan for a two-factor study of the impact of the SBM process (bivalent factor) and rPET content (three-valued factor) on the “relative” properties of the bottles in relation to the properties of the preforms (for individual rPET content, the fan power settings were changed, while the lamp power settings were fixed at the central value from the plan shown in Table 1 as A8 series (“RPET + FANS” plan)).

The examination of the microscopic and macroscopic features of the preform and bottle is carried out using 6 plans, summarized in Table 1 (a graphic representation of the test plans is shown in Figure A1 (Appendix A)). A detailed description of each plan is described in Supporting Information in Section S.1 (Table S1 shows the equations of calculating the main linear effects, main quadratic effects, and linear two-way interaction effects for the three-way, three-valued DOE).

It should be emphasized that compared to linear main effects, the geometric interpretation of linear two-way interactions depends on the value and sign of the individual effects for which the interaction is calculated. The influence of the value and sign of the linear two-way interaction AxB on the interpretation of the effect of Factor A depending on the level of Factor B, and vice versa, is summarized in Table S2. In Table S2, the equality (=) relationship between the effects was defined by the 5% tolerance range for the variability of standardized effects, i.e., if the 5% tolerance window for two effects overlaps, it was assumed that both effects are equal; Formula (S18) is given as an example for the analysis of the equality between the values of the “A” effect and the “B” effect. The analysis of the equality between the other effects is analogous.

Figure S1 (Supporting Information) shows the geometric interpretation of the main quadratic effect and the main linear effect in the case of a three-valued single-factor plan—Figure S1 also shows linear and quadratic equations and graphs approximating the measurements (based on linear multiple regression). Referring to the analysis of quadratic effects, four cases may occur depending on the statistical significance of the quadratic effect and its absolute value in relation to the absolute value of the linear effect: 1. no non-linearity (the quadratic effect is statistically insignificant); 2. non-linearity, but without a change in the sign of the trend changes in the dependent variable in terms of changes in the independent variable (the quadratic effect is statistically significant, but its absolute value is not greater than ¼ of the absolute value of the linear effect—condition (S6)); 3. non-linearity, in the absence of clear evidence of a change in the sign of the trend in changes in the dependent variable in terms of changes in the independent variable (the quadratic effect is statistically significant, but its absolute value is greater than ¼ but not greater than ½ the absolute value of the linear effect—condition (S7)); 4. non-linearity with a change in the sign of the trend of changes in the dependent variable in terms of changes in the independent variable (the square effect is statistically significant, and its absolute value is greater than ½ of the absolute value of the linear effect—condition (S8)).

**Table 1:** Test plans, independent and dependent variables, run order, and the number of samples for the measurement series, testing the microscopic and macroscopic features of bottles, preforms, and bottles relative to preforms (the SBM process); graphic representations of the test plans are shown in Figure A1 (Appendix A).

Measurement Series						Run order of Bottle Measurement Series ^6^	Number of Bottles	Independent Variables										Dependent Variables ***					
Bottle ^a^	Preform ^b^	SBM Process (Bottle vs. Preform)						RPET Content			Power of LAMPS			Power of FANS			FORM	Microscopic				Macroscopic	
		“ALL” ^c^	“RPET” ^d^	“RPET+ LAMPS” ^e,7^	“RPET + FANS” ^f,7^			(−1)	(0)	(1)	(−1)	(0)	(1)	(−1)	(0)	(1)		ρ ^1,2^	DSC ^1,2^	PALS ^1^	IV ^1^	TH ^3^ (I,II,III)	PRT ^4^
								0%	25%	50%	−10%	0%	10%	−5%	0%	5%		Number of Samples					
A1		B4				15	15	(−1)			(−1)			(−1)			Bottle **	1	1	1	1	5 × 3	10
A2						4	15	(−1)			(−1)			(1)				1	1	1	1	5 × 3	10
A3			B7	B7	B7	8	15	(−1)			(0)			(0)				1	1	1	1	5 × 3	10
A4						1	15	(−1)			(1)			(−1)				1	1	1	1	5 × 3	10
A5						14	15	(−1)			(1)			(1)				1	1	1	1	5 × 3	10
A6		B5		B10		13	15	(0)			(−1)			(0)				1	1	1	1	5 × 3	10
A7					B11	6	15	(0)			(0)			(−1)				1	1	1	1	5 × 3	10
A8			B8	B10		11	15	(0)			(0)			(0)				3 ^5^	3 ^5^	3 ^5^	3 ^5^	5 × 3	10
A9						10	15	(0)			(0)			(1)				1	1	1	1	5 × 3	10
A10				B10		3	15	(0)			(1)			(0)				1	1	1	1	5 × 3	10
A11		B6				12	15	(1)			(−1)			(−1)				1	1	1	1	5 × 3	10
A12						2	15	(1)			(−1)			(1)				1	1	1	1	5 × 3	10
A13			B9	B9	B9	9	15	(1)			(0)			(0)				1	1	1	1	5 × 3	10
A14						7	15	(1)			(1)			(−1)				1	1	1	1	5 × 3	10
A15						5	15	(1)			(1)			(1)				1	1	1	1	5 × 3	10
	p0.0	B1				-		(−1)			-			-			Preform *	(−1)	-	-	1	-	-
	p0.25	B2				-		(0)			-			-				(0)	-	-	3	-	-
	p0.5	B3				-		(1)			-			-				(1)	-	-	1	-	-

^1^ One bottle out of fifteen was randomly drawn from each series (except the A8 series) after the pressure resistance test (PRT). ^2^ The same sample was used first in the density study and then in the DSC study. ^3^ Five bottles out of fifteen were randomly drawn before the PRT (for each bottle, thickness (TH) measurement was made at three points on the circumference of the bottle at points I, II, III—Figure 2b). ^4^ Ten bottles out of fifteen were randomly drawn from each of measurement series. ^5^ Three bottles out of fifteen were randomly drawn from the A8 series after the PRT to evaluate the error. ^6^ The order of production of the bottle measurement series on the thermodynamically stabilized SBM machine (the thermodynamic stabilization procedure is described in another article [11]) was randomly generated. ^7^ In order to determine, for example, the effect of the heating lamp power on the change in microscopic properties of the bottle in relation to the preform, the effects obtained from the “RPET” plan and those from the “RPET + LAMPS” plan were compared, and if the analyzed effect for rPET content was statistically significantly (the difference was tested with the post hoc Bonferroni test [12]) greater for the “RPET + LAMPS” plan than for the “RPET” plan, it was concluded that the influence of heating lamps increases the effect of changing rPET content on the change in microscopic properties of the bottle in relation to the preform in the SBM process. * All preforms were made by GTX Hanex Plastic Sp. z o.o. (https://www.hanex.com.pl/en/preforms/ (accessed on 19 September 2024)). PET recyclate (rPET) in the form of granulate was obtained using the material recycling method to comply fully with Directive 94/62/EU (https://eur-lex.europa.eu/eli/dir/1994/62/oj (accessed on 19 September 2024)). The ^13^C NMR and ^1^H NMR tests (Figure 3) show that the preforms used, both containing and not containing rPET, showed high chemical purity and contained only PET. ** All bottles were manufactured using the SBM method using a Blueline 1HiTech blow molding machine from TES Sp. z o. o (Figure 2c). *** Summary of methods and measurement tools used in the study is summarized in Table 2. ^a–f^ A graphic representation of the test plans is shown in Figure A1 (Appendix A).

Table A1 (in Appendix A) displays the values of the SBM process parameters that remained constant throughout the tests and were not considered independent variables in the study. The power of heating lamps was altered by changing the general power of the oven by ±10%, while the power of cooling fans was altered by ±5% compared to the average value (Table 1).

### 2.2. Materials, Reagents, and Method Used

Table 2 provides a consolidated overview of the methodologies and instruments employed to measure specific dependent variables. In Appendix B, a detailed description of the measurement methods used is presented, i.e., nuclear magnetic resonance (NMR) spectroscopy (Appendix B.1); degree of crystallinity determined by DSC method (Appendix B.2); density determined by the gradient column method (Appendix B.3); the intrinsic viscosity determined by capillary viscometer (Appendix B.4); the free volume determined by positron annihilation lifetime spectroscopy (PALS) (Appendix B.5); thickness profile and pressure resistance test (bottle macroscopic properties) (Appendix B.6).

**Table 2:** The summary of methods and measurement tools used for individual dependent variables.

Features		Method	Measurement Tool	Meter Type	Sample	Maximum Measurement Tool Error *
Composition and chemical structure of PET preform samples ^a^		NMR	Bruker Avance III 500 MHz (Billerica, MA, USA)	digital meter	1 × 1 cm square sample cut out from preform III point (Figure 2a)	-
Dependent Variables	IV ^a^	Capillary viscometer	Automatic capillary viscometer type HVM 472 (Walter Herzog: Lauda-Königshofen, Germany)	digital meter	1 × 1 cm square sample cut out from preform III point, and from the corresponding place after SBM process, bottle base area III point (i.e., the place of most significant deformation during blowing)—Figure 2	$\Delta_{1}=\pm 0.001\;dL/g$ (maximum measurement error was assumed to be equal to the resolution of the measuring instrument)
	Degree of crystallinity^a^	DSC analysis	TA Inst Q20 microcalorimeter (TA Instruments: New Castle, DE, USA)	digital meter		$\Delta_{2}=\pm 2\%$
	Density ^a^	Measurement in accordance with ASTM D 1505-85 [26]	Gradient column	analog meter		$\Delta_{3}=\pm 8.3\cdot 10^{-5}\;g/{cm}^{2}$
	Free Volume ^b^	PALS	Conventional fast–fast coincidence apparatus [27]	digital meter		$\Delta_{4}=0.0001\;ns$ (measurement error related to the measurement of time τ_2_ and τ_3_) $\Delta_{5}=0.01\%$ (measurement error related to the intensity measurement I1 + I3 and I2)
	Thickness Profile ^c^	Measurement of bottle thickness at points I, II, III (Figure 2b)	Inductive sensor FH4-1MM 80-174-0300 (ElektroPhysik: Cologne, Germany)	digital meter	bottle	$\Delta_{6}=\pm 0.01\;mm$
	Pressure Resistance ^c^	Bottles burst test with water pressure	ABT-3100 PET Bottle Burst Pressure (CMC KUHNKE: Berlin, Germany)	digital meter	bottle	$\Delta_{7}=\pm 0.1\;bar$

^a^ Research conducted at the Center of Molecular and Macromolecular Studies (CBMM), Polish Academy of Sciences. Information about the materials used in the study: Phenol and 1,2-dichlorobenzene were purchased from Sigma-Aldrich (Sigma, St. Louis, MO, USA). All other chemicals of analytical grade were obtained from POCh-Gliwice (Poland). ^b^ Research conducted at the Faculty of Physics and Astronomy, University of Wrocław. ^c^ Research conducted at TES Sp. z o. o. (Poland). * A rectangular error distribution was assumed for the entire measurement range.

#### 2.2.1. The SBM Process

**Figure 2:**
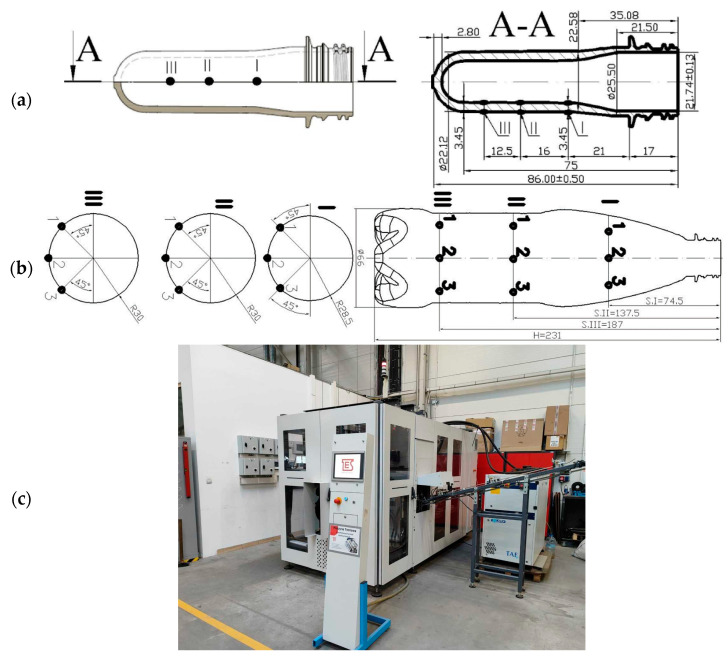
Dimensions with location of marking (point I, II, III) of (**a**) the preform, (**b**) the bottle used in the tests, and (**c**) a photo of the blow molding machine Blueline 1HiTech from TES Sp. z o. o.

The drawing and dimensions of the tested preform and bottle are shown, respectively, in Figure 2a and Figure 2b. Figure 2a,b also show the location of the places of cutting samples for measuring the microstructure properties of the preforms (point III) and the corresponding places after the SBM process of cutting samples for measuring the microstructure of bottles (point III). A photo of the blow molding machine Blueline 1HiTech is shown in Figure 2c. The machine has a linear oven for heating the preforms, which are transported continuously and heated using NIR heating lamps. Increasing the power of the heating lamps increases the temperature of the preform. Increasing the fan power increases the air flow in the heating oven, which flows around the preform. This results in a reduction in the temperature of the outer surface of the preform, meaning that the preform material is not in isothermal conditions (therefore, the research described in the paper is not based on ambiguous temperature measurements but on the unambiguous power of the heating lamps). In general, the purpose of using forced air flow in the heating oven is to protect the outer surface of the preform against overheating. If there is no forced air circulation (washing the preform), there will also be a significant temperature difference between the outer and inner surfaces of the preform—this phenomenon makes it practically impossible to form the bottle correctly. In this study, the phenomenon of the influence of fan power on the preform was simplified, and the influence of fan power on the temperature gradient between the outer and inner surfaces of the preform wall was not analyzed (increasing the cooling fan power reduces the preform temperature).

#### 2.2.2. Microcavitation Effect

Indirect estimation of the quantity of “oriented” and “rigid” amorphous phases in a multiphase PET model, inversely linked to the relaxation measure of the amorphous phase, is feasible using a two-phase PET model (known as the amorphous–crystalline model of PET). Based on measurements of the density and degree of crystallinity, the density of the amorphous phase (as a measure of the orientation of the amorphous phase) of the preform and bottle material can be determined by Equations (3)–(6) (methodology described in [11]). Based on the density of the amorphous phase, the microvitation effect (M.E.) was defined (Equations (1) and (2)).

(1) $$M.E.=\frac{\rho_{a}^{u}-\rho_{a}}{\rho_{a}^{u}}\pm \Delta_{M.E.}$$

(2) $$\Delta_{M.E.}=\sqrt{{\left(\frac{\partial M.E.}{\partial \rho_{a}}\cdot \Delta_{\rho_{a}}\right)}^{2}}=\sqrt{{\left(-\Delta_{\rho_{a}}\right)}^{2}}=\Delta_{\rho_{a}}$$

(3) $$\rho_{a}=\frac{\rho_{c}\cdot \rho \cdot \left(1-C_{DSC}\right)}{\rho_{c}-\rho \cdot C_{DSC}}\pm \Delta_{\rho_{a}}$$

(4) $$\Delta_{\rho_{a}}=\sqrt{{\left(\frac{{\rho_{c}}^{2}\cdot \left(1-C_{DSC}\right)}{{\left(\rho \cdot C_{DSC}-\rho_{c}\right)}^{2}}\cdot \Delta_{\rho }\right)}^{2}+{\left(\frac{\rho \cdot \rho_{c}\cdot \left(\rho -\rho_{c}\right)}{{\left(\rho \cdot C_{DSC}-\rho_{c}\right)}^{2}}\cdot \Delta_{C_{DSC}}\right)}^{2}}$$

(5) $$\Delta_{\rho }=k\cdot \sqrt{{\left(\frac{\Delta_{3}}{\sqrt{3}}\right)}^{2}+\frac{{\sigma_{\rho }}^{2}}{n_{\rho }}}$$

(6) $$\Delta_{C_{DSC}}=k\cdot \sqrt{{\left(\frac{\Delta_{2}}{\sqrt{3}}\right)}^{2}+\frac{{\sigma_{C_{DSC}}}^{2}}{n_{C_{DSC}}}}$$

where the crystallization degree of the polymer, measured by DSC, is denoted as $C_{DSC}$ [%]. The density of the PET sample, measured in a gradient column, is indicated as ρ [$g/{cm}^{3}$]. The density of the unoriented amorphous PET phase is $\rho_{a}^{u}$, which is 1.335 $g/{cm}^{3}$ [28], while the density of the crystalline phase of PET (perfect crystallites) is $\rho_{c}$, with a value of 1.455 $g/{cm}^{3}$ [28]. The extension factor is denoted as “k” (for p = 0.95, k = 1.96). The number of measurement repetitions is represented by n. The most probable value of the absolute error for the assumed confidence level of p = 0.95 is denoted by Δ. The standard deviation from the mean of measurements for the A8 series and preform containing 25% rPET content is represented by σ (see Table S4). $\Delta_{2}$ is TA Inst Q20 microcalorimeter measurement uncertainty (see Table 2); $\Delta_{3}$ is the uncertainty of density measurement in a gradient column filled with water solutions of calcium nitrate (see Table 2).

The parameter we have defined as M.E. (Equation (1)) is interpreted as a change in the porosity of the material at the level of free volume and not the formation of cavitation pores observed, for example, when stretching PP [29]. The measure of this parameter is the change in the density of the amorphous phase. It should be emphasized that the τ_3_ time from PALS analysis (Figure 4f) in samples from bottles is shorter than in preforms (this means that the average lifetime of the ortho-positronium and, therefore, the average pore size of the free volume is also smaller—Equation (A8)). In contrast, δτ_3_ (Figure 4f) and σ_3_ (Figure 4i) are greater for the bottle material than the preform. Such an effect can be interpreted as a change in the shape of the free volume pores (from statistically spherical) in the case of materials with an oriented amorphous phase. Then, the annihilation of ortho-positronium along the shorter axis of the ellipsoidal pores of the free volume is more likely than along the longer one, and at the same time, the density of the amorphous phase (as a measure to calculate by Equation (1) M.E. in Figure 4a) decreases. This means that the pore size measurement based on time τ_3_ is strongly sensitive to the pore shape [30]; however, the microcavitation testing technique based on the density of the amorphous phase (defined by Equation (1)) is not sensitive to the shape of the pores. Therefore, only the combination of these two techniques allows for a reliable interpretation of the shape and distribution of free volumes.

### 2.3. Statistical Analysis Methodology

The DOE analysis was performed together with the ANOVA statistical significance test (Table S3). All ANOVA tests were conducted for an arbitrarily assumed 5% probability of making a type I error [12,31,32], and all the test power for each effect was conducted for a 20% probability of making a type II error [12]. Detailed information about the statistical tools and equations used in the work is presented in Supporting Information in point S.1.

## 3. Research Results

The results of measurements of the physical and thermal properties of the preform and bottle material are summarized in Table S4; the lifetime and percentage intensity of subatomic antiparticles measured with the positron annihilation method of measuring the free volume of bottle and preform material and taking into account the dispersion σ_3_ are summarized in Table S5, while the lifetime and percentage intensity of subatomic antiparticles measured in positron annihilation method of measuring the free volume of bottle and preform material and taking into account the dispersion σ_3_ are summarized in Table S6. After removing outliers, the results of measurements of the thickness profiles of the bottle wall are summarized in Table S7, while the measurement results of the pressure resistance are summarized in Table S8. Table S9 presents the photos of two bottles from each measurement series, with extreme locations of the bottle cracking initiation points (after removing outliers)—the remaining bottles in the series cracked in the areas defined within the boundaries of the bottles shown.

A full statistical interpretation of research results is presented in the second part of the article [15]. The following contains only analysis related to the comparison of vPET and rPET and the impact of rPET content on preform and bottle microscopic and macroscopic properties.

### 3.1. ^13^C-NMR and ^1^H NMR Analysis of PET Preform Material

**Figure 3:**
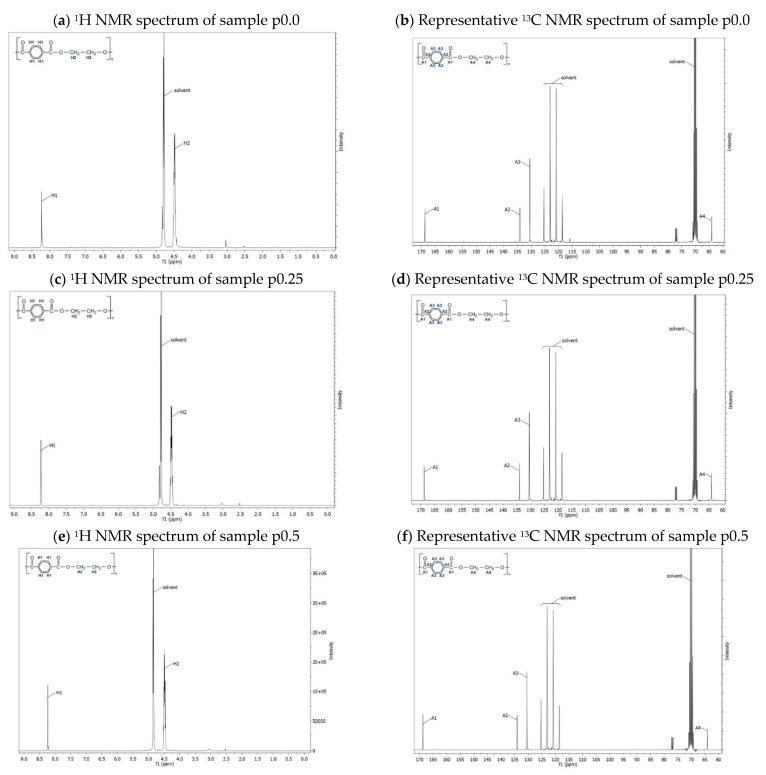
^13^C NMR and ^1^H NMR spectra for p0.0 (0% of rPET content), p0.25 (25% of rPET content), and p0.5 (50% of rPET content) preform material.

^13^C NMR and ^1^H NMR spectra for p0.0, p0.25, and p0.5 preform material are illustrated in Figure 3a–f, and the corresponding assignments of the peaks are marked (H1, H2, A1, A2, A3, A4) in the structural formulas.

In the ^1^H NMR spectra, the chemical shift at δ 8.22 ppm is the characteristic absorption band on the benzene ring (H1—shown in Figure 3a,c,e), while the chemical shift at δ 4.48 ppm corresponds to the proton (H2—shown in Figure 3a,c,e) in PET. The OCH2 resonances for PET are observed at 4.0–4.7 ppm (shown in Figure 3a,c,e) due to the influence of ester groups. The ^13^C NMR spectra of preform samples exhibit the resonances associated with the benzene ring (δ 134 ppm and δ 130 ppm, respectively, A2 and A3—shown in Figure 3b,d,f), with the methylene groups at δ 64 ppm (A4—shown in Figure 3b,d,f) and the carbonyl ester moieties at 168 ppm (A1—shown in Figure 3b,d,f) attributed to benzene counterparts.

Based on the intensity of the resonance peaks and the corresponding chemical shifts, it can be observed that all of the preforms marked as p0.0, p0.25, and p0.5 contain only polyethylene terephthalate [33,34]. No other components/additives were found in the analyzed preparations. It also means that the rPET preforms used in the research were characterized by high chemical purity.

### 3.2. Microscopic and Macroscopic Properties of the Preform and Bottle Material Research

**Figure 4:**
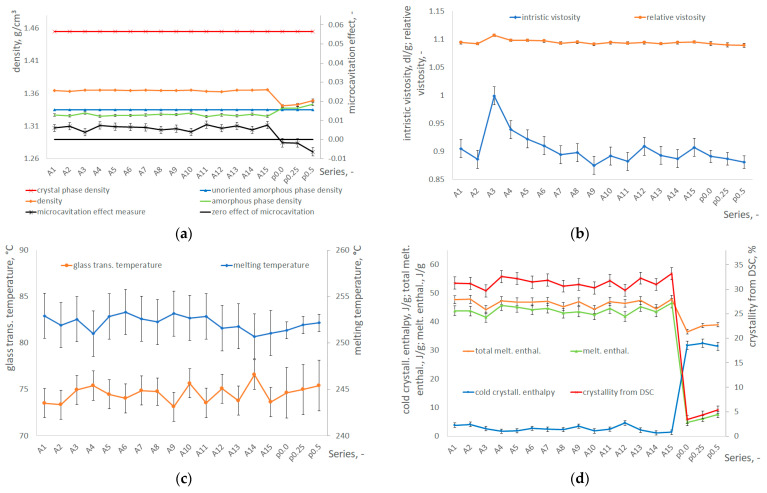

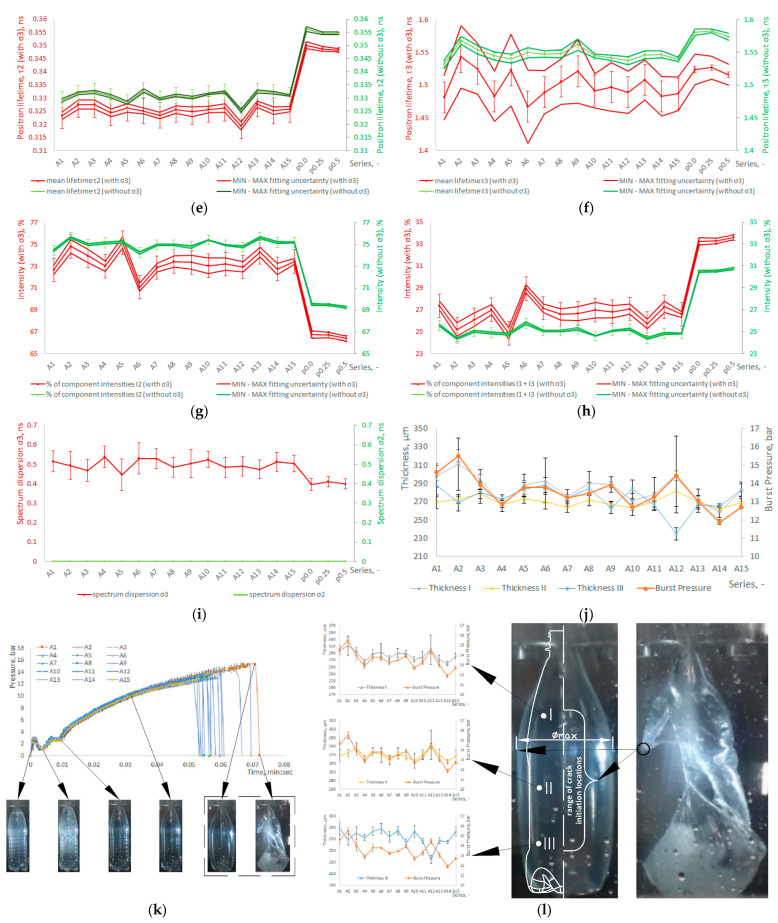
Results of the measurements of microscopic and macroscopic properties of preform and bottle material with bars of measurement uncertainty: (**a**) density, amorphous phase density (the non-crystalline phase density) against the crystalline phase density and the non-oriented amorphous phase density together with the quantification of M.E. (Formula (1)) against the zero M.E.; (**b**) the IV and relative viscosity; (**c**) the Tg and the Tm; (**d**) the degree of crystallinity of PET and the enthalpy of cold crystallization, total enthalpy of melting and enthalpy of hot crystallization; (**e**) mean positron lifetime (τ_2_) with the spectrum fitting uncertainty range of the adopted fitting model; (**f**) mean ortho-positronium lifetime (τ_3_) with the spectrum fitting uncertainty range resulting from the adopted fitting model; (**g**) the intensity of the positron component related to the crystal space (I_2_) with the spectrum fitting uncertainty range resulting from the adopted fitting model; (**h**) the o-Ps intensity (I_1_ + I_3_) with spectrum fitting uncertainty range resulting from the adopted fitting model; (**i**) results of measurements of dispersion parameters (σ_2_ and σ_3_) of the analyzed spectrum in PET; (**j**) pressure resistance and thickness of the bottle; (**k**) the pressure changes in the bottle during the pressure resistance test; (**l**) shape of the bottle (with thickness measurements) just before and after fracture, showing the location of fracture initiation for the maximum diameter of the bottle shown, and the range of locations at which fracture initiated throughout the whole experiment (see Table S9).

Figure 4a–d show the measurement results of the physical (density according to Formula (A2), intrinsic and relative viscosity according to Formulas (A4) and (A5)) and thermal (glass transition and melting temperatures; melting enthalpy; degree of crystallinity according to Formula (A1)) properties of PET bottles and preforms, determined by using density gradient column, capillary viscometer, and differential scanning calorimetry (DSC). Microcavitation (M.E.) was defined by Formula (1).

Figure 4e–i show the results of the analysis of the positron annihilation lifetime spectra described by mean lifetimes: the intermediate lifetime τ_2_, due to free positron annihilation, and the longest lifetime τ_3_, due to ortho-positronium (o-Ps) annihilation with their corresponding intensities I_2_ and I_1_ + I_3_, as well as the dispersion parameters σ_2_ and σ_3_.

All PALS graphs show the results both for the model taking into account the σ_3_ dispersion of the spectrum and for the model not taking it into account (with and without σ_3_ dispersion). The first one, referred to as “with dispersion”, assumes that each measured spectrum can be described using three components which correspond to the p-Ps annihilation (characterized by τ_1_ = 125 ps and I_1_), the free positron annihilation (characterized by τ_2_ and I_2_), and the ortho-positronium (o-Ps) pick-off annihilation (characterized by τ_3_, σ_3_, and I_3_). The I_1_/I_3_ ratio was fixed at 1/3. At the same time, the longest component was described using continuous component yielding the τ_3_ average lifetime and the dispersion of lifetimes σ_3_. In the polymeric systems, the distribution of τ_3_ is related to the distribution of the free volume. The second model, referred to as “without dispersion”, is similar to the first one but assumes that σ_3_ is zero. Both models are commonly used to describe PALS spectra measured for polymers [35,36].

The most important parameter is τ_3_, which is proportional to the average volume of free spaces (voids) in the material [30]. The higher the value of τ_3_, the larger the average dimensions of free spaces, and thus, the greater the potential M.E. In the model with dispersion, it is possible to estimate the size distributions of the voids with the average volume related to τ_3_ and the dispersion σ_3_. The average lifetime of τ_2_ is responsible for the free annihilation in the material; it is not related to the free spaces in the tested material, and often in polymers, it is related to the positron annihilation in the crystalline parts. The I_1_ + I_3_ parameter (i.e., the intensity of the positron component) determines how many positrons entering the material create positrons in free spaces. The model without dispersion allows for a more reliable estimation of small changes in the average void volume in the material but does not allow for the formation of the distributions of these voids—it can only estimate their average size.

The error bars shown in Figure 4a–i are the result of the standard deviation of the mean calculated for three repetitions of the p0.25 (25% of rPET content) and A8 series measurements, while the continuous lines in the vicinity of the mean shown in Figure 4e–h define the fitting uncertainty of the analyzed spectrum (δτ_2_, δτ_3_), calculated as the root of the sum of the squares of the fitting uncertainties (which were obtained by fitting the spectra for each sample) and the standard deviation of the mean of these fitting uncertainties calculated for three replicates measurements.

Figure 4j shows the results of the measurements of the bottle burst pressure (pressure resistance) with the measurement uncertainty after removing outliers and the results of bottle wall thickness measurements at each measurement point (shown in Figure 2b) for the entire circumferential cross-section I, II, and III (treated as one thickness measurement point) for each measurement series, along with the measurement uncertainty bars after removing outliers. Figure 4k shows the pressure changes in the bottle while filling with water during the pressure resistance test, including the uncertainty of pressure measurements. The mean pressure for a given measurement series was calculated as averages for 10 randomly selected bottles from a series of 15 bottles. Figure 4l shows the shape of the bottle (with thickness measurements) just before and after fracture, showing the location of fracture initiation for the maximum diameter of the bottle shown and the range of locations at which fracture was initiated throughout the whole experiment (see Table S9).

Figure 4a–d and Figure S5 (standardized results of measurements) show the measurement results of the physical and thermal properties of PET bottles and preforms, which indicate the following:

1. The density of PET preforms was similar to each other and significantly lower than the densities of the PET bottle fragments. As shown by calorimetric studies, this effect was caused by a significant difference in the degree of crystallinity between the preforms and the analyzed bottle fragments. At the same time, there was no visual difference in the density of fragments obtained from the various parts of the bottles.
2. There was also no significant influence of the thermomechanical history of the forming process on the Tg and the Tm of the analyzed samples. However, significant differences were observed in the phase structure. The PET preforms are samples with negligible crystallinity (degree of crystallinity in the range of 3.4–5.4%), while bottle fragments as a result of stretch blow molding were characterized by the maximum value of the degree of crystallinity of 29–33% (when heated in the DSC apparatus, the enthalpy of the cold crystallization process for the bottle fragments was only 1–4.5 J/g).
3. The SBM process strongly influenced the density of the amorphous phase (to be precise, the density of the non-crystalline phase). In the case of preforms, the density of the amorphous phase is slightly higher than the density of the non-oriented amorphous phase, which can be explained by the increased content of trans conformation in the amorphous phase [37]. In the case of samples obtained from formed bottles, the density of the amorphous phase was significantly lower compared to the density of the non-oriented amorphous phase of PET, which can be explained by M.E. occurring in the SBM process [12] related to the content of the free volume in the amorphous phase.
4. The intrinsic viscosity (IV) for PET preforms was in the range of 0.881–0.891 dL/g. In the case of the remaining analyzed samples (fragments of bottles), the value of this parameter was similar or slightly higher. This finding proves that no measurable thermomechanical degradation of the polymer material occurred during the bottle-forming process. At the same time, the increase in the IV of the bottles in relation to the IV of the preform can be explained by the fact that the bottle molding process induced a small amount of post-condensation, thereby increasing the molecular weight [38].

Due to the occurrence of SSPC phenomena, the microstructure analysis based on DSC thermograms and density measurements was extended to include PALS tests, which, however, correlate with the results obtained from the DSC and density analysis. The analysis of the PALS measurement results shown in Figure 4e–i and Figure S6 (standardized results of measurements) led to the following conclusions:

1. The lifetime τ_2_ (associated with annihilation in the crystalline phase) for the preform material is significantly greater than for the bottle material. On the other hand, the intensity of the I_2_ component in the preform material is significantly lower than in the bottle material. From this, it can be concluded that there are significantly more crystal structures in the bottle material than in the preform material, which coincides with the degree of crystallinity determined by DSC shown in Figure 4d.
2. The lifetime τ_3_ (proportional to the volume of voids in the entire material) for the preform material is slightly greater than for the bottle material. Also, the intensity of the I_1_ + I_3_ component in the preform material is significantly higher than in the bottle material. There are higher average dimensions of free volume in the preform material than in the bottle material, mainly due to the orientation of the amorphous phase (thus reducing the distance between macromolecules) and its further ordering to the crystalline phase during the SBM process.
3. The scattering of the spectrum dispersion σ_3_ in the preform material is smaller than in the bottle material. It follows that the free volumes in the preform material have a “more spherical shape” than in the bottle material. The slightest scattering of σ_3_ (the most “spherical” free volume shapes) is for a preform material made of vPET (without rPET content).

Figure 4j–l and Figure S7 (standardized results of measurements) show the results of the macroscopic properties of bottles (pressure resistance and thickness in points I, II, III), which indicate the following:

1. The greater the thickness at points I and II (bottle label region), the greater the pressure resistance (this cannot be said for point III).
2. The most important macroscopic parameter influencing pressure resistance is thickness between points I and II because in the area of these points, the smallest bottle thickness is obtained, and the highest diameter is achieved during the pressure resistance test (the stress in the cylindrical tank wall caused by the pressure inside the cylindrical tank increases with the cylindrical tank diameter and also increases rapidly with the decrease in the cylindrical tank wall thickness caused by the stretching from the increase in the cylindrical tank diameter [39]), and thus in these locations the cracking starting points occur for most of the bottles in every series (except for series A6, for which the cracking starting point was around point III for most of the bottles).
3. The thickness at point III is smaller than the thickness at point I and comparable to point II, but there is no such relationship between thickness at point III and pressure resistance. The exception for point III is the effect of rPET content—increasing the rPET content causes a decrease in the thickness at point III (especially for series A12—the coldest preform) and also a decrease in the pressure resistance of the bottle, but the place where the crack started does not move towards the bottom of the bottle (which in turn was observed in SBM process [4]).
4. It indicates that at point III, the greatest phase transformations occur, which are related to the orientation of the amorphous phase as a result of bottle deformation in the SBM process.

Due to the fact that for different series, and even for different bottles in one series, the crack initiation point was located differently (see Table S9), it was ambiguously to adopt a methodology for selecting the location for measuring the bottle microstructure, but it was noted that for high rPET contents (for A11, A12 and A13 series), the thickness at point III is smaller than the thickness at point I and II, and it does not corresponds to pressure resistance. This may indicate that at point III, the greatest phase transformations occur, which are related to the orientation of the amorphous phase as a result of bottle deformation in the SBM process. This observation was the basis for adopting point III of the bottle as the place of cutting out samples for the analysis of the bottle microstructure. Additionally, the preform’s bottom (the “gate” area) experiences higher temperatures (see Table A1), leading to a faster crystallization process before blowing compared to the label part of the preform. Consequently, the base of the bottle exhibits higher crystallization than the label part, and M.E. during blowing is more pronounced.

The acceptance of point III as the location for measuring phase transformations is also supported by the fact that during the pressure resistance test, the place around point III was subject to the smallest deformations, and therefore, there was no deformation of the free volumes. Thus, this point is the place where the structure of the bottle material is best reproduced immediately after the SBM process. In particular, regarding the shape of the free volumes (free volumes are strongly modified in deformed semicrystalline polymers even below the Tg, in particular during destruction, because small free volumes merge into larger volumes [30]), other studies have shown that during the blow molding of bottles with the addition of rPET, the area around the bottom is torn [4]. Therefore, in further consideration, it was assumed that the phase transformations determined on the basis of samples cut in point III are representative for comparison with pressure resistance, mainly in the case of the analysis of the rPET content.

Furthermore, changes in the independent variables affect the entire volume of the preform material in a uniform manner. Therefore, it was assumed that microstructure measurements taken at the point with the strongest phase transformations would reflect changes for all points of the bottle volume as a result of changes in the independent variables after the SBM process (the values of the individual dependent variables of the microstructure would differ at each point of the bottle volume, but it was assumed that since the independent variables affect all points of the preform material uniformly, the sign of the change trend of the dependent variables would also be the same at each point of the bottle volume).

## 4. Summary

The experimental research presented in this paper and the results of the microstructure measurements of the preform material and the bottle produced in the SBM process confirm that both the microcavitation process and the solid state post-condensation process occur in PET material of bottles produced by the SBM process.

The microcavitation and post-condensate processes have an impact on the dimensions and shape of the free volumes of pores, which in turn are strongly related to the cracking mechanism of semicrystalline polymers during mechanical deformation [30]. However, the microstructural changes occurring in the SBM process are exceptionally complex [14]. Within the assumed variability range, the effect of rPET content, power of heating lamps, and power of cooling fans in the SBM process on the microstructure of the bottle material is strongly intertwined, in particular in terms of the impact on the density of the bottle material (because the density of the material is affected by the amount of free volume, the amount of amorphous phase, the amount of non-crystalline mesophase, and the amount of crystalline phase). In order to draw statistics reliable conclusions, it is necessary to conduct a statistical analysis of the obtained research results. The entire statistical analysis, i.e., the analysis of statistically significant linear effects, two-factor cross effects, and quadratic effects, is presented in the second part of the paper [15].

## Data Availability

The original contributions presented in the study are included in the article and Supplementary Material, further inquiries can be directed to the corresponding author.

## Supplementary Materials

The following supporting information can be downloaded at: https://www.mdpi.com/article/10.3390/ma17215233/s1, Figure S1: The main quadratic effect, where: (a) geometric interpretation of the main linear effect (e.L. = τ) and the main quadratic effect (e.Q. = τ^2^) for a three-valued single-factor plan with standardized values of the independent variable from −1 to 1 (i.e., −1, 0, 1), when condition (S8) is met; (b) presentation of the method of interpretation of the quadratic non-linear effect in the case of the unfulfilled condition of the occurrence of an extreme (Equation (S6) is met) for a single-factor three-valued plan with standardized values of the independent variable (−1,0,1); Figure S5: Summary of standardized results of measurements of material density, degree of crystallinity, intrinsic viscosity, and microcavitation in point III (see Figure 2a,b) for the series of bottles (A1–A15) and preforms (p0.0, p0.25, p0.5); Figure S6: Summary of standardized results of PALS analysis in point III (see Figure 2a,b) for the series of bottles (A1–A15) and preforms (p0.0, p0.25, p0.5); Figure S7: Summary of standardized results of pressure resistance and bottle material distribution (TH-I, TH-II, TH-III, i.e. thickness in point I, II, III) measurements (see Figure 2b) for the series of bottles (A1–A15); Table S1: Plan of a three-way, three-valued experiment (Figure S2a) with the method of calculating the main linear effects (τ, β, γ), main quadratic effects (τ^2^, β^2^, γ^2^), and linear two-way interaction effects ((τβ), (τγ), (βγ)); Table S2: Influence of the value and sign of the two-way interaction AxB on the interpretation of the Factor A effect de-pending on the level of Factor B, and vice versa; Table S3: Summary of the formulas needed to perform ANOVA for an incomplete 3-factor, 3-valued plan, including three linear main effects, three quadratic main effects, and three linear effects of two-way interaction; Table S4: Presentation of research results on the physical and thermal properties of materials for bottles and preforms (measurement series are related to Table 1), where (a) is mean value; (b) is measurement uncertainty; (c) is sample size; Table S5: Presentation of research results on the lifetime and percentage intensity of subatomic particles measured in positron annihilation method of measuring the free volume of bottle and preform material, taking into account the dispersion σ₃ (measurement series are related to Table 1), where (a) is mean value; (b) is measurement uncertainty; (c) is sample size; Table S6: Presentation of research results on the lifetime and percentage intensity of subatomic particles measured in positron annihilation method of measuring the free volume of bottle and preform material after removal of the dispersion σ₃ (measurement series are related to Table 1), where (a) is mean value; (b) is measurement uncertainty; (c) is sample size; Table S7: Summary of the results of the study of the thickness profiles (measurement series are related to Table 1), where (a) is the mean value; (b) is the measurement uncertainty; (c) is the sample size (after removing outliers*); Table S8: Summary of the results of the study of the pressure resistance (measurement series are related to Table 1), where (a) is the mean value; (b) is the measurement uncertainty; (c) is the sample size (after removing outliers*); Table S9: Photos of two bottles from each measurement series, with extreme locations of the bottle cracking initiation points (after removing outliers)—the remaining bottles in the series cracked in the areas defined within the boundaries of the bottles shown; [11,12].

## Appendix A

**Table A1:** SBM process parameters that remained constant throughout the tests and were not considered independent variables in the study.

SBM Process Parameters		Power of Individual Heating Lamps in the Oven (for Series A8 in Table 1)
Stretching rod speed	1.5 m/s	01: 43.4% (near thread) 02: 54.2% 03: 40.6% 04: 54.2% 05: 33.8% 06: 74.5% (near “gate”) 07: 0.0% 08: 0.0% 09: 0.0% General power of the oven: 65.0% Absolute installed power of the oven: 16.8 kW
Initial blow start delays relative to the position of the stretching rod	5 mm before the rod touches the preform	
Pre-blow air pressure	8.0 bar	
Pre-blow time	0.09 s	
Main blow start delays relative to the position of the stretching rod	10 mm from the end (bottom) of the bottle	
Main blow air pressure	35 bar	
Main blow time	0.72 s	
Mold temperature	10 °C	
Average power of cooling fans (for series A8 in Table 1)	1.1 kW	

## Appendix B

### Appendix B.1. Nuclear Magnetic Resonance (NMR) Spectroscopy

In order to identify the composition and chemical structure of the analyzed PET preform samples, nuclear magnetic resonance (NMR) spectroscopy was performed using a Bruker Avance III 500 MHz spectrometer (Bruker Corporation: Billerica, MA, USA). The 1H NMR and 13C NMR spectra were performed at 298 K for samples prepared by dissolving 100 mg of the sample in 1.5 mL of 1,1,1,3,3,3-hexafluoro-2-propanol and 0.5 mL of deuterated chloroform (CDCl_3_). The total acquisition time for one sample was approximately 14 h.

### Appendix B.2. Degree of Crystallinity Determined by DSC Method

Thermal analysis of the tested materials was carried out using a DSC apparatus (Q20, Thermal Analysis) calibrated with indium. Samples of 6–9 mg were placed in airtight aluminum pans. Thermograms were recorded under heating at a constant rate of 10°/min in a nitrogen atmosphere and a temperature range of 0–300 °C. The degree of crystallinity was determined according to the Formula (A1).

(A1) $$C_{DSC}=\frac{\Delta h}{\Delta {h_{f}}^{o}}\pm \Delta_{C_{DSC}}$$

where $C_{DSC}$ is the weight crystallization degree of the polymer measured by DSC; Δh is the melting enthalpy minus the cold crystallization enthalpy of the sample, [J/g]; ${{\Delta h}_{f}}^{o}$ is the melting enthalpy of a perfect PET crystal, ${{\Delta h}_{f}}^{o}=140\;J/g$ [40].

### Appendix B.3. Density Determined by the Gradient Column Method

The determination of the density of the PET samples was carried out using the gradient column method in accordance with the ASTM D 1505-85 [26]. The method of measurement consisted of comparing the level of immersion of a sample of the tested material in a gradient column filled with aqueous solutions of calcium nitrate of different densities (in the range of 1.32–1.42 g/cm^3^), the density of which should be approximately equal to the lowest required density. After wetting with the immersion liquid, the sample was introduced into the column under the surface of the liquid and left in the column until equilibrium was reached. Measurements were carried out at 25 °C. The density of the tested samples was calculated on the basis of the height of the draft levels at which the samples remained at equilibrium, using Equation (A2). Due to the fact that density is measured indirectly, to calculate the error of density measurement, Formula (A3) should be used; however, the measurement uncertainty $\Delta_{a}$, $\Delta_{b}$, $\Delta_{x}$, $\Delta_{y}$, and $\Delta_{z}$ was not known. Therefore, a simplified Formula (5) was adopted to calculate the density measurement error.

(A2) $$\rho =a+\left[\frac{\left(x-y\right)\cdot \left(b-a\right)}{\left(z-y\right)}\right]\pm \Delta_{\rho }$$

(A3) $$\begin{array}{c} \Delta_{\rho }=\sqrt{{\left(\frac{\partial \rho }{\partial a}\cdot \Delta_{a}\right)}^{2}+{\left(\frac{\partial \rho }{\partial b}\cdot \Delta_{b}\right)}^{2}+{\left(\frac{\partial \rho }{\partial x}\cdot \Delta_{x}\right)}^{2}+{\left(\frac{\partial \rho }{\partial y}\cdot \Delta_{y}\right)}^{2}+{\left(\frac{\partial \rho }{\partial z}\cdot \Delta_{z}\right)}^{2}}=\\ =\sqrt{{\left(\Delta_{a}\right)}^{2}+{\left(\frac{x-y}{z-y}\cdot \Delta_{b}\right)}^{2}+{\left(\frac{b-a}{z-y}\cdot \Delta_{x}\right)}^{2}+{\left(\frac{\left(a-b\right){\left(z-y\right)}^{2}+\left(x+y\right)\left(b-a\right)}{{\left(z-y\right)}^{3}}\cdot \Delta_{y}\right)}^{2}+{\left(\frac{\left(y-x\right)\left(b-a\right)}{{\left(z-y\right)}^{2}}\cdot \Delta_{z}\right)}^{2}} \end{array}$$

where ρ is the density of the tested samples, [g/cm^3^]; a and b are the density of the two calibrated floats, [g/cm^3^]; y and z are the draft levels of floats a and b, [mm]; x is the immersion level of the test sample, [mm].

### Appendix B.4. The Intrinsic Viscosity Determined by Capillary Viscometer

The intrinsic viscosity was determined using an automatic capillary viscometer HVM 472 (Walter Herzog: Lauda-Königshofen, Germany) and a mixture of phenol/1,2-dichlorobenzene prepared in the ratio 1:1 [41]. The determination consisted of measuring the flow rate of the polymer/solvent solution, previously thermostated at 25 °C, through the capillary of the viscometer. The concentration of the tested PET samples in the solution was about 0.1 g/dL. The flow time of the phenol/1,2-dichlorobenzene solvent was 91.12 s. The relative viscosity η_r_ of the polymer solution) was calculated using Formula (A4). The intrinsic viscosity [η] of the tested polymers was calculated using the Solomon–Ciuta Equation (A5) [42,43]. Due to the fact that the intrinsic viscosity is measured indirectly, the calculation of the intrinsic viscosity measurement error should be calculated using Formula (A6); however, the measurement uncertainty for the flow time of the polymeric material solution ($\Delta_{t}$), the measurement uncertainty for the time solvent flow ($\Delta_{t_{0}}$), and polymer concentration measurement uncertainty ($\Delta_{c}$) were unknown. Therefore, the calculation of the intrinsic viscosity measurement error was based on a simplified Formula (A7).

(A4) $$\eta_{r}=\frac{t}{t_{0}}$$

where t is the flow time of the polymeric material solution, [s]; to is the solvent flow time, [s].

(A5) $$\left[\eta \right]=\frac{{\left[2\left(\eta_{s}-ln\eta_{r}\right)\right]}^{0.5}}{c}\pm \Delta_{\left[\eta \right]}=\frac{\sqrt{2\left(\frac{t}{t_{0}}-1-ln\left(\frac{t}{t_{0}}\right)\right)}}{c}\pm \Delta_{\left[\eta \right]}$$

(A6) $$\begin{array}{c} \Delta_{\left[\eta \right]}=\sqrt{{\left(\frac{\partial \left[\eta \right]}{\partial t}\cdot \Delta_{t}\right)}^{2}+{\left(\frac{\partial \left[\eta \right]}{\partial t_{0}}\cdot \Delta_{t_{0}}\right)}^{2}+{\left(\frac{\partial \left[\eta \right]}{\partial c}\cdot \Delta_{c}\right)}^{2}}=\\ =\sqrt{{\left(\frac{t-t_{0}}{t_{0}\cdot t\cdot c\cdot \sqrt{2\left(\frac{t}{t_{0}}-1-ln\left(\frac{t}{t_{0}}\right)\right)}}\cdot \Delta_{t}\right)}^{2}+{\left(\frac{{t_{0}}^{2}-t\cdot t_{0}}{{t_{0}}^{3}\cdot c\cdot \sqrt{2\left(\frac{t}{t_{0}}-1-ln\left(\frac{t}{t_{0}}\right)\right)}}\cdot \Delta_{t_{0}}\right)}^{2}+{\left(\frac{-\sqrt{2\left(\frac{t}{t_{0}}-1-ln\left(\frac{t}{t_{0}}\right)\right)}}{c^{2}}\cdot \Delta_{c}\right)}^{2}} \end{array}$$

(A7) $$\Delta_{\left[\eta \right]}=k\cdot \sqrt{{\left(\frac{\Delta_{1}}{\sqrt{3}}\right)}^{2}+\frac{{\sigma_{\left[\eta \right]}}^{2}}{n_{\left[\eta \right]}}}$$

where [η] is the intrinsic viscosity; η_r_ is the relative viscosity; η_s_ is the specific viscosity ($\eta_{s}=\eta_{r}-1$); c is polymer concentration [g/mL]; $\Delta_{1}$ is HVM 472 (Walter Herzong) capillary viscometer measurement uncertainty (see Table 2); n is the number of repetitions of measurements; $\sigma_{\left[\eta \right]}$ is the standard deviation from the mean of measurements of intrinsic viscosity for the A8 series and preform with 25% rPET content (Table S4).

### Appendix B.5. The Free Volume Determined by Positron Annihilation Lifetime Spectroscopy (PALS)

The assessment of free volume in both preform and bottle materials relied on PALS measurements, specifically at point III of the bottle and point III of the preform. These measurements were conducted at room temperature using a conventional fast–fast coincidence setup [27]. A droplet of ^22^Na from a carrier-free neutral solution was used as the positron source, dried onto two identical Hostaphan films, and bonded together. The source activity approached 1 MBq, with a spectrometer resolution of approximately 300 ps. Positrons emitted from the ^22^Na source were implanted into the surrounding samples and annihilated with electrons after undergoing thermalization, with an exposure time of 24 h for each sample.

Positrons introduced into a material typically thermalize within about 10 ps by interacting with localized electric fields within the material [22,44]. The positively charged positron undergoes scattering due to repulsion by the ionic cores of atoms until it eventually annihilates with an electron. If a positron randomly migrates into a void during its trajectory of 10–1000 μm, it gets “trapped” by the positive ionic cores on the void’s surface [22]. The trapped positrons have significantly longer lifetimes compared to other positrons, making this technique highly sensitive to voids of various sizes. When positrons annihilate with valence or core electrons (free or bulk annihilation), the mean positron lifetime attributed to this process typically ranges from 100 to 500 ps.

In molecular materials like polymers, some positrons may form positronium (Ps), which is a metastable bound state of a positron and an electron [35,36]. Positronium exists in two spin states: para-positronium (p-Ps) with antiparallel spins and ortho-positronium (o-Ps) with parallel spins. The relative abundance of these spin states is 1:3. The o-Ps has a self-annihilation lifetime of 142 ns, significantly longer than the p-Ps lifetime of 125 ps. However, when o-Ps is formed in condensed matter, its lifetime reduces to a few nanoseconds (typically 1 to 10 ns) due to a pick-off process, where the positron in o-Ps annihilates with one of the surrounding electrons with opposite spin.

According to the Tao–Eldrup model [45,46], the o-Ps pick-off annihilation lifetime (τ_3_) is directly related to the radius (r) of the spherical hole (free volume pore) in which it is situated, described by Formula (A8).

(A8) $$\tau_{3}\left[ns\right]=0.5{\left[1-\frac{r}{r+\Delta r}+\frac{1}{2\pi }\sin\left(\frac{2\pi r}{r+\Delta r}\right)\right]}^{-1}$$

Here, the term 0.5 ns stands for the inverse of the spin-averaged Ps annihilation rate, and Δr = 0.166 nm signifies the penetration depth of the Ps-wave function into the walls of the void.

The LT-9.0 software fitting function (A9) was utilized to analyze the obtained PALS spectra.

(A9) $$N\left(x\right)=\int_{0}^{\infty }P(x-t)\sum I_{i}F_{i}\left(t\right)dt$$

where P in Function (A9) denotes the instrumental resolution function, I_i_ stands for the relative intensity of the i-th component in the spectrum, and F_i_ can take the form of discrete or continuous components. The discrete component is given by Formula (A10).

(A10) $$F=\frac{1}{\tau }exp\left(-\frac{t}{\tau }\right)=\lambda exp\left(-\lambda t\right)$$

where λ and τ denote the positron annihilation rate and the positron lifetime, respectively. The continuous component comprises an ongoing sum of decay curves defined by a log-normal distribution Ln(λ), which corresponds to a Gaussian distribution on the logarithmic scale of lifetimes [47], as expressed by Formula (A11).

(A11) $$F\left(t\right)=\int_{0}^{\infty }L_{n}(\lambda )\lambda exp\left(-\lambda t\right)d\lambda =\frac{1}{{\lambda \sigma }_{0}\sqrt{2\pi }}\int_{0}^{\infty }exp\left[-\frac{{ln}^{2}\left(\lambda \tau_{0}\right)}{2\sigma_{0}^{2}}\right]\lambda exp\left(-\lambda t\right)d\lambda$$

In Equation (A11), σ_0_ denotes the variation in the distribution while τ_0_ is the center of the τ distribution. Bearing in mind that the distribution in log-Gaussian form is not symmetric, the mean positron lifetime can be determined using the function $\tau =\tau_{0}exp\left(\frac{\sigma_{0}^{2}}{2}\right)$, and the dispersion of lifetimes (standard deviation of the distribution) is given by $\sigma =\tau \sqrt{exp\left(\sigma_{0}^{2}-1\right)}$ [47].

The obtained PALS spectra were analyzed using two models. The first one, referred to as “with dispersion”, assumes that each measured spectrum can be described using three components which correspond to the p-Ps annihilation (characterized by τ_1_ = 125 ps and I_1_), the free positron annihilation (characterized by τ_2_ and I_2_), and the o-Ps pick-off annihilation (characterized by τ_3_, σ_3_, and I_3_). The I_1_/I_3_ ratio was fixed at 1/3. Additionally, the longest component was described using a continuous component yielding the τ_3_ mean lifetime and dispersion parameter σ_3_. In the polymeric systems, the distribution of τ_3_ is related to the distribution of the free volume. The second model, referred to as “without dispersion”, is similar to the first one but assumes that σ_3_ is zero. Both models are commonly used to describe PALS spectra measured for polymers [35,36].

The most important parameter is τ_3,_ which is proportional to the average volume of voids in the material. The higher τ_3_, the larger the free spaces (and therefore, the greater the potential effect of microcavitation calculated according to Formula (1)). In the model with dispersion, it is possible to estimate the size distributions of the voids with the average volume related to τ_3_ and the dispersion σ_3_. The mean lifetime of τ_2_ is responsible for the free annihilation in the material; it is not related to the free spaces in the tested material, and in polymers, it is often related to the positron annihilation in the crystalline parts. The I_1_ + I_3_ parameter determines how many positrons introduced into the material create Ps in free spaces. The model without dispersion allows for a more reliable estimation of small changes in the average void volume in the material but does not allow one to determine the distributions of these voids. It can only be used to calculate their average size. The uncertainty of time measurements τ_2_ ($\Delta_{\tau 2}$), τ_3_ ($\Delta_{\tau 3}$), and the uncertainty of percentage intensity I_1_ + I_3_ ($\Delta_{I1+I3}$), and I_2_ ($\Delta_{I2}$) were estimated based on Formulas (A12), (A13), (A14), (A15), respectively.

(A12) $$F\Delta_{\tau 2}=k\cdot \sqrt{{\left(\frac{\Delta_{4}}{\sqrt{3}}\right)}^{2}+\frac{{\sigma_{\tau 2}}^{2}}{n_{\tau 2}}}$$

(A13) $$\Delta_{\tau 3}=k\cdot \sqrt{{\left(\frac{\Delta_{4}}{\sqrt{3}}\right)}^{2}+\frac{{\sigma_{\tau 3}}^{2}}{n_{\tau 3}}}$$

(A14) $$\Delta_{I1+I3}=k\cdot \sqrt{{\left(\frac{\Delta_{5}}{\sqrt{3}}\right)}^{2}+\frac{{\sigma_{I1+I3}}^{2}}{n_{I1+I3}}}$$

(A15) $$F\Delta_{I2}=k\cdot \sqrt{{\left(\frac{\Delta_{5}}{\sqrt{3}}\right)}^{2}+\frac{{\sigma_{I2}}^{2}}{n_{I2}}}$$

where $\Delta_{4}$ is the uncertainty of time measurement (see Table 2); $\Delta_{5}$ is the uncertainty in the measurement of the percentage intensity (see Table 2); σ is the standard deviation from the mean of measurements for bottle A8 series and preform with 25% rPET content (see Tables S5 and S6); n is the sample size.

### Appendix B.6. Bottle Macroscopic Properties (Thickness Profile and Pressure Resistance)

Figure 2b shows the shape of the bottle being analyzed and cross-sections, where I, II, and III cross-sections were placed. In each cross-section, three measuring points are located circumferentially, in which the thickness was measured. The most likely value of absolute thickness measurement uncertainty is shown by Formula (A16). In all formulas of measurement uncertainty, the problem is to determine the extension factor “k”, but it was assumed to be equal to 1.96.

(A16) $$\Delta_{t_{ij}}=k\cdot \sqrt{{\left(\frac{\Delta_{6}}{\sqrt{3}}\right)}^{2}+\frac{{\sigma_{ij}}^{2}}{n_{ij}}}$$

where $\Delta_{t_{ij}}$ is the most likely absolute error value for thickness measurements at the i-th measuring point (see Figure 2b) of the j-th bottle for the assumed confidence level p = 0.95; k is the extension factor (for p = 0.95, k = 1.96 was assumed); $\sigma_{ij}$ is the standard deviation from the average of measurements (if the distribution of results is a normal distribution, then the standard deviation from the mean as a measure of measurement error can be taken) at the i-th measuring point of the j-th bottle; $n_{ij}$ is the number of measurement repetitions at the i-th measuring point of the j-th bottle; $\Delta_{6}$ is the measurement uncertainty of inductive sensor FH4 (Table 2).

In addition, the bottle was tested for pressure resistance. The pressure resistance test consisted of pumping water inside a bottle and uniformly increasing the pressure of that water until the bottle burst. The water pressure at which the burst occurred was a measure of the pressure resistance of the bottle. The most likely value of the measurement uncertainty in the measurement of the pressure resistance is shown by Formula (A17).

(A17) $$\Delta_{b}=k\cdot \sqrt{{\left(\frac{\Delta_{7}}{\sqrt{3}}\right)}^{2}+\frac{{\sigma_{b}}^{2}}{n_{b}}}$$

where $\Delta_{b}$ is the most likely absolute error value of the pressure resistance measure of the entire bottle for the assumed confidence level p = 0.95; $\Delta_{7}$ is the uncertainty of measurement of pressure resistance testing machines (Table 2); k is the extension factor (for p = 0.95, k = 1.96 was assumed); n is the number of repetitions of measurements, the sample size; σ is the standard deviation from the average of measurements.
