# Supplementary material for: Effect of rPET Content and Preform Heating/Cooling Conditions in the Stretch Blow Molding Process on Microcavitation and Solid-State Post-Condensation of vPET-rPET Blend: Part I—Research Methodology and Results
Source: Materials (Basel). 2024 Oct 27;17(21):5233. doi: 10.3390/ma17215233 (PMC11548011; doi:10.3390/ma17215233)
Supplement: Supplementary file 1 [file materials-17-05233-s001.zip › materials-3245021-supplementary.pdf]

Supporting Information

Effect of rPET Content and Preform Heating/Cooling Conditions in the Stretch Blow Molding Process on Microcavitation and Solid-State Post-Condensation of vPET-rPET Blend: Part I—Research Methodology and Results

Paweł Wawrzyniak[1]*, Waldemar Karaszewski[2], Artur Różański[3]

[1] Faculty of Automotive and Construction Machinery Engineering, Warsaw University of Technology, 84 Ludwika Narbutta Street, 02-524 Warsaw, Poland; * Corresponding author (pawel.wawrzyniak@pw.edu.pl)

[2] Faculty of Mechanical Engineering and Ship Technology, Gdańsk University of Technology, 11/12 Gabriela Narutowicza Street, 80-233 Gdańsk, Poland

[3] Centre of Molecular and Macromolecular Studies, Polish Academy of Sciences, 112 Sienkiewicza Street, 90-363 Łódz, Poland

S.1. Statistical analysis methodology

S.1.1. DOE for the Three-Factor, Three-Valued Plan 3^(k-p)

It should be emphasized that linear standardized two-valued plans (with the independent variables of -1, +1) and quadratic standardized three-valued plans (with the independent variables of -1, 0, +1) use very simplified calculations of the values of the main linear effects (τ, β, γ), main square effects (τ^2, β^2, γ^2), and linear interaction effects ((τβ), (τγ), (βγ)) based on the mean measurements for each series. Figure S1a shows the geometric interpretation of the main quadratic effect and the main linear effect in the case of a three-valued single-factor plan—Figure S1 also shows linear and quadratic equations and graphs approximating the measurements (based on linear multiple regression). The linear effect was calculated according to Formula (S1), and the quadratic effect according to Formula (S2). There is a relationship between the linear effect and the quadratic effect and the coefficients of the linear and quadratic equations approximating the measurements, as shown by Formulas (S3), (S4), and (S5), respectively. It should be emphasized that knowing the linear and quadratic effect makes it possible to estimate the existence of an extremum in the variability of the independent variable. And if Condition (S8) is met, then there is an extreme of the dependent variable in the range from -1 to 1 of changes in the independent variable: maximum for e.Q> 0, minimum for e.Q <0.

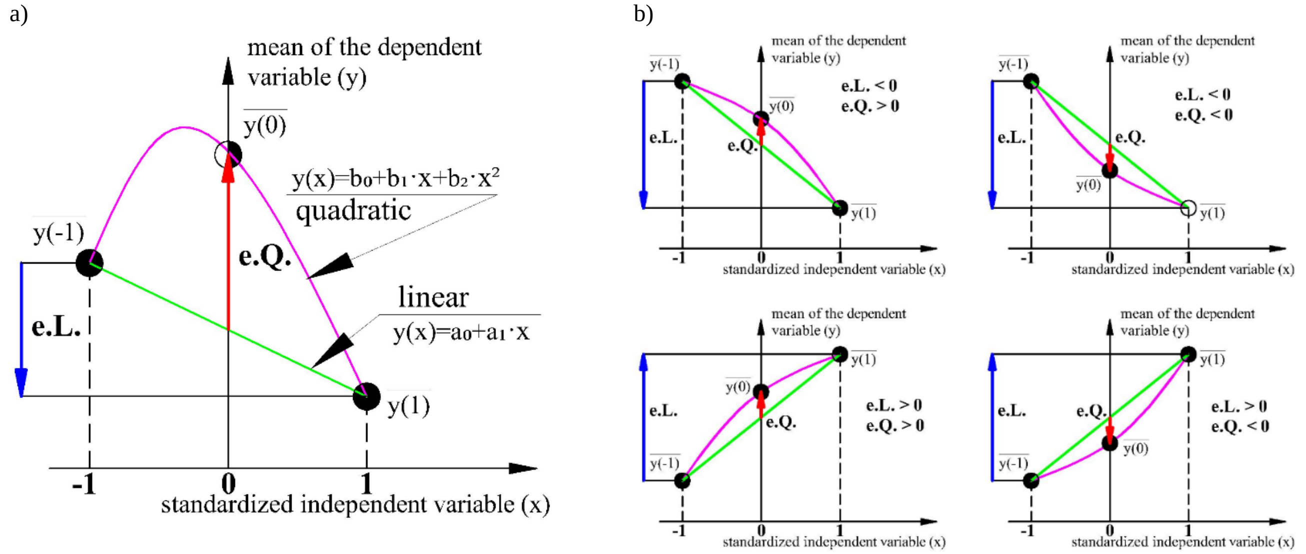

Figure S1. The main quadratic effect, where: a) geometric interpretation of the main linear effect (e.L. = τ) and the main quadratic effect (e.Q. = τ^2) for a three-valued single-factor plan with standardized values of the independent variable from -1 to 1 (i.e., -1, 0, 1), when condition (S8) is met; b) presentation of the method of interpretation of the quadratic non-linear effect in the case of the unfulfilled condition of the occurrence of an extreme (Equation (S6) is met) for a single-factor three-valued plan with standardized values of the independent variable (-1,0,1).

If Condition (S6) is met, then there is no extreme, and the trend sign of changes remains the same in terms of changes in the independent variables, but the rate of these changes is different. If Condition (S7) is met, then there is no clear evidence of the occurrence of an extreme, and the change in the sign of trend is undetermined whether it occurs at all (may exist, but should be checked for more values of the independent variable). The change in speed depends on the sign of the linear effect and the quadratic effect, as shown in Figure S1b (in the absence of an extreme). Based on Figure S1b, it follows that:

- If e.f.> 0 and e.Q.> 0, then for low values of the independent variable, the dependent variable increases rapidly, but the rate of rise decreases with the increase of the value of the independent variable. For a large value of the independent variable, the rate of change of the dependent variable is much slower, but the value of the dependent variable keeps growing.
- If e.f.> 0 and e.Q. <0, then for low values of the independent variable, the dependent variable grows very slowly, but the growth rate increases with the increase of the independent variable value. For high values of the independent variable, the growth rate of the dependent variable is very high and increases with the growth of the independent variable.
- If e.f. <0 and e.Q.> 0, then for low values of the independent variable, the dependent variable slowly decreases, but the falling speed increases with the increase of the independent variable. For a large value of the independent variable, the rate of change of the dependent variable is much greater and increases with the increase in the independent variable.
- If e.f. <0 and e.Q. <0, then for low values of the independent variable, the dependent variable decreases quickly, but the rate of this decrease slows with the increase of the independent variable. For a large value of the independent variable, the rate of change of the dependent variable is much slower, but the value of the dependent variable keeps decreasing.

Main linear effect from DOE analysis	$e.L. = \overline{y(1)} - \overline{y(-1)}$	(S1)
Main quadratic effect from DOE analysis	$e.Q. = \overline{y(0)} - \frac{\overline{y(1)} + \overline{y(-1)}}{2}$	(S2)
Relationships between the effects of DOE analysis and the coefficients of linear regression equations	$a_0 = \frac{\overline{y(1)} + \overline{y(-1)}}{2}$	(S3)
	$a_1 = b_1 = \frac{e.L.}{2} = \frac{\overline{y(1)} - \overline{y(-1)}}{2}$	(S4)
	$b_2 = -e.Q.$	(S5)
If this condition is met, then there is not an extreme of the dependent variable in the range from -1 to 1 of changes in the independent variable. The change in the dependent variable is non-linear, but without changing the sign of the change trend.	$\lvert e.Q\rvert < \frac{\lvert e.L\rvert}{4}$ $\lvert b_2\rvert < \frac{\lvert b_1\rvert}{2}$	(S6)
If this condition is met, then there is no clear evidence an extreme of the dependent variable in the range from -1 to 1 of changes in the independent variable. The change in the dependent variable is non-linear, but there is no clear evidence of a change in the sign of the trend of changes in the dependent variable.	$\frac{\lvert e.L\rvert}{4} < \lvert e.Q\rvert < \frac{\lvert e.L\rvert}{2}$ $\frac{\lvert b_1\rvert}{2} < \lvert b_2\rvert < \lvert b_1\rvert$	(S7)
If this condition is met, then there is definitely an extreme of the dependent variable in the range from -1 to 1 of changes in the independent variable:<br>maximum for e.Q> 0<br>minimum for e.Q <0	$\lvert e.Q\rvert > \frac{\lvert e.L\rvert}{2}$ $\lvert b_2\rvert > \lvert b_1\rvert$	(S8)

The geometric interpretation of the individual main quadratic effect and the individual main linear effect for a three-valued three-factor plan is analogous to that for a three-valued single-factor plan (Figures S1a and S1b). Table S1 shows the design of the three-way, three-valued experiment (Table 1[(a)]) along with the method of calculating the main linear effects (τ, β, γ), main quadratic effects (τ^2, β^2, γ^2), and linear two-way interaction effects ((τβ), (τγ), (βγ)), with Formulas (S9), (S10), (S11), (S12), (S13), (S14), (S15), (S16), (S17), respectively.

Table S1. Plan of a three-way, three-valued experiment (Figure S2a) with the method of calculating the main linear effects (τ, β, γ), main quadratic effects (τ^2, β^2, γ^2), and linear two-way interaction effects ((τβ), (τγ), (βγ))

NoE	Main factors			Interaction factors			Mean response
	A	B	C	A*B	A*C	B*C	$\tilde{y}$
1	-1	-1	-1	1	1	1	$\overline{y_1}$
2	-1	-1	1	1	-1	-1	$\overline{y_2}$
3	-1	0	0	0	0	0	$\overline{y_3}$
4	-1	1	-1	-1	1	-1	$\overline{y_4}$
5	-1	1	1	-1	-1	1	$\overline{y_5}$
6	0	-1	0	0	0	0	$\overline{y_6}$
7	0	0	-1	0	0	0	$\overline{y_7}$
8	0	0	0	0	0	0	$\overline{y_8}$

9	0	0	1	0	0	0	$\bar{y}_9$
10	0	1	0	0	0	0	$\overline{y_{10}}$
11	1	-1	-1	-1	-1	1	$\overline{y_{11}}$
12	1	-1	1	-1	1	-1	$\overline{y_{12}}$
13	1	0	0	0	0	0	$\overline{y_{13}}$
14	1	1	-1	1	-1	-1	$\overline{y_{14}}$
15	1	1	1	1	1	1	$\overline{y_{15}}$
$\tau = \frac{\overline{y_{11}} + \overline{y_{12}} + \overline{y_{13}} + \overline{y_{14}} + \overline{y_{15}}}{5} - \frac{\overline{y_1} + \overline{y_2} + \overline{y_3} + \overline{y_4} + \overline{y_5}}{5}$							(S9)
$\beta = \frac{\overline{y_4} + \overline{y_5} + \overline{y_{10}} + \overline{y_{14}} + \overline{y_{15}}}{5} - \frac{\overline{y_1} + \overline{y_2} + \overline{y_6} + \overline{y_{11}} + \overline{y_{12}}}{5}$							(S10)
$\gamma = \frac{\overline{y_2} + \overline{y_5} + \overline{y_9} + \overline{y_{12}} + \overline{y_{15}}}{5} - \frac{\overline{y_1} + \overline{y_4} + \overline{y_7} + \overline{y_{11}} + \overline{y_{14}}}{5}$							(S11)
$\tau^2 = \frac{\overline{y_6} + \overline{y_7} + \overline{y_8} + \overline{y_9} + \overline{y_{10}}}{5} - \frac{1}{2}\left(\frac{\overline{y_{11}} + \overline{y_{12}} + \overline{y_{13}} + \overline{y_{14}} + \overline{y_{15}}}{5} + \frac{\overline{y_1} + \overline{y_2} + \overline{y_3} + \overline{y_4} + \overline{y_5}}{5}\right)$							(S12)
$\beta^2 = \frac{\overline{y_3} + \overline{y_7} + \overline{y_8} + \overline{y_9} + \overline{y_{13}}}{5} - \frac{1}{2}\left(\frac{\overline{y_4} + \overline{y_5} + \overline{y_{10}} + \overline{y_{14}} + \overline{y_{15}}}{5} + \frac{\overline{y_1} + \overline{y_2} + \overline{y_6} + \overline{y_{11}} + \overline{y_{12}}}{5}\right)$							(S13)
$\gamma^2 = \frac{\overline{y_3} + \overline{y_6} + \overline{y_8} + \overline{y_{10}} + \overline{y_{13}}}{5} - \frac{1}{2}\left(\frac{\overline{y_2} + \overline{y_5} + \overline{y_9} + \overline{y_{12}} + \overline{y_{15}}}{5} + \frac{\overline{y_1} + \overline{y_4} + \overline{y_7} + \overline{y_{11}} + \overline{y_{14}}}{5}\right)$							(S14)
$(\tau\beta) = \frac{\overline{y_1} + \overline{y_2} + \overline{y_{14}} + \overline{y_{15}}}{4} - \frac{\overline{y_4} + \overline{y_5} + \overline{y_{11}} + \overline{y_{12}}}{4}$							(S15)
$(\tau\gamma) = \frac{\overline{y_1} + \overline{y_4} + \overline{y_{12}} + \overline{y_{15}}}{4} - \frac{\overline{y_2} + \overline{y_5} + \overline{y_{11}} + \overline{y_{14}}}{4}$							(S16)
$(\beta\gamma) = \frac{\overline{y_1} + \overline{y_5} + \overline{y_{11}} + \overline{y_{15}}}{4} - \frac{\overline{y_2} + \overline{y_4} + \overline{y_{12}} + \overline{y_{14}}}{4}$							(S17)

A complete three-factor, three-valued plan consists of 27 series, as represented graphically in Figure S2a, where Factors A, B, and C have three levels each. However, this article uses an incomplete three-factor plan (CCF) consisting of 15 measurement series (as represented graphically in Table 1[(a)]). Figure S2b shows the geometric interpretation of the formulas for calculating the three linear main effects (Formulas (S9), (S10), (S11)), the three quadratic main effects (Formulas (S12), (S13), (S14)), and the three linear effects of the two-way interaction (Formulas (S15), (S16), (S17)).

a) b)

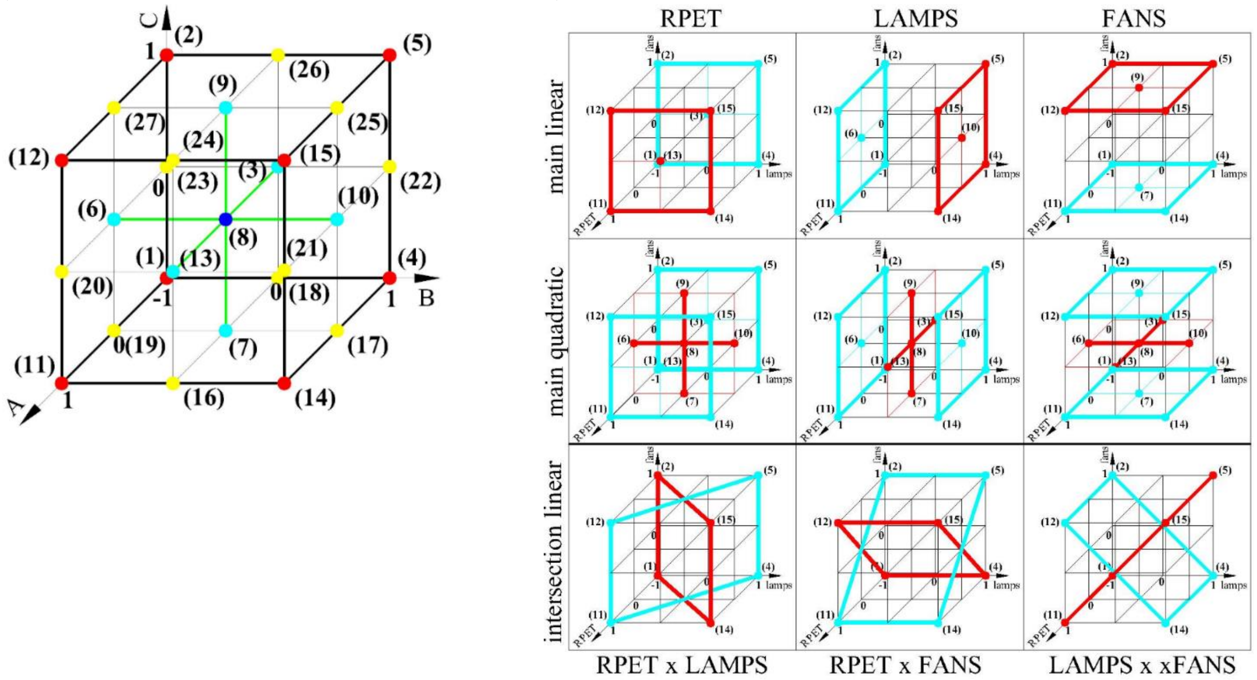

Figure S2. The complete three-factor plan consisting of 27 series of measurements, where: a) graphic representation; b) graphic representation of the groups of measurement series used in computing each linear main effect (Formulas (S9), (S10), (S11)), each quadratic main effect (Formulas (S12), (S13), (S14)), and each linear two-way interaction effect (Formulas (S15), (S16), (S17)) for the analyzed the CCF design, where cyan represents negative (-) series and red represents positive (+) series occurring in Formulas (S9), (S10), (S11), (S12), (S13), (S14), (S15), (S16), (S17).

Compared to linear main effects, the geometric interpretation of linear two-way interactions depends on the value and sign of the individual effects for which the interaction is calculated. The influence of the value and sign

of the linear two-way interaction AxB on the interpretation of the effect of Factor A depending on the level of Factor B, and vice versa, is summarized in Table S2.

In Table S2, the equality (=) relationship between the effects was defined by the 5% tolerance range for the variability of standardized effects, i.e., if the 5% tolerance window for two effects overlaps, it was assumed that both effects are equal; Formula (S*18*) is given as an example for the analysis of the equality between the values of the "A" effect and the "B" effect. The analysis of the equality between the other effects is analogous.

$$(|A| = |B|) \Leftrightarrow \{[(0.975 \cdot |A| < 1.025 \cdot |B|) \wedge (0.975 \cdot |A| > 0.975 \cdot |B|)] \vee [(1.025 \cdot |A| > 0.975 \cdot |B|) \wedge (1.025 \cdot |A| < 1.025 \cdot |B|)]\} \quad \text{(S18)}$$

Table S2. Influence of the value and sign of the two-way interaction AxB on the interpretation of the Factor A effect depending on the level of Factor B, and vice versa.

No.	Effect sign			Relative absolute values of effects			Factor A (dependent variable A)					Factor B (dependent variable B)				
	A	B	AxB	|A|vs.|B|	|AxB|vs.|A|	|AxB|vs.|B|	Trend of change for a low level of Factor B: (B-)	Trend of change for a high level of Factor B: (B +)	Initial value, i.e., in A-, for low and high levels of Factor B: (B-)vs(B+)	Trends of change for low and high levels of Factor B intersect in the domain of change of Factor A	Relative absolute value of the trends for low and high levels of Factor B: |B-|vs.|B+|	Trend of change for a low level of Factor A: (A-)	Trend of change for a high level of Factor A: (A +)	Initial value, i.e., in B-, for low and high levels of Factor A: (A-)vs(A+)	Trends of change for low and high levels of Factor A intersect in the domain of change of Factor B	Relative absolute value of the trends for low and high levels of Factor A: |A-|vs.|A+|
1	0	0	0	=	=	=	(0)	(0)	=	n.a.1	n.a.2	(0)	(0)	=	n.a.1	n.a.2
2	0	0	+	=	>	>	(-)	(+)	>	YES	(=)	(-)	(+)	>	YES	(=)
3	0	0	-	=	>	>	(+)	(-)	<	YES	(=)	(+)	(-)	<	YES	(=)
4	0	+	0	<	=	<	(0)	(0)	<	n.a.1	n.a.2	(=B)	(=B)	=	n.a.1	(=)
5	0	-	0	<	=	<	(0)	(0)	>	n.a.1	n.a.2	(=B)	(=B)	=	n.a.1	(=)
6	+	0	0	>	<	=	(=A)	(=A)	=	n.a.1	(=)	(0)	(0)	<	n.a.1	n.a.2
7	-	0	0	>	<	=	(=A)	(=A)	=	n.a.1	(=)	(0)	(0)	>	n.a.1	n.a.2
8	0	+	+	<	>	=	(-)	(+)	=	(A-)	(=)	(0)	(+)	>	YES	n.a.2
9	0	+	-	<	>	=	(+)	(-)	<	(A+)	(=)	(+)	(0)	<	YES	n.a.2
10	0	-	+	<	>	=	(-)	(+)	>	(A+)	(=)	(-)	(0)	>	YES	n.a.2
11	0	-	-	<	>	=	(+)	(-)	=	(A-)	(=)	(0)	(-)	<	YES	n.a.2
12	0	+	+	<	>	>	(-)	(+)	>	YES	(=)	(-)	(+)	>	YES	<
13	0	+	-	<	>	>	(+)	(-)	<	YES	(=)	(+)	(-)	<	YES	>
14	0	-	+	<	>	>	(-)	(+)	>	YES	(=)	(-)	(+)	>	YES	>
15	0	-	-	<	>	>	(+)	(-)	<	YES	(=)	(+)	(-)	<	YES	<
16	0	+	+	<	>	<	(-)	(+)	<	NO	(=)	(+)	(+)	>	YES	<
17	0	+	-	<	>	<	(+)	(-)	<	NO	(=)	(+)	(+)	<	YES	>
18	0	-	+	<	>	<	(-)	(+)	>	NO	(=)	(-)	(-)	>	YES	>
19	0	-	-	<	>	<	(+)	(-)	>	NO	(=)	(-)	(-)	<	YES	<
20	+	0	+	>	=	>	(0)	(+)	>	YES	n.a.2	(-)	(+)	=	(B-)	(=)
21	+	0	-	>	=	>	(+)	(0)	<	YES	n.a.2	(+)	(-)	<	(B+)	(=)
22	-	0	+	>	=	>	(-)	(0)	>	YES	n.a.2	(-)	(+)	>	(B+)	(=)
23	-	0	-	>	=	>	(0)	(-)	<	YES	n.a.2	(+)	(-)	=	(B-)	(=)
24	+	0	+	>	>	>	(-)	(+)	>	YES	(<)	(-)	(+)	>	YES	(=)
25	+	0	-	>	>	>	(+)	(-)	<	YES	(>)	(+)	(-)	<	YES	(=)
26	-	0	+	>	>	>	(-)	(+)	>	YES	(>)	(-)	(+)	>	YES	(=)

27	-	0	-	>	>	>	(+)	(-)	<	YES	<	(+)	(-)	<	YES	(=)
28	+	0	+	>	<	>	(+)	(+)	>	YES	<	(-)	(+)	<	NO	(=)
29	+	0	-	>	<	>	(+)	(+)	<	YES	(>)	(+)	(-)	<	NO	(=)
30	-	0	+	>	<	>	(-)	(-)	>	YES	(>)	(-)	(+)	>	NO	(=)
31	-	0	-	>	<	>	(-)	(-)	<	YES	<	(+)	(-)	>	NO	(=)
32	+	+	0	=	<	<	(+)	(+)	<	n.a.1	(=)	(+)	(+)	<	n.a.1	(=)
33	+	-	0	=	<	<	(+)	(+)	>	n.a.1	(=)	(-)	(-)	<	n.a.1	(=)
34	-	+	0	=	<	<	(-)	(-)	<	n.a.1	(=)	(+)	(+)	>	n.a.1	(=)
35	-	-	0	=	<	<	(-)	(-)	>	n.a.1	(=)	(-)	(-)	>	n.a.1	(=)
36	+	+	0	>	<	<	(+)	(+)	<	n.a.1	(=)	(+)	(+)	<	n.a.1	(=)
37	+	-	0	>	<	<	(+)	(+)	>	n.a.1	(=)	(-)	(-)	<	n.a.1	(=)
38	-	+	0	>	<	<	(-)	(-)	<	n.a.1	(=)	(+)	(+)	>	n.a.1	(=)
39	-	-	0	>	<	<	(-)	(-)	>	n.a.1	(=)	(-)	(-)	>	n.a.1	(=)
40	+	+	0	<	<	<	(+)	(+)	<	n.a.1	(=)	(+)	(+)	<	n.a.1	(=)
41	+	-	0	<	<	<	(+)	(+)	>	n.a.1	(=)	(-)	(-)	<	n.a.1	(=)
42	-	+	0	<	<	<	(-)	(-)	<	n.a.1	(=)	(+)	(+)	>	n.a.1	(=)
43	-	-	0	<	<	<	(-)	(-)	>	n.a.1	(=)	(-)	(-)	>	n.a.1	(=)
44	-	-	-	=	=	=	(0)	(-)	=	(A-)	n.a.2	(0)	(-)	=	(B-)	n.a.2
45	-	-	+	=	=	=	(-)	(0)	>	(A+)	n.a.2	(-)	(0)	>	(B+)	n.a.2
46	-	+	-	=	=	=	(0)	(-)	<	(A+)	n.a.2	(+)	(0)	=	(B-)	n.a.2
47	-	+	+	=	=	=	(+)	(0)	=	(A-)	n.a.2	(0)	(+)	>	(B+)	n.a.2
48	+	-	-	=	=	=	(+)	(0)	=	(A-)	n.a.2	(0)	(-)	<	(B+)	n.a.2
49	+	-	+	=	=	=	(0)	(+)	>	(A+)	n.a.2	(-)	(0)	=	(B-)	n.a.2
50	+	+	-	=	=	=	(+)	(0)	<	(A+)	n.a.2	(+)	(0)	<	(B+)	n.a.2
51	+	+	+	=	=	=	(0)	(+)	=	(A-)	n.a.2	(0)	(+)	=	(B-)	n.a.2
52	-	-	-	=	>	>	(+)	(-)	<	YES	(<)	(+)	(-)	<	YES	(<)
53	-	-	+	=	>	>	(-)	(+)	>	YES	(>)	(-)	(+)	>	YES	(>)
54	-	+	-	=	>	>	(+)	(-)	<	YES	(<)	(+)	(-)	<	YES	(>)
55	-	+	+	=	>	>	(-)	(+)	>	YES	(>)	(-)	(+)	>	YES	(<)
56	+	-	-	=	>	>	(+)	(-)	<	YES	(>)	(+)	(-)	<	YES	(<)
57	+	-	+	=	>	>	(-)	(+)	>	YES	(<)	(-)	(+)	>	YES	(>)
58	+	+	-	=	>	>	(+)	(-)	<	YES	(>)	(+)	(-)	<	YES	(>)
59	+	+	+	=	>	>	(-)	(+)	>	YES	(<)	(-)	(+)	>	YES	(<)
60	-	-	-	=	<	<	(-)	(-)	>	NO	(<)	(-)	(-)	>	NO	(<)
61	-	-	+	=	<	<	(-)	(-)	>	NO	(>)	(-)	(-)	>	NO	(>)
62	-	+	-	=	<	<	(-)	(-)	<	NO	(<)	(+)	(+)	>	NO	(>)
63	-	+	+	=	<	<	(-)	(-)	<	NO	(>)	(+)	(+)	>	NO	(<)
64	+	-	-	=	<	<	(+)	(+)	>	NO	(>)	(-)	(-)	<	NO	(<)
65	+	-	+	=	<	<	(+)	(+)	>	NO	(<)	(-)	(-)	<	NO	(>)
66	+	+	-	=	<	<	(+)	(+)	<	NO	(>)	(+)	(+)	<	NO	(>)
67	+	+	+	=	<	<	(+)	(+)	<	NO	(<)	(+)	(+)	<	NO	(<)
68	-	-	-	>	=	>	(0)	(-)	<	YES	n.a.2	(+)	(-)	=	(B-)	(<)
69	-	-	+	>	=	>	(-)	(0)	>	YES	n.a.2	(-)	(+)	>	(B+)	(>)
70	-	+	-	>	=	>	(0)	(-)	<	YES	n.a.2	(+)	(-)	=	(B-)	(>)
71	-	+	+	>	=	>	(-)	(0)	>	YES	n.a.2	(-)	(+)	>	(B+)	(<)
72	+	-	-	>	=	>	(+)	(0)	<	YES	n.a.2	(+)	(-)	<	(B+)	(<)
73	+	-	+	>	=	>	(0)	(+)	>	YES	n.a.2	(-)	(+)	=	(B-)	(>)
74	+	+	-	>	=	>	(+)	(0)	<	YES	n.a.2	(+)	(-)	<	(B+)	(>)
75	+	+	+	>	=	>	(0)	(+)	>	YES	n.a.2	(-)	(+)	=	(B-)	(<)
76	-	-	-	<	=	<	(0)	(-)	>	NO	n.a.2	(-)	(-)	=	(B-)	(<)
77	-	-	+	<	=	<	(-)	(0)	>	NO	n.a.2	(-)	(-)	>	(B+)	(>)

78	-	+	-	<	=	<	(0)	(-)	<	NO	n.a.2	(+)	(+)	=	(B-)	(>)
79	-	+	+	<	=	<	(-)	(0)	<	NO	n.a.2	(+)	(+)	>	(B+)	(<)
80	+	-	-	<	=	<	(+)	(0)	>	NO	n.a.2	(-)	(-)	<	(B+)	(<)
81	+	-	+	<	=	<	(0)	(+)	>	NO	n.a.2	(-)	(-)	=	(B-)	(>)
82	+	+	-	<	=	<	(+)	(0)	<	NO	n.a.2	(+)	(+)	<	(B+)	(>)
83	+	+	+	<	=	<	(0)	(+)	<	NO	n.a.2	(+)	(+)	=	(B-)	(<)
84	-	-	-	<	<	=	(+)	(-)	=	(A-)	(<)	(0)	(-)	<	YES	n.a.2
85	-	-	+	<	<	=	(-)	(+)	>	(A+)	(>)	(-)	(0)	>	YES	n.a.2
86	-	+	-	<	<	=	(+)	(-)	<	(A+)	(<)	(+)	(0)	<	YES	n.a.2
87	-	+	+	<	<	=	(-)	(+)	=	(A-)	(>)	(0)	(+)	>	YES	n.a.2
88	+	-	-	<	<	=	(+)	(-)	=	(A-)	(>)	(0)	(-)	<	YES	n.a.2
89	+	-	+	<	<	=	(-)	(+)	>	(A+)	(<)	(-)	(0)	>	YES	n.a.2
90	+	+	-	<	<	=	(+)	(-)	<	(A+)	(>)	(+)	(0)	<	YES	n.a.2
91	+	+	+	<	<	=	(-)	(+)	=	(A-)	(<)	(0)	(+)	>	YES	n.a.2
92	-	-	-	<	>	=	(-)	(-)	=	(A-)	(<)	(0)	(-)	<	YES	n.a.2
93	-	-	+	<	>	=	(-)	(+)	>	(A+)	(>)	(-)	(0)	>	YES	n.a.2
94	-	+	-	<	>	=	(+)	(-)	<	(A+)	(<)	(+)	(0)	<	YES	n.a.2
95	-	+	+	<	>	=	(-)	(+)	=	(A-)	(>)	(0)	(+)	>	YES	n.a.2
96	+	-	-	<	>	=	(+)	(-)	=	(A-)	(>)	(0)	(-)	<	YES	n.a.2
97	+	-	+	<	>	=	(-)	(+)	>	(A+)	(<)	(-)	(0)	>	YES	n.a.2
98	+	+	-	<	>	=	(+)	(-)	<	(A+)	(>)	(+)	(0)	<	YES	n.a.2
99	+	+	+	<	>	=	(-)	(+)	=	(A-)	(<)	(0)	(+)	>	YES	n.a.2
100	-	-	-	>	<	=	(-)	(-)	=	(A-)	(<)	(0)	(-)	>	NO	n.a.2
101	-	-	+	>	<	=	(-)	(-)	>	(A+)	(>)	(-)	(0)	>	NO	n.a.2
102	-	+	-	>	<	=	(-)	(-)	<	(A+)	(<)	(+)	(0)	>	NO	n.a.2
103	-	+	+	>	<	=	(-)	(-)	=	(A-)	(>)	(0)	(+)	>	NO	n.a.2
104	+	-	-	>	<	=	(+)	(+)	=	(A-)	(>)	(0)	(-)	<	NO	n.a.2
105	+	-	+	>	<	=	(+)	(+)	>	(A+)	(<)	(-)	(0)	<	NO	n.a.2
106	+	+	-	>	<	=	(+)	(+)	<	(A+)	(>)	(+)	(0)	<	NO	n.a.2
107	+	+	+	>	<	=	(+)	(+)	=	(A-)	(<)	(0)	(+)	<	NO	n.a.2
108	-	-	-	>	>	>	(+)	(-)	<	YES	(<)	(+)	(-)	<	YES	(<)
109	-	-	+	>	>	>	(-)	(+)	>	YES	(>)	(-)	(+)	>	YES	(>)
110	-	+	-	>	>	>	(+)	(-)	<	YES	(<)	(+)	(-)	<	YES	(>)
111	-	+	+	>	>	>	(-)	(+)	>	YES	(>)	(-)	(+)	>	YES	(<)
112	+	-	-	>	>	>	(+)	(-)	<	YES	(>)	(+)	(-)	<	YES	(<)
113	+	-	+	>	>	>	(-)	(+)	>	YES	(<)	(-)	(+)	>	YES	(>)
114	+	+	-	>	>	>	(+)	(-)	<	YES	(>)	(+)	(-)	<	YES	(>)
115	+	+	+	>	>	>	(-)	(+)	>	YES	(<)	(-)	(+)	>	YES	(<)
116	-	-	-	>	<	<	(-)	(-)	>	NO	(<)	(-)	(-)	>	NO	(<)
117	-	-	+	>	<	<	(-)	(-)	>	NO	(>)	(-)	(-)	>	NO	(>)
118	-	+	-	>	<	<	(-)	(-)	<	NO	(<)	(+)	(+)	>	NO	(>)
119	-	+	+	>	<	<	(-)	(-)	<	NO	(>)	(+)	(+)	>	NO	(<)
120	+	-	-	>	<	<	(+)	(+)	>	NO	(>)	(-)	(-)	<	NO	(<)
121	+	-	+	>	<	<	(+)	(+)	>	NO	(<)	(-)	(-)	<	NO	(>)
122	+	+	-	>	<	<	(+)	(+)	<	NO	(>)	(+)	(+)	<	NO	(>)
123	+	+	+	>	<	<	(+)	(+)	<	NO	(<)	(+)	(+)	<	NO	(<)
124	-	-	-	>	<	>	(-)	(-)	<	YES	(<)	(+)	(-)	>	NO	(<)
125	-	-	+	>	<	>	(-)	(-)	>	YES	(>)	(-)	(+)	>	NO	(>)
126	-	+	-	>	<	>	(-)	(-)	<	YES	(<)	(+)	(-)	>	NO	(>)
127	-	+	+	>	<	>	(-)	(-)	>	YES	(>)	(-)	(+)	>	NO	(<)
128	+	-	-	>	<	>	(+)	(+)	<	YES	(>)	(+)	(-)	<	NO	(<)
129	+	-	+	>	<	>	(+)	(+)	>	YES	(<)	(-)	(+)	<	NO	(>)

130	+	+	-	>	<	>	(+)	(+)	<	YES	(>)	(+)	(-)	<	NO	(>)
131	+	+	+	>	<	>	(+)	(+)	>	YES	(<)	(-)	(+)	<	NO	(<)
132	-	-	-	<	<	<	(-)	(-)	>	NO	(<)	(-)	(-)	>	NO	(<)
133	-	-	+	<	<	<	(-)	(-)	>	NO	(>)	(-)	(-)	>	NO	(>)
134	-	+	-	<	<	<	(-)	(-)	<	NO	(<)	(+)	(+)	>	NO	(>)
135	-	+	+	<	<	<	(-)	(-)	<	NO	(>)	(+)	(+)	>	NO	(<)
136	+	-	-	<	<	<	(+)	(+)	>	NO	(>)	(-)	(-)	<	NO	(<)
137	+	-	+	<	<	<	(+)	(+)	>	NO	(<)	(-)	(-)	<	NO	(>)
138	+	+	-	<	<	<	(+)	(+)	<	NO	(>)	(+)	(+)	<	NO	(>)
139	+	+	+	<	<	<	(+)	(+)	<	NO	(<)	(+)	(+)	<	NO	(<)
140	-	-	-	<	>	>	(+)	(-)	<	YES	(<)	(+)	(-)	<	YES	(<)
141	-	-	+	<	>	>	(-)	(+)	>	YES	(>)	(-)	(+)	>	YES	(>)
142	-	+	-	<	>	>	(+)	(-)	<	YES	(<)	(+)	(-)	<	YES	(>)
143	-	+	+	<	>	>	(-)	(+)	>	YES	(>)	(-)	(+)	>	YES	(<)
144	+	-	-	<	>	>	(+)	(-)	<	YES	(>)	(+)	(-)	<	YES	(<)
145	+	-	+	<	>	>	(-)	(+)	>	YES	(<)	(-)	(+)	>	YES	(>)
146	+	+	-	<	>	>	(+)	(-)	<	YES	(>)	(+)	(-)	<	YES	(>)
147	+	+	+	<	>	>	(-)	(+)	>	YES	(<)	(-)	(+)	>	YES	(<)
148	-	-	-	<	>	<	(+)	(-)	>	NO	(<)	(-)	(-)	<	YES	(<)
149	-	-	+	<	>	<	(-)	(+)	>	NO	(>)	(-)	(-)	>	YES	(>)
150	-	+	-	<	>	<	(+)	(-)	<	NO	(<)	(+)	(+)	<	YES	(>)
151	-	+	+	<	>	<	(-)	(+)	<	NO	(>)	(+)	(+)	>	YES	(<)
152	+	-	-	<	>	<	(+)	(-)	>	NO	(>)	(-)	(-)	<	YES	(<)
153	+	-	+	<	>	<	(-)	(+)	>	NO	(<)	(-)	(-)	>	YES	(>)
154	+	+	-	<	>	<	(+)	(-)	<	NO	(>)	(+)	(+)	<	YES	(>)
155	+	+	+	<	>	<	(+)	(-)	<	NO	(<)	(+)	(+)	>	YES	(<)

Where
(n.a.1) means not applicable because there is no cross-effect
(n.a.2) means not applicable because there is at least one zero trend
(=A) means in line with the mean trend of the A series
(=B) means in line with the mean trend of the B series
(0) is a zero trend of change
(+) is a positive trend of change
(-) is a negative trend of change
(>) is a faster trend of change
(<) is a slower trend of change
YES means trends of changes for low and high levels of the second factor intersect in the domain of change of the first factor
NO means trends of changes for low and high levels of the second factor do not intersect in the domain of change of the first factor
(A-) means trends of changes for low and high levels of Factor B intersect at the point of low level of Factor A (A-)
(A+) means trends of changes for low and high levels of Factor B intersect at the point of high level of Factor A (A-)
(B-) means trends of changes for low and high levels of Factor A intersect at the point of low level of Factor B (B-)
(B+) means trends of changes for low and high levels of Factor A intersect at the point of high level of Factor B (B-)

Examples of the interpretation of the results of the linear DOE analysis for the two-factor design (linear main effects and linear interaction effects) for combination Nos. 107 and 37 from Table ***S2*** are presented in Figures S3a and S3b, respectively. For the example shown in Figure S3a, the conclusions are as follows:

- The two-way interaction effect (AxB) is statistically significant and positive; the absolute value is lower than the absolute value of the main effect of Factor A, and the absolute value of the two-way interaction effect (AxB) is equal to the absolute value of the main effect of Factor B. That is, the values resulting from the main effect for Factor (A) are not the same for the entire range of variability of Factor (B)—the main effect of Factor A should be interpreted separately for low and high levels of Factor B. The same is true for the main effect of Factor (B) with respect to the entire range of variability of Factor (A).
- The main effect of Factor A is statistically significant and positive (as for the mean value of Factor B, the higher the value of Factor A, the greater the value of the dependent variable); however, there is a two-way interaction between Factor A and Factor B: for both low and high levels of Factor B, the higher the value of Factor A, the greater the value of the dependent variable. However, for a high value of Factor B, the rate

of changes of the dependent variable under the influence of Factor A change is greater than for a low value of Factor B.

- The main effect of Factor B is statistically significant and positive (as for the mean value of Factor A, the higher the value of Factor B, the greater the value of the dependent variable); however, there is a two-way interaction between Factor A and Factor B: for a low level of Factor A, the increase in Factor B has no effect on the change of the dependent variable, while for a high value of Factor A, an increase in the value of Factor B causes an increase in the value of the dependent variable. The cross-effect occurs outside the range of Factor B variability, so in the entire range of Factor B variability, for a low level of Factor A, the values of the dependent variable are lower than for a high level of Factor A.

For the example shown in Figure S3b, the conclusions are as follows:

- The two-way interaction effect (AxB) is statistically insignificant. This means that the values resulting from the main effect for Factor (A) are the same for the entire range of Factor (B) variation. The same is true of the main effect for Factor (B) with respect to the entire range of Factor (A) variability.
- The main effect of Factor A is statistically significant and positive (as to the mean value of Factor B, the higher the value of Factor A, the greater the value of the dependent variable), and its absolute value is greater than the absolute value of the main effect B, so in the absence of a cross-effect, in across the range of Factor A variability, for a low level of Factor B, the values of the dependent variable are higher than for a high level of Factor B.
- The main effect of Factor B is statistically significant and negative (as for the mean value of Factor A, the higher the value of Factor B, the lower the value of the dependent variable), and its absolute value is smaller than the absolute value of the main effect A, so in the absence of a cross-effect, in across the range of Factor B variability, for a low level of Factor A, the values of the dependent variable are lower than for a high level of Factor A.

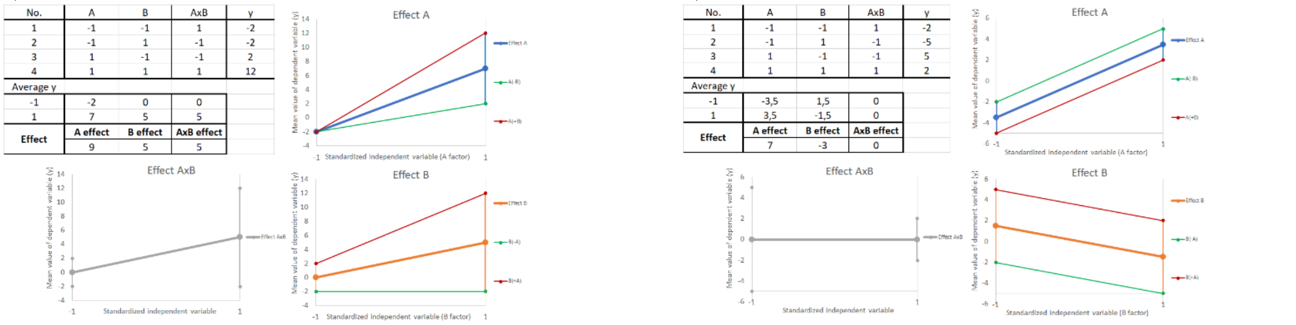

a)

No.	A	B	AxB	y
1	-1	-1	1	-2
2	-1	1	-1	-2
3	1	-1	-1	2
4	1	1	1	12
Average y				
-1	-2	0	0	
1	7	5	5	
Effect	A effect	B effect	AxB effect	
	9	5	5	

b)

No.	A	B	AxB	y
1	-1	-1	1	-2
2	-1	1	-1	-5
3	1	-1	-1	5
4	1	1	1	2
Average y				
-1	-3,5	1,5	0	
1	3,5	-1,5	0	
Effect	A effect	B effect	AxB effect	
	7	-3	0	

Figure S3. Example of DOE analysis results interpretation for a two-factor plan for: a) combination No. 107 from Table **S2**; b) combination No. 37 from Table **S2**.

S.1.2. ANOVA for the Three-Factor Plan 3^(k-p)

The statistical analysis should check whether the constructed model significantly reflects the influence of the independent variables on the dependent variables. For this, an ANOVA is required, which tests the null hypothesis that Factor A has no effects on the response at an effect level τ. The F-test statistic for each effect (Formulas (S9)–(S17)) is given by Formulas (*S88*)–(*S96*), and if this F statistic is higher than the critical value of F statistic for the given plan and type I error α (Formula (*S97*)), then this means that the given effect is statistically significant with the probability of a type I error α. ANOVA tests the significance of differences between multiple sample means from multiple populations (groups). The results of observations in these groups are dependent on one or more factors acting simultaneously. They are called categorical predictors, grouping variables, or independent variables. The variables that are measured are called dependent variables.

If only one factor influences the examined (dependent) variable, it is called a one-factor model. When more factors have an impact on the dependent variable, then it is a multifactorial model. It is worth remembering that ANOVA can be performed for both independent and dependent (repeated measurement) samples. Table S3 summarizes the formulas needed to perform ANOVA for an incomplete three-valued, three-factor design shown in Table 1[(a)]. In order to calculate dependencies (*S67*)–(*S97*) shown in Table S3, auxiliary quantities were introduced as shown in Equations (*S21*)–(*S66*).

Table S3. Summary of the formulas needed to perform ANOVA for an incomplete 3-factor, 3-valued plan, including three linear main effects, three quadratic main effects, and three linear effects of two-way interaction

Factors A, B, C			Factor Levels	Degree of Freedom (df)	Sum of Squares	Mean Square (variance)	F-statistics	F critical
A	Effect	Linear Main	A: p	$df_A = 1$	Equation (*S67*)	Equation (S78)	Equation (S88)	Equation (S97)
B			B: q	$df_B = 1$	Equation (S68)	Equation (S79)	Equation (S89)	
C			C: r	$df_C = 1$	Equation (S69)	Equation (S80)	Equation (S90)	
A^2		Quadratic Main	-	$df_{A^2} = 1$	Equation (S70)	Equation (S81)	Equation (S91)	
B^2				$df_{B^2} = 1$	Equation (S71)	Equation (S82)	Equation (S92)	
C^2				$df_{C^2} = 1$	Equation (S72)	Equation (S83)	Equation (S93)	
A*B		Linear Interacion		$df_{AB} = 1$	Equation (S73)	Equation (S84)	Equation (S94)	
B*C				$df_{AC} = 1$	Equation (S74)	Equation (S85)	Equation (S95)	
A*C				$df_{BC} = 1$	Equation (S75)	Equation (S86)	Equation (S96)	
Error				Equation (S20)	Equation (S76)	Equation (S87)	-	-
Total				Equation (*S19*)	Equation (S77)	-	-	-

$df_{total} = \sum_{i=1}^{3}\sum_{j=1}^{3}\sum_{k=1}^{3} N_{ijk} - 1$	(S19)
$df_{error} = df_{total} - (df_A + df_B + df_C + df_{A^2} + df_{B^2} + df_{C^2} + df_{AB} + df_{BC} + df_{BC} + df_{ABC})$	(S20)
Positive mean and positive group size for the linear RPET (A) main effect $\bar{y}_{+A} = \frac{\overline{y_{11}} + \overline{y_{12}} + \overline{y_{13}} + \overline{y_{14}} + \overline{y_{15}}}{5};\ N_{+A} = N_{11} + N_{12} + N_{13} + N_{14} + N_{15}$	(S21)
Negative mean and negative group size for the linear RPET (A) main effect $\bar{y}_{-A} = \frac{\overline{y_1} + \overline{y_2} + \overline{y_3} + \overline{y_4} + \overline{y_5}}{5};\ N_{-A} = N_1 + N_2 + N_3 + N_4 + N_5$	(S22)
Mean for the linear main effect of the RPET Factor (A) $\bar{y}_A = \frac{\bar{y}_{+A} + \bar{y}_{-A}}{2}$	(S23)
Positive mean and positive group size for the linear LAMPS (B) main effect $\bar{y}_{+B} = \frac{\overline{y_4} + \overline{y_5} + \overline{y_{10}} + \overline{y_{14}} + \overline{y_{15}}}{5};\ N_{+B} = N_4 + N_5 + N_{10} + N_{14} + N_{15}$	(S24)
Negative mean and negative group size for the linear LAMPS (B) main effect $\bar{y}_{-B} = \frac{\overline{y_1} + \overline{y_2} + \overline{y_6} + \overline{y_{11}} + \overline{y_{12}}}{5};\ N_{-B} = N_1 + N_2 + N_6 + N_{11} + N_{12}$	(S25)
Mean for the linear main effect of the LAMPS factor (B) $\bar{y}_B = \frac{\bar{y}_{+B} + \bar{y}_{-B}}{2}$	(S26)
Positive mean and positive group size for the linear FANS (C) main effect $\bar{y}_{+C} = \frac{\overline{y_2} + \overline{y_5} + \overline{y_9} + \overline{y_{12}} + \overline{y_{15}}}{5};\ N_{+C} = N_2 + N_5 + N_9 + N_{12} + N_{15}$	(S27)
Negative mean and negative group size for the linear FANS (C) main effect $\bar{y}_{-C} = \frac{\overline{y_1} + \overline{y_4} + \overline{y_7} + \overline{y_{11}} + \overline{y_{14}}}{5};\ N_{-C} = N_1 + N_4 + N_7 + N_{11} + N_{14}$	(S28)
Mean for the linear main effect of the FANS Factor (C) $\bar{y}_C = \frac{\bar{y}_{+C} + \bar{y}_{-C}}{2}$	(S29)
Positive mean and positive group size for the quadratic RPET (A^2) main effect $\bar{y}_{+A^2} = \frac{\overline{y_6} + \overline{y_7} + \overline{y_8} + \overline{y_9} + \overline{y_{10}}}{5};\ N_{+A^2} = N_6 + N_7 + N_8 + N_9 + N_{10}$	(S30)
Negative mean and negative group size for the quadratic RPET (A^2) main effect $\bar{y}_{-A^2} = \frac{1}{2}\left(\frac{\overline{y_{11}} + \overline{y_{12}} + \overline{y_{13}} + \overline{y_{14}} + \overline{y_{15}}}{5} + \frac{\overline{y_1} + \overline{y_2} + \overline{y_3} + \overline{y_4} + \overline{y_5}}{5}\right)$ $N_{-A^2} = N_{11} + N_{12} + N_{13} + N_{14} + N_{15} + N_1 + N_2 + N_3 + N_4 + N_5$	(S31)

Mean for the quadratic main effect of the RPET Factor (A²) $$\bar{y}_{A^2} = \frac{\bar{y}_{+A^2} + \bar{y}_{-A^2}}{2}$$	(S32)
Positive mean and positive group size for the quadratic LAMPS (B²) main effect $$\bar{y}_{+B^2} = \frac{\overline{y_3} + \overline{y_7} + \overline{y_8} + \overline{y_9} + \overline{y_{13}}}{5};\ N_{+B^2} = N_3 + N_7 + N_8 + N_9 + N_{13}$$	(S33)
Negative mean and negative group size for the quadratic LAMPS (B²) main effect $$\bar{y}_{-B^2} = \frac{1}{2}\left(\frac{\overline{y_2} + \overline{y_5} + \overline{y_9} + \overline{y_{12}} + \overline{y_{15}}}{5} + \frac{\overline{y_1} + \overline{y_4} + \overline{y_7} + \overline{y_{11}} + \overline{y_{14}}}{5}\right)$$ $$N_{-B^2} = N_2 + N_5 + N_9 + N_{12} + N_{15} + N_1 + N_4 + N_7 + N_{11} + N_{14}$$	(S34)
Mean for the quadratic main effect of the LAMPS Factor (B²) $$\bar{y}_{B^2} = \frac{\bar{y}_{+B^2} + \bar{y}_{-B^2}}{2}$$	(S35)
Positive mean and positive group size for the quadratic FANS (C²) main effect $$\bar{y}_{+C^2} = \frac{\overline{y_3} + \overline{y_6} + \overline{y_8} + \overline{y_{10}} + \overline{y_{13}}}{5};\ N_{+C^2} = N_3 + N_6 + N_8 + N_{10} + N_{13}$$	(S36)
Negative mean and negative group size for the quadratic FANS (C²) main effect $$\bar{y}_{-C^2} = \frac{1}{2}\left(\frac{\overline{y_2} + \overline{y_5} + \overline{y_9} + \overline{y_{12}} + \overline{y_{15}}}{5} + \frac{\overline{y_1} + \overline{y_4} + \overline{y_7} + \overline{y_{11}} + \overline{y_{14}}}{5}\right)$$ $$N_{-C^2} = N_2 + N_5 + N_9 + N_{12} + N_{15} + N_1 + N_4 + N_7 + N_{11} + N_{14}$$	(S37)
Mean for the quadratic main effect of the FANS Factor (C²) $$\bar{y}_{C^2} = \frac{\bar{y}_{+C^2} + \bar{y}_{-C^2}}{2}$$	(S38)
Mean and group size for the linear effect of the AB interaction with a positive value of Factor A and a positive value of Factor B $$\bar{y}_{+A+B} = \frac{\bar{y}_{14} + \bar{y}_{15}}{2};\ N_{+A+B} = N_{14} + N_{15}$$	(S39)
Mean and group size for the linear effect of the AB interaction with a positive value of Factor A and a negative value of Factor B $$\bar{y}_{+A-B} = \frac{\bar{y}_{11} + \bar{y}_{12}}{2};\ N_{+A-B} = N_{11} + N_{12}$$	(S40)
Mean and group size for the linear effect of the AB interaction with a negative value of Factor A and a positive value of Factor B $$\bar{y}_{-A+B} = \frac{\bar{y}_4 + \bar{y}_5}{2};\ N_{-A+B} = N_4 + N_5$$	(S41)
Mean and group size for the linear effect of the AB interaction with a negative value of Factor A and a negative value of Factor B $$\bar{y}_{-A-B} = \frac{\bar{y}_1 + \bar{y}_2}{2};\ N_{-A-B} = N_1 + N_2$$	(S42)
Mean for the linear effect of AB interactions from A+ $$\bar{y}_{+(A)AB} = \frac{\bar{y}_{+A+B} + \bar{y}_{+A-B}}{2}$$	(S43)
Mean for the linear effect of AB interactions from A- $$\bar{y}_{-(A)AB} = \frac{\bar{y}_{-A+B} + \bar{y}_{-A-B}}{2}$$	(S44)
Mean for the linear effect of AB interactions from B+ $$\bar{y}_{+(B)AB} = \frac{\bar{y}_{+A+B} + \bar{y}_{-A+B}}{2}$$	(S45)
Mean for the linear effect of AB interactions from B- $$\bar{y}_{-(B)AB} = \frac{\bar{y}_{+A-B} + \bar{y}_{-A-B}}{2}$$	(S46)
Mean for the linear effect of AB interactions $$\bar{y}_{AB} = \frac{\bar{y}_{+(A)AB} + \bar{y}_{-(A)AB} + \bar{y}_{+(B)AB} + \bar{y}_{-(B)AB}}{4}$$	(S47)
Mean and group size for the linear effect of the AC interaction with a positive value of Factor A and a positive value of Factor C $$\bar{y}_{+A+C} = \frac{\bar{y}_{12} + \bar{y}_{15}}{2};\ N_{+A+C} = N_{12} + N_{15}$$	(S48)
Mean and group size for the linear effect of the AC interaction with a positive value of Factor A and a negative value of Factor C $$\bar{y}_{+A-C} = \frac{\bar{y}_{11} + \bar{y}_{14}}{2};\ N_{+A-C} = N_{11} + N_{14}$$	(S49)
Mean and group size for the linear effect of the AC interaction with a negative value of Factor A and a positive value of Factor C $$\bar{y}_{-A+C} = \frac{\bar{y}_2 + \bar{y}_5}{2};\ N_{-A+C} = N_2 + N_5$$	(S50)
Mean and group size for the linear effect of the AC interaction with a negative value of Factor A and a negative value of Factor C $$\bar{y}_{-A-C} = \frac{\bar{y}_1 + \bar{y}_4}{2};\ N_{-A-C} = N_1 + N_4$$	(S51)
Mean for the linear effect of AC interactions from A+ $$\bar{y}_{+(A)AC} = \frac{\bar{y}_{+A+C} + \bar{y}_{+A-C}}{2}$$	(S52)
Mean for the linear effect of AC interactions from A-	(S53)

$\bar{y}_{-(A)AC} = \frac{\bar{y}_{-A+C} + \bar{y}_{-A-C}}{2}$	
Mean for the linear effect of AC interactions from C+<br>$\bar{y}_{+(C)AC} = \frac{\bar{y}_{+A+C} + \bar{y}_{-A+C}}{2}$	(S54)
Mean for the linear effect of AC interactions from C-<br>$\bar{y}_{-(C)AC} = \frac{\bar{y}_{+A-C} + \bar{y}_{-A-C}}{2}$	(S55)
Mean for the linear effect of AC interactions<br>$\bar{y}_{AC} = \frac{\bar{y}_{+(A)AC} + \bar{y}_{-(A)AC} + \bar{y}_{+(C)AC} + \bar{y}_{-(C)AC}}{4}$	(S56)
Mean and group size for the linear effect of the BC interaction with a positive value of Factor B and a positive value of Factor C<br>$\bar{y}_{+B+C} = \frac{\bar{y}_{11} + \bar{y}_{15}}{2};\ N_{+B+C} = N_{11} + N_{15}$	(S57)
Mean and group size for the linear effect of the BC interaction with a positive value of Factor B and a negative value of Factor C<br>$\bar{y}_{+B-C} = \frac{\bar{y}_{4} + \bar{y}_{14}}{2};\ N_{+B-C} = N_{4} + N_{14}$	(S58)
Mean and group size for the linear effect of the BC interaction with a negative value of Factor B and a positive value of Factor C<br>$\bar{y}_{-B+C} = \frac{\bar{y}_{2} + \bar{y}_{12}}{2};\ N_{-B+C} = N_{2} + N_{12}$	(S59)
Mean and group size for the linear effect of the BC interaction with a negative value of Factor B and a negative value of Factor C<br>$\bar{y}_{-B-C} = \frac{\bar{y}_{1} + \bar{y}_{5}}{2};\ N_{-B-C} = N_{1} + N_{5}$	(S60)
Mean for the linear effect of BC interactions from B+<br>$\bar{y}_{+(B)BC} = \frac{\bar{y}_{+B+C} + \bar{y}_{+B-C}}{2}$	(S61)
Mean for the linear effect of BC interactions from B-<br>$\bar{y}_{-(B)BC} = \frac{\bar{y}_{-B+C} + \bar{y}_{-B-C}}{2}$	(S62)
Mean for the linear effect of BC interactions from C+<br>$\bar{y}_{+(C)BC} = \frac{\bar{y}_{+B+C} + \bar{y}_{-B+C}}{2}$	(S63)
Mean e for the linear effect of BC interactions from C-<br>$\bar{y}_{-(C)BC} = \frac{\bar{y}_{+B-C} + \bar{y}_{-B-C}}{2}$	(S64)
Mean for the linear effect of BC interactions<br>$\bar{y}_{BC} = \frac{\bar{y}_{+(B)BC} + \bar{y}_{-(B)BC} + \bar{y}_{+(B)BC} + \bar{y}_{-(B)BC}}{4}$	(S65)
Mean for the entire plan<br>$\tilde{y} = \bar{y}_{\bullet\bullet\bullet\bullet} = \sum_{i=-1}^{1}\sum_{j=-1}^{1}\sum_{k=-1}^{1}\frac{\sum_{l=1}^{N_{ijk}} y_{ijkl}}{N_{ijk}}$	(S66)
$SS_{A.ef} = (\bar{y}_{+A} - \bar{y}_{A})^2 \cdot N_{+A} + (\bar{y}_{-A} - \bar{y}_{A})^2 \cdot N_{-A}$	(S67)
$SS_{B.ef} = (\bar{y}_{+B} - \bar{y}_{B})^2 \cdot N_{+B} + (\bar{y}_{-B} - \bar{y}_{B})^2 \cdot N_{-B}$	(S68)
$SS_{C.ef} = (\bar{y}_{+C} - \bar{y}_{C})^2 \cdot N_{+C} + (\bar{y}_{-C} - \bar{y}_{C})^2 \cdot N_{-C}$	(S69)
$S_{A^2.ef} = (\bar{y}_{+A^2} - \bar{y}_{A^2})^2 \cdot N_{+A^2} + (\bar{y}_{-A^2} - \bar{y}_{A^2})^2 \cdot N_{-A^2}$	(S70)
$SS_{B^2.ef} = (\bar{y}_{+B^2} - \bar{y}_{B^2})^2 \cdot N_{+B^2} + (\bar{y}_{-B^2} - \bar{y}_{B^2})^2 \cdot N_{-B^2}$	(S71)
$S_{C^2.ef} = (\bar{y}_{+C^2} - \bar{y}_{C^2})^2 \cdot N_{+C^2} + (\bar{y}_{-C^2} - \bar{y}_{C^2})^2 \cdot N_{-C^2}$	(S72)
$SS_{AB.ef} = \left[\bar{y}_{+A+B} - \left(\bar{y}_{+(A)AB} + \bar{y}_{+(B)AB} - \bar{y}_{AB}\right)\right]^2 \cdot N_{+A+B} + \left[\bar{y}_{+A-B} - \left(\bar{y}_{+(A)AB} + \bar{y}_{-(B)AB} - \bar{y}_{AB}\right)\right]^2 \cdot N_{+A-B} +$<br>$+\left[\bar{y}_{-A+B} - \left(\bar{y}_{-(A)AB} + \bar{y}_{+(B)AB} - \bar{y}_{AB}\right)\right]^2 \cdot N_{-A+B} + \left[\bar{y}_{-A-B} - \left(\bar{y}_{-(A)AB} + \bar{y}_{+(B)AB} - \bar{y}_{AB}\right)\right]^2 \cdot N_{-A-B}$	(S73)
$SS_{AC.ef} = \left[\bar{y}_{+A+C} - \left(\bar{y}_{+(A)AC} + \bar{y}_{+(C)AC} - \bar{y}_{AC}\right)\right]^2 \cdot N_{+A+C} + \left[\bar{y}_{+A-C} - \left(\bar{y}_{+(A)AC} + \bar{y}_{-(C)AC} - \bar{y}_{AC}\right)\right]^2 \cdot N_{+A-C} +$<br>$+\left[\bar{y}_{-A+C} - \left(\bar{y}_{-(A)AC} + \bar{y}_{+(C)AC} - \bar{y}_{AC}\right)\right]^2 \cdot N_{-A+C} + \left[\bar{y}_{-A-C} - \left(\bar{y}_{-(A)AC} + \bar{y}_{-(C)AC} - \bar{y}_{AC}\right)\right]^2 \cdot N_{-A-C}$	(S74)
$SS_{BC.ef} = \left[\bar{y}_{+B+C} - \left(\bar{y}_{+(B)BC} + \bar{y}_{+(C)BC} - \bar{y}_{BC}\right)\right]^2 \cdot N_{+B+C} + \left[\bar{y}_{+B-C} - \left(\bar{y}_{+(B)BC} + \bar{y}_{-(C)BC} - \bar{y}_{BC}\right)\right]^2 \cdot N_{+B-C} +$<br>$+\left[\bar{y}_{-B+C} - \left(\bar{y}_{-(B)BC} + \bar{y}_{+(C)BC} - \bar{y}_{BC}\right)\right]^2 \cdot N_{-B+C} + \left[\bar{y}_{-B-C} - \left(\bar{y}_{-(B)BC} + \bar{y}_{-(C)BC} - \bar{y}_{BC}\right)\right]^2 \cdot N_{-B-C}$	(S75)
$SS_{error} = \sum_{i=-1}^{1}\sum_{j=-1}^{1}\sum_{k=1}^{1}\sum_{l=1}^{N_{ijk}}\left(y_{ijkl} - \bar{y}_{ijk\bullet}\right)^2 =$<br>$= SS_{total} - (SS_{A.ef} + SS_{B.ef} + SS_{C.ef} + SS_{A^2.ef} + SS_{B^2.ef} + SS_{C^2.ef} + SS_{AB.ef} + SS_{BC.ef} + SS_{AC.ef})$	(S76)

$SS_{total} = SS_{A.ef} + SS_{B.ef} + SS_{C.ef} + SS_{A^2.ef} + SS_{B^2.ef} + SS_{C^2.ef} + SS_{AB.ef} + SS_{BC.ef} + SS_{AC.ef} + SS_{error} = = \sum_{l=1}^{N_{ijk}} \sum_{i=-1}^{1} \sum_{j=-1}^{1} \sum_{k=-1}^{1} (y_{ijkl} - \tilde{y})^2$	(S77)
$\mathrm{MS_{A.ef}} = (\hat{s}_{1.A})^2 = \frac{\mathrm{SS_{A.ef}}}{\mathrm{df_A}}$	(S78)
$MS_{B.ef} = (\hat{s}_{1.B})^2 = \frac{SS_{B.ef}}{df_B}$	(S79)
$\mathrm{MS_{C.ef}} = (\hat{s}_{1.C})^2 = \frac{\mathrm{SS_{C.ef}}}{\mathrm{df_C}}$	(S80)
$\mathrm{MS_{A^2.ef}} = (\hat{s}_{1.A^2})^2 = \frac{\mathrm{SS_{A^2.ef}}}{\mathrm{df_{A^2}}}$	(S81)
$MS_{B^2.ef} = (\hat{s}_{1.B^2})^2 = \frac{SS_{B^2.ef}}{df_{B^2}}$	(S82)
$MS_{C^2.ef} = (\hat{s}_{1.C^2})^2 = \frac{SS_{C^2.ef}}{df_{C^2}}$	(S83)
$\mathrm{MS_{AB.ef}} = (\hat{s}_{1.AB})^2 = \frac{\mathrm{SS_{AB.ef}}}{\mathrm{df_{AB}}}$	(S84)
$\mathrm{MS_{BC.ef}} = (\hat{s}_{1.BC})^2 = \frac{\mathrm{SS_{BC.ef}}}{\mathrm{df_{BC}}}$	(S85)
$MS_{AC.ef} = (\hat{s}_{1.AC})^2 = \frac{SS_{AC.ef}}{df_{AC}}$	(S86)
$\mathrm{MS_{error}} = (\hat{s}_2)^2 = \frac{\mathrm{SS_{error}}}{\mathrm{df_{error}}}$	(S87)
$\mathrm{F_A} = \frac{(\hat{s}_{1.A})^2}{(\hat{s}_2)^2}$	(S88)
$F_B = \frac{(\hat{s}_{1.B})^2}{(\hat{s}_2)^2}$	(S89)
$F_C = \frac{(\hat{s}_{1.C})^2}{(\hat{s}_2)^2}$	(S90)
$\mathrm{F_{A^2}} = \frac{(\hat{s}_{1.A^2})^2}{(\hat{s}_2)^2}$	(S91)
$F_{B^2} = \frac{(\hat{s}_{1.B^2})^2}{(\hat{s}_2)^2}$	(S92)
$F_{C^2} = \frac{(\hat{s}_{1.C^2})^2}{(\hat{s}_2)^2}$	(S93)
$\mathrm{F_{AB}} = \frac{(\hat{s}_{1.AB})^2}{(\hat{s}_2)^2}$	(S94)
$\mathrm{F_{BC}} = \frac{(\hat{s}_{1.BC})^2}{(\hat{s}_2)^2}$	(S95)
$F_{AC} = \frac{(\hat{s}_{1.AC})^2}{(\hat{s}_2)^2}$	(S96)
$F_{cr.effect} = F(\alpha, \vartheta_1 = df_{effect}, \vartheta_2 = df_{error})$	(S97)

S.1.3. ANOVA power for the Three-Way Trivalent Plan 3^(k-p)

In most articles, the test of the power of statistical tests used is omitted due to the difficulty in calculating type II error (β). For this purpose, the probability for the non-central F-distribution (with a non-zero non-central parameter δ) should be calculated for the critical value F_c corresponding to the probability of making a type I error (α) for the central F-distribution. While the central F-distribution characterizes how the F-test statistic is distributed when the null hypothesis is assumed to be true, the non-central F-distribution instead shows how the F-test statistic is distributed when the alternative hypothesis is assumed to be true (i.e., when the null hypothesis is assumed to be false). As such, it is useful in calculating the power of the usual F tests (ANOVA, regression, etc.) [11].

Knowledge of the value of the probability of making a type II error (β) makes it possible to calculate the test power according to Formula (S98). It was assumed that the minimum power cannot be lower than 80%, i.e., the probability of making a type II error cannot exceed 20%.

In each statistical test, a hypothesis is formulated first, which is subject to verification, and this tested hypothesis is referred to as the null hypothesis—H0. The null hypothesis is formulated in such a way that it can be

rejected based on the test results. In addition, an alternative hypothesis—H1—is formulated. It is assumed that the null hypothesis is true.

Two types of errors can be made when verifying hypotheses. The error of rejecting a true null hypothesis—type I error (error α). The error of adopting a false null hypothesis is a type II error (error β). The level of significance, marked with the symbol α, is chosen in advance as the probability of making the first kind of error. Rejection of the tested null hypothesis at the significance level of $\alpha = 0.05$ means that the risk of making the first kind of error with this decision was 5%. The power of the test is the probability of type II error (understood as the difference in certainty and the likelihood of making a type II error—Formula (S98)). In the case of the probability of type I error (α), a decision is made that there is a statistically significant difference between the analyzed means. Type II error (β) probability is based on the fact that a decision is made that there is no statistically significant difference between the analyzed means. The power of the test consists in the fact that it is reliably accepted that with the probability of type error I (α), there is a statistically significant difference between the analyzed means.

$$\text{Power} = 1 - \beta \tag{S98}$$

The recommended level of significance is 0.05, and the statistical testing power level is 0.8 [11]. The smaller the type II error, the more powerful the test. Increasing the level of significance (α), increasing the sample size, and improving the accuracy of measurements (e.g., by increasing the sensitivity of the measuring instrument) all improve the test power. However, increasing the level of significance also increases the probability of making a type I error.

In the case of analysis of the non-central F-distribution, a significant problem is the calculation of the non-central parameter δ (which, according to Equation (S99), is calculated from the root-mean-square standardized effect (RMSSE) for the "i" factor effect with "q" levels—Formula (S100) [11]). The RMSSE is the square root of the sum of squared standardized effects divided by the number of degrees of freedom for the effect. For individual main effects (Stata Software Manuals) and two-factor ANOVA interactions, this leads to Formulas (S101), where $\mathrm{SS}_{\mathrm{i.ef}}$ is the sum of the squares of the "i" main and interaction effects. The variance σ_e^2 is the probability of the measurement uncertainty for the test object in the hole research (in the case of this study, the object is a preform or bottle) in all measurement series. A summary of the measurement errors Δ_{k} of the measurement tools is shown in Table 3. For direct measurements, with uniform distribution of the density function of the probability density of the error distribution of the measuring instrument Δ_{k} and an arbitrarily accepted confidence level $p = 0.95$, the variance σ_e^2 was calculated by Formula (S102) (for direct measurements, i.e., degree of crystallinity in DSC, density in the gradient column, time τ_2 and τ_3, percentage intensity $I_1 + I_3$ and I_2). The problem is to determine the measurement variance (S102) for testing the microcavitation effect, the density of the oriented amorphous phase, and the relaxation of the amorphous phase because the measurement is done indirectly using either the apparatus for positron annihilation or a DSC apparatus and a gradient column. The variation of the measurement error σ_e^2 for the microcavitation effect can be calculated by Formula (S103), for the density of the oriented amorphous phase by Formula (S104), and for the relaxation of the amorphous phase by Formula (S105).

The non-central parameter can be understood as the surplus of variability between the means in the analyzed effect and the variability of the measurements for the object in a given measurement series (the higher the parameter δ, the greater the variability between the means of the effect in relation to the variability of the measurements of the object in measurement series, so the decision to assume the statistical significance of the effect is more reliable—the smaller the type II error). The steps for calculating the test power for each effect are summarized in Figure S4.

$$\delta_{ef.i} = N_i \cdot df_i \cdot RMSEE_{ef.i}{}^2 \quad where \quad i = A, B, C, AB, AC, BC \tag{S99}$$

$$RMSEE_{ef.i} = \frac{\sqrt{\dfrac{\sum_{j=1}^{q}\left(\bar{y}_{j\bullet\bullet\bullet} - \tilde{y}\right)^2}{df_i}}}{\sigma_e} \quad where \quad i = A, B, C, AB, AC, BC \tag{S100}$$

$$\delta_{\mathrm{i.ef}} = \frac{\mathrm{SS}_{\mathrm{i.ef}}}{\sigma_{\mathrm{e}}{}^2} \quad \text{where} \quad \mathrm{i} = \mathrm{A, B, C, AB, AC, BC} \tag{S101}$$

$$\sigma_e = k \cdot \sqrt{\left(\Delta_j/\sqrt{3}\right)^2 + \left(\sigma/\sqrt{N}\right)^2} \quad where \quad j = 1,2,\ldots,5 \text{ in Table 3} \tag{S102}$$

$$(\sigma_e)_{M.E.} = (\sigma_e)_{\rho_a} \tag{S103}$$

$$(\sigma_e)_{\rho_a} = \sqrt{\left(\frac{\rho_c^2\cdot\left(1-\widehat{C_{DSC}}\right)}{\left(\hat{\rho}\cdot\widehat{C_{DSC}}-\rho_c\right)^2}\cdot(\sigma_e)_\rho\right)^2 + \left(\frac{\hat{\rho}\cdot\rho_c\cdot(\hat{\rho}-\rho_c)}{\left(\hat{\rho}\cdot\widehat{C_{DSC}}-\rho_c\right)^2}\cdot(\sigma_e)_{DSC}\right)^2} \tag{S104}$$

$$(\sigma_e)_{Y_a} = \sqrt{\left(-\frac{\widehat{\rho_a^u}}{\rho_a^2}\cdot\sqrt{\left(\frac{\rho_c^2\cdot\left(1-\widehat{C_{DSC}}\right)}{\left(\hat{\rho}\cdot\widehat{C_{DSC}}-\rho_c\right)^2}\cdot(\sigma_e)_\rho\right)^2 + \left(\frac{\hat{\rho}\cdot\rho_c\cdot(\hat{\rho}-\rho_c)}{(\rho\cdot C_{DSC}-\rho_c)^2}\cdot(\sigma_e)_{DSC}\right)^2}\right)^2}\cdot 100\% \tag{S105}$$

where $\delta_{ef.i}$ is the non-central parameter of non-central F-distribution, [-]; N_i is the effect sample size, [-]; df_i is the effect degree of freedom, [-]; $RMSEE_{ef.i}$ is the root-mean-square standardized effect (RMSSE) for the "i" factor effect, [-]; Δh_f^o is the heat of melting a completely crystalline polymer in the same temperature range, $[J/(kg\cdot K)]$; $SS_{i.ef}$ is the sum of the squares of the "i" effect; σ_e^2 is the probability of the measurement uncertainty value of the measurement for the test object in the hole research (in the case of this study, the object is a preform or bottle) in all measurement series for direct measurements; k is the extension factor (for p = 0.95, k = 1.96); N is the number of repetitions of measurements for all measurement series (the sample size), [-]; Δ is the most likely absolute error value of measurement tool for the assumed confidence level p=0.95; σ is the standard deviation from the mean of measurements for A8 measurement series of bottle and p0.25 for preform; marking "^" means that the quantity refers to the mean of all measurements for all measurement series.

Figure S4 describes how to calculate the power of statistical tests for each linear effect in a three-factor design (formulas (S98)-(S105)) based on the authors' previous work [11,12], which used a two-factor design. The steps for calculating the test power for each main effect and two-way interaction in a three-factor design are summarized in Figure S4. Analogous to linear main effects, statistical power can be calculated for quadratic main effects.

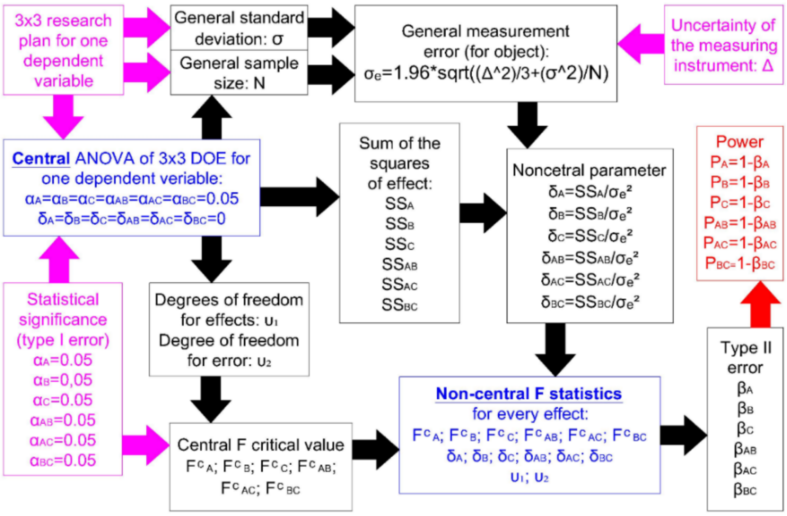

Figure S4. Procedure that allows defining the power of ANOVA tests for individual effects of the DOE analysis.

S.2. Research Results

Table 1[(a)] shows the CCF three-way, trivalent, incomplete design with 15 measurement series for the bottle (A1–A15), and Table 1[(b)] shows the one-factor three-valued design for the preform (p0.0, p0.25, p0.5). Studies on the density, intrinsic viscosity, thermal properties (DSC), and free volume (PALS) were carried out as part of these designs. The results of measurements of the physical and thermal properties of the preform and bottle material are summarized in Table S4; the lifetime and percentage intensity of subatomic antiparticles measured with the positron annihilation method of measuring the free volume of bottle and preform material and taking into account the dispersion σ_3 are summarized in Table S5, while the lifetime and percentage intensity of subatomic antiparticles measured in positron annihilation method of measuring the free volume of bottle and preform material and taking into account the dispersion σ_3 are summarized in Table S6. The results of measurements of the thickness profiles of

the bottle wall are summarized in Table S7, while the measurement results of the pressure resistance are summarized in Table S8.

In Tables S4, S5, S6, S7 and S8 for each dependent variable, the arithmetic mean (a), measurement uncertainty (b), and sample size (c) are given for each measurement series (shown in Table 1(a) and Table 1(b)). The statistical power of the ANOVA test was calculated for each experimental design. In Table S9 is presented the photos of two bottles from each measurement series, with extreme locations of the bottle cracking initiation points (after removing outliers) - the remaining bottles in the series cracked in the areas defined within the boundaries of the bottles shown.

Figure S5 additionally shows the standardized results of the bulk density, the microcavitation effect, the intrinsic viscosity, and the degree of crystallinity for each measurement series separately. While Figure S6 shows a summary of standardized results of PALS analysis at point III for each measuring series of the bottles (A1 ÷ A15) and preforms (p0.0, p0.25, p0.5) separately. Figure S7 shows a summary of standardized results of measurements of bottle material distribution (TH-I, TH-II, TH-III, i.e. thickness in point I, II, III), pressure resistance for each measurement series.

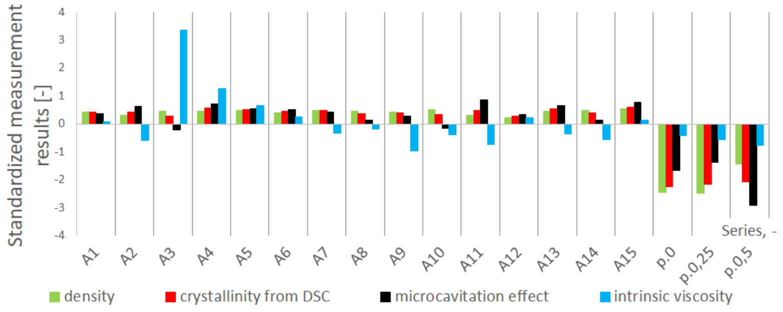

Figure S5. Summary of standardized results of measurements of material density, degree of crystallinity, intrinsic viscosity, and microcavitation in point III (see Figures 2a and 2b) for the series of bottles (A1 ÷ A15) and preforms (p0.0, p0.25, p0.5).

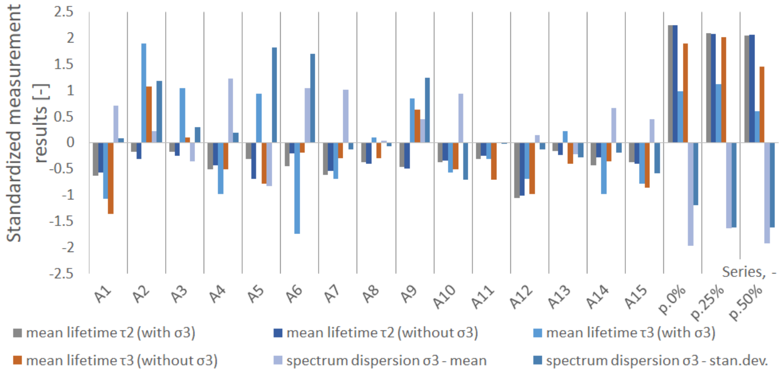

Figure S6. Summary of standardized results of PALS analysis in point III (see Figures 2a and 2b) for the series of bottles (A1 ÷ A15) and preforms (p0.0, p0.25, p0.5).

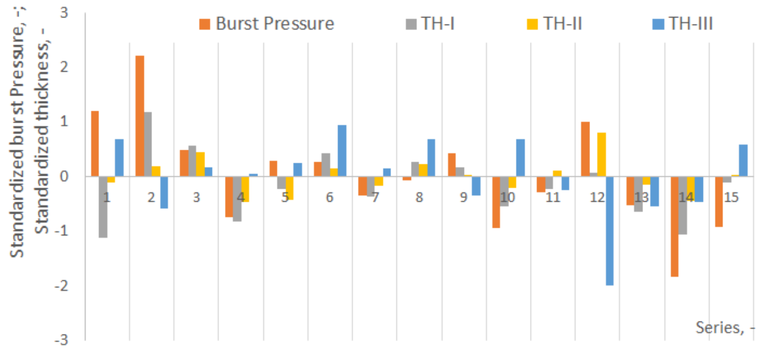

Figure S7. Summary of standardized results of pressure resistance and bottle material distribution (TH-I, TH-II, TH-III, i.e. thickness in point I, II, III) measurements (see Figure 2b) for the series of bottles (A1 ÷ A15).

Table S4. Presentation of research results on the physical and thermal properties of materials for bottles and preforms (measurement series are related to Table 1), where (a) is mean value; (b) is measurement uncertainty; (c) is sample size.

Physical and thermal properties		Responses for bottles (“A” series)															Responses for preforms		
		1	2	3	4	5	6	7	8	9	10	11	12	13	14	15	p0.0	p0.25	p0.5
Density [g/cm³]	(a)	1.3646	1.3635	1.3648	1.3649	1.3650	1.3644	1.3651	1.3647	1.3646	1.3654	1.3635	1.3629	1.3648	1.3651	1.3655	1.3409	1.3427	1.3492
	(b)	-	-	-	-	-	-	-	0.0002	-	-	-	-	-	-	-	-	0.0021	-
	(c)	1	1	1	1	1	1	1	3	1	1	1	1	1	1	1	1	3	1
Microcavitation effect [-]	(a)	0.0060	0.0070	0.0038	0.0073	0.0067	0.0065	0.0062	0.0051	0.0056	0.0040	0.0078	0.0058	0.0071	0.0051	0.0075	-	-	-
	(b)	-	-	-	-	-	-	-	0.0014	-	-	-	-	-	-	-	-	0.0019	-
	(c)	1	1	1	1	1	1	1	3	1	1	1	1	1	1	1	1	3	1
Relative density [-]	(a)	1.094	1.092	1.107	1.098	1.098	1.097	1.093	1.095	1.091	1.094	1.093	1.094	1.092	1.094	1.095	1.092	1.090	1.089
	(b)	-	-	-	-	-	-	-	0.003	-	-	-	-	-	-	-	-	0.006	-
	(c)	1	1	1	1	1	1	1	3	1	1	1	1	1	1	1	1	3	1
Intrinsic viscosity [dL/g]	(a)	0.905	0.886	0.999	0.939	0.922	0.910	0.894	0.898	0.875	0.892	0.882	0.909	0.893	0.887	0.907	0.891	0.887	0.881
	(b)	-	-	-	-	-	-	-	0.016	-	-	-	-	-	-	-	-	0.016	-
	(c)	1	1	1	1	1	1	1	3	1	1	1	1	1	1	1	1	3	1
Crystallinity DSC [%]	(a)	31.3	31.2	29.7	32.6	32.2	31.5	31.9	30.7	31.0	30.3	31.8	29.8	32.3	31.0	33.2	3.4	4.4	5.4
	(b)	-	-	-	-	-	-	-	1.2	-	-	-	-	-	-	-	-	0.8	-
	(c)	1	1	1	1	1	1	1	3	1	1	1	1	1	1	1	1	3	1
Glass transition temperature [°C]	(a)	73.51	73.35	74.94	75.41	74.46	74.03	74.87	74.76	73.12	75.64	73.55	75.07	73.78	76.59	73.64	74.65	74.98	75.42
	(b)	-	-	-	-	-	-	-	1.48	-	-	-	-	-	-	-	-	2.71	-
	(c)	1	1	1	1	1	1	1	3	1	1	1	1	1	1	1	1	3	1
Melting temperature [°C]	(a)	252.91	251.91	252.55	250.99	252.86	253.32	252.59	252.25	253.16	252.68	252.86	251.58	251.78	250.69	251.05	251.36	251.93	252.16
	(b)	-	-	-	-	-	-	-	2.44	-	-	-	-	-	-	-	-	1.59	-
	(c)	1	1	1	1	1	1	1	3	1	1	1	1	1	1	1	1	3	1
Cold crystallization enthalpy [J/g]	(a)	3.85	4.16	2.67	1.70	1.86	2.76	2.41	2.33	3.51	1.83	2.49	4.62	2.12	1.12	1.37	31.69	32.47	31.37
	(b)	-	-	-	-	-	-	-	0.76	-	-	-	-	-	-	-	-	2.25	-
	(c)	1	1	1	1	1	1	1	3	1	1	1	1	1	1	1	1	3	1
Total melting enthalpy [J/g]*	(a)	47.69	47.86	44.23	47.34	46.90	46.92	47.11	45.30	46.94	44.30	47.07	46.41	47.41	44.54	47.87	36.47	38.61	38.93
	(b)	-	-	-	-	-	-	-	1.38	-	-	-	-	-	-	-	-	1.24	-
	(c)	1	1	1	1	1	1	1	3	1	1	1	1	1	1	1	1	3	1
Melting enthalpy [J/g]**	(a)	43.84	43.70	41.56	45.64	45.04	44.16	44.70	42.97	43.43	42.47	44.58	41.79	45.29	43.42	46.50	4.78	6.14	7.56
	(b)	-	-	-	-	-	-	-	1.74	-	-	-	-	-	-	-	-	1.01	-
	(c)	1	1	1	1	1	1	1	3	1	1	1	1	1	1	1	1	3	1

*) Total melting enthalpy recorded during heating, including the contribution from cold crystallization.
**) Melting enthalpy recorded during heating after subtraction of the cold crystallization contribution. This value was used to determine the degree of crystallinity.

Table S5. Presentation of research results on the lifetime and percentage intensity of subatomic particles measured in positron annihilation method of measuring the free volume of bottle and preform material, taking into account the dispersion σ_3 (measurement series are related to Table 1), where (a) is mean value; (b) is measurement uncertainty; (c) is sample size.

Name	Feature		Responses for bottles ("A" series)															Responses for preforms		
			1	2	3	4	5	6	7	8	9	10	11	12	13	14	15	p0.0	p0.25	p0.5
Positron lifetime [ns]	τ_2 (mean)	(a)	0.3233	0.3275	0.3275	0.3244	0.3262	0.3250	0.3234	0.3256	0.3248	0.3256	0.3262	0.3193	0.3277	0.3251	0.3257	0.3501	0.3487	0.3482
		(b)	-	-	-	-	-	-	-	0.0049	-	-	-	-	-	-	-	-	0.0010	-
		(c)	1	1	1	1	1	1	1	3	1	1	1	1	1	1	1	1	3	1
	τ_2 (fitting uncertainty)	(a)	0.0014	0.0017	0.0017	0.0014	0.0016	0.0011	0.0012	0.0015	0.0019	0.0012	0.0017	0.0015	0.0012	0.0014	0.0010	0.0013	0.0009	0.0006
		(b)	-	-	-	-	-	-	-	0.0001	-	-	-	-	-	-	-	-	0.0003	-
		(c)	1	1	1	1	1	1	1	3	1	1	1	1	1	1	1	1	3	1
	τ_3 (mean)	(a)	1.4810	1.5430	1.5250	1.4830	1.5230	1.4670	1.4890	1.5053	1.5210	1.4915	1.4970	1.4890	1.5080	1.4830	1.4870	1.5240	1.5267	1.5160
		(b)	-	-	-	-	-	-	-	0.0237	-	-	-	-	-	-	-	-	0.0043	-
		(c)	1	1	1	1	1	1	1	3	1	1	1	1	1	1	1	1	3	1
	τ_3 (fitting uncertainty)	(a)	0.0330	0.0470	0.0380	0.0370	0.0540	0.0550	0.0320	0.0333	0.0480	0.0245	0.0360	0.0310	0.0290	0.0290	0.0240	0.0220	0.0157	0.0140
		(b)	-	-	-	-	-	-	-	0.0087	-	-	-	-	-	-	-	-	0.0073	-
		(c)	1	1	1	1	1	1	1	3	1	1	1	1	1	1	1	1	3	1
Intensity [%]	$I_1 + I_3$ (mean)	(a)	27.35	25.20	26.08	27.00	24.85	28.92	27.13	26.58	26.65	26.97	26.81	27.08	25.72	27.26	26.57	33.24	33.27	33.60
		(b)	-	-	-	-	-	-	-	1.07	-	-	-	-	-	-	-	-	0.29	-
		(c)	1	1	1	1	1	1	1	3	1	1	1	1	1	1	1	1	3	1
	$I_1 + I_3$ (fitting uncertainty)	(a)	0.40	0.62	0.59	0.47	0.51	0.35	0.40	0.51	0.63	0.72	0.57	0.46	0.44	0.51	0.27	0.31	0.22	0.19
		(b)	-	-	-	-	-	-	-	0.07	-	-	-	-	-	-	-	-	0.10	-
		(c)	1	1	1	1	1	1	1	3	1	1	1	1	1	1	1	1	3	1
	I_2 (mean)	(a)	72.65	74.80	73.92	73.00	75.15	71.08	72.87	73.42	73.35	73.04	73.19	72.92	74.28	72.74	73.43	66.76	66.73	66.40
		(b)	-	-	-	-	-	-	-	1.07	-	-	-	-	-	-	-	-	0.29	-
		(c)	1	1	1	1	1	1	1	3	1	1	1	1	1	1	1	1	3	1
	I_2 (fitting uncertainty)	(a)	0.40	0.62	0.59	0.47	0.51	0.35	0.40	0.51	0.63	0.72	0.57	0.46	0.44	0.51	0.27	0.31	0.22	0.19
		(b)	-	-	-	-	-	-	-	0.07	-	-	-	-	-	-	-	-	0.10	-
		(c)	1	1	1	1	1	1	1	3	1	1	1	1	1	1	1	1	3	1
Dispersion of ortho-positronium lifetime [ns]	σ_3 (mean)	(a)	0.514	0.493	0.467	0.537	0.446	0.529	0.528	0.485	0.503	0.524	0.483	0.489	0.473	0.512	0.503	0.396	0.410	0.398
		(b)	-	-	-	-	-	-	-	0.023	-	-	-	-	-	-	-	-	0.008	-
		(c)	1	1	1	1	1	1	1	3	1	1	1	1	1	1	1	1	3	1
	σ_3 (standard uncertainty)	(a)	0.047	0.067	0.051	0.049	0.078	0.076	0.043	0.044	0.068	0.032	0.045	0.043	0.040	0.042	0.034	0.030	0.022	0.022
		(b)	-	-	-	-	-	-	-	0.013	-	-	-	-	-	-	-	-	0.011	-
		(c)	1	1	1	1	1	1	1	3	1	1	1	1	1	1	1	1	3	1

Table S6. Presentation of research results on the lifetime and percentage intensity of subatomic particles measured in positron annihilation method of measuring the free volume of bottle and preform material after removal of the dispersion σ₃ (measurement series are related to Table 1), where (a) is mean value; (b) is measurement uncertainty; (c) is sample size.

Name	Feature		Responses for bottles ("A" series)															Responses for preforms		
			1	2	3	4	5	6	7	8	9	10	11	12	13	14	15	p0.0	p0.25	p0.5
Positron lifetime [ns]	τ_2 (mean)	(a)	0.3293	0.3318	0.3324	0.3307	0.3283	0.3328	0.3297	0.3310	0.3301	0.3316	0.3323	0.3251	0.3325	0.3321	0.3310	0.3563	0.3546	0.354
		(b)	-	-	-	-	-	-	-	0.0030	-	-	-	-	-	-	-	-	0.0002	-
		(c)	1	1	1	1	1	1	1	3	1	1	1	1	1	1	1	1	3	1
	τ_2 (fitting uncertainty)	(a)	0.0006	0.0005	0.0006	0.0005	0.0006	0.0007	0.0004	0.0005	0.0005	0.0003	0.0004	0.0006	0.0006	0.0005	0.0003	0.0008	0.0005	0.000
		(b)	-	-	-	-	-	-	-	0.0001	-	-	-	-	-	-	-	-	0.0002	-
		(c)	1	1	1	1	1	1	1	3	1	1	1	1	1	1	1	1	3	1
	τ_3 (mean)	(a)	1.5318	1.5683	1.5537	1.5447	1.5405	1.5495	1.5477	1.5479	1.5617	1.5447	1.5417	1.5376	1.5462	1.5469	1.5394	1.5808	1.5825	1.574
		(b)	-	-	-	-	-	-	-	0.0049	-	-	-	-	-	-	-	-	0.0002	-
		(c)	1	1	1	1	1	1	1	3	1	1	1	1	1	1	1	1	3	1
	τ_3 (fitting uncertainty)	(a)	0.0061	0.0062	0.0063	0.0060	0.0071	0.0077	0.0047	0.0060	0.0087	0.0025	0.0040	0.0060	0.0058	0.0054	0.0034	0.0045	0.0028	0.005
		(b)	-	-	-	-	-	-	-	0.0009	-	-	-	-	-	-	-	-	0.0005	-
		(c)	1	1	1	1	1	1	1	3	1	1	1	1	1	1	1	1	3	1
Intensity [%]	$I_1 + I_3$ (mean)	(a)	25.56	24.38	25.03	24.88	24.78	25.77	25.06	25.05	25.24	24.61	25.08	25.24	24.40	24.84	24.83	30.49	30.52	30.77
		(b)	-	-	-	-	-	-	-	0.46	-	-	-	-	-	-	-	-	0.14	-
		(c)	1	1	1	1	1	1	1	3	1	1	1	1	1	1	1	1	3	1
	$I_1 + I_3$ (fitting uncertainty)	(a)	0.10	0.09	0.10	0.09	0.10	0.12	0.07	0.09	0.11	0.03	0.05	0.10	0.09	0.09	0.05	0.08	0.07	0.08
		(b)	-	-	-	-	-	-	-	0.01	-	-	-	-	-	-	-	-	0.03	-
		(c)	1	1	1	1	1	1	1	3	1	1	1	1	1	1	1	1	3	1
	I_2 (mean)	(a)	74.45	75.62	74.97	75.12	75.22	74.23	74.94	74.95	74.76	75.39	74.93	74.76	75.60	75.16	75.17	69.51	69.48	69.24
		(b)	-	-	-	-	-	-	-	0.46	-	-	-	-	-	-	-	-	0.14	-
		(c)	1	1	1	1	1	1	1	3	1	1	1	1	1	1	1	1	3	1
	I_2 (fitting uncertainty)	(a)	0.10	0.09	0.10	0.09	0.10	0.12	0.07	0.09	0.11	0.03	0.05	0.10	0.09	0.09	0.05	0.08	0.07	0.08
		(b)	-	-	-	-	-	-	-	0.01	-	-	-	-	-	-	-	-	0.03	-
		(c)	1	1	1	1	1	1	1	3	1	1	1	1	1	1	1	1	3	1
Dispersion of ortho-positronium lifetime [ns]	σ_3 (mean)	(a)	0	0	0	0	0	0	0	0	0	0	0	0	0	0	0	0	0	0
		(b)	0	0	0	0	0	0	0	0	0	0	0	0	0	0	0	0	0	0
		(c)	0	0	0	0	0	0	0	0	0	0	0	0	0	0	0	0	0	0
	σ_3 (standard uncertainty)	(a)	0	0	0	0	0	0	0	0	0	0	0	0	0	0	0	0	0	0
		(b)	0	0	0	0	0	0	0	0	0	0	0	0	0	0	0	0	0	0
		(c)	0	0	0	0	0	0	0	0	0	0	0	0	0	0	0	0	0	0

Table S7. Summary of the results of the study of the thickness profiles (measurement series are related to Table **1**), where (a) is the mean value; (b) is the measurement uncertainty; (c) is the sample size (after removing outliers*).

Name	Feature		Results concerning bottles from the series in Table 1														
			A1	A2	A3	A4	A5	A6	A7	A8	A9	A10	A11	A12	A13	A14	A15
Thickness profiles (Figure 2b)	I-1 [µm]	(a)	295	317	296	263	286	286	271	286	288	276	283	321	274	252	280
		(b)	9.1	22.3	8.6	2.4	9.9	11.7	6.9	3.5	9.5	3.5	13.4	19.9	5.1	9.6	2.3
		(c)	5	5	5	4	5	4	5	5	5	4	5	5	3	5	3
	I-2 [µm]	(a)	305	303	295	265	287	311	278	302	293	270	278	281	275	263	284
		(b)	9.1	1.7	9.5	4.1	16.4	17.7	8.5	6.4	6.0	8.0	27.6	12.9	3.5	4.5	1.5
		(c)	3	3	4	4	5	4	5	4	4	5	5	3	4	5	4
	I-3 [µm]	(a)	293	321	291	268	262	293	277	286	289	270	270	298	261	258	279
		(b)	2.9	39.3	15.9	7.1	40.4	22.2	7.0	19.0	10.1	9.1	6.2	51.6	12.0	2.1	9.2
		(c)	3	5	5	5	5	4	5	5	4	5	4	5	5	4	5
	II-1 [µm]	(a)	269	276	279	266	271	269	260	267	265	268	271	279	270	258	265
		(b)	3.1	1.5	6.1	2.2	2.1	5.3	1.5	2.6	2.2	1.5	1.3	20.5	1.5	4.7	6.3
		(c)	4	3	4	4	5	5	4	4	4	3	4	5	3	4	4
	II-2 [µm]	(a)	258	269	279	267	275	272	263	279	267	258	272	272	272	263	276
		(b)	19.4	4.6	3.2	0.5	5.9	11.6	2.8	3.4	2.2	0.6	8.2	8.7	8.5	2.3	3.7
		(c)	5	4	5	4	5	5	4	4	4	3	5	4	5	5	4
	II-3 [µm]	(a)	272	273	278	263	254	268	269	270	268	270	267	287	263	263	271
		(b)	7.6	2.8	7.6	10.2	21.8	7.6	2.6	6.2	2.9	2.9	3.1	1.7	2.1	0.6	4.4
		(c)	5	5	4	5	3	5	4	5	3	4	4	3	3	3	4
	III-1 [µm]	(a)	285	269	278	268	288	292	265	282	263	288	264	234	264	262	274
		(b)	5.4	6.7	12.6	1.5	6.5	8.9	6.8	7.6	2.9	6.1	4.6	4.0	11.3	3.7	15.0
		(c)	4	4	4	4	5	5	4	5	5	5	5	5	4	4	5
	III-2 [µm]	(a)	289	272	284	273	280	287	273	289	264	287	265	236	268	265	291
		(b)	13.9	8.3	13.1	3.5	6.6	5.7	5.5	2.6	5.1	2.9	3.0	8.2	3.2	1.9	1.9
		(c)	4	4	4	5	5	5	4	4	4	4	4	5	4	5	4
	III-3 [µm]	(a)	294	272	274	276	258	286	275	283	264	277	270	234	268	266	283
		(b)	5.4	9.6	4.9	2.5	20.6	9.8	5.5	8.2	10.3	14.9	2.9	8.3	4.8	2.4	5.6
		(c)	5	4	4	4	5	5	5	5	5	5	4	5	4	5	5
	I [µm] - entire cross-section I	(a)	298	311	294	265	288	293	275	290	289	271	278	300	271	259	281
		(b)	11.4	28.7	11.2	6.5	12.1	24.1	7.7	13.2	8.8	7.8	17.9	42.2	12.7	6.3	8.0
		(c)	13	15	14	15	13	15	15	15	14	15	15	15	14	13	15

	II [µm] - entire cross-section II	(a)	269	272	279	267	273	270	264	272	267	263	270	281	270	262	269
		(b)	6.8	4.1	5.3	2.5	4.9	8.2	5.7	6.2	3.1	8.5	5.9	17.0	6.9	2.3	6.9
		(c)	13	13	13	12	11	15	15	15	12	15	15	15	13	11	14
	III [µm] -entire cross-section III	(a)	288	269	280	272	284	288	274	284	263	283	266	235	267	265	283
		(b)	10.5	9.3	11.4	5.0	7.3	8.2	9.5	6.9	6.4	10.1	5.0	6.6	5.4	2.4	8.3
		(c)	14	13	13	15	12	15	15	15	14	15	15	15	12	13	14
* the methodology of statistical pre-processing of measurement data has been described in another work [11]																	

Table S8. Summary of the results of the study of the pressure resistance (measurement series are related to Table 1), where (a) is the mean value; (b) is the measurement uncertainty; (c) is the sample size (after removing outliers*).

Name	Feature		Results concerning bottles from the series in Table 1														
			A1	A2	A3	A4	A5	A6	A7	A8	A9	A10	A11	A12	A13	A14	A15
Pressure resistance	Burst pressure	(a)	14.6	15.5	14.0	12.9	13.8	13.8	13.2	13.5	13.9	12.7	13.3	14.4	13.0	11.9	12.7
		(b)	0.47	0.34	0.51	0.20	0.38	0.08	0.17	0.05	0.16	0.18	0.23	0.26	0.25	0.15	0.09
		(c)	10	9	10	9	10	8	10	8	8	10	9	6	10	8	9
* the methodology of statistical pre-processing of measurement data has been described in another work [11]																	

materials

Table S9. Photos of two bottles from each measurement series, with extreme locations of the bottle cracking initiation points (after removing outliers) - the remaining bottles in the series cracked in the areas defined within the boundaries of the bottles shown.

Series - Bottle	Just before bursting	Just after bursting	Crack shape	Remarks
A1-2	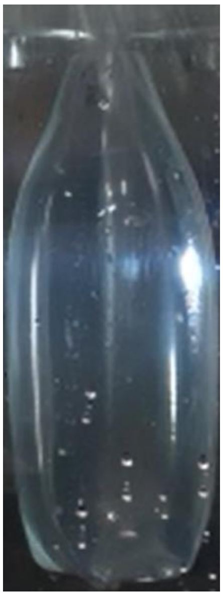	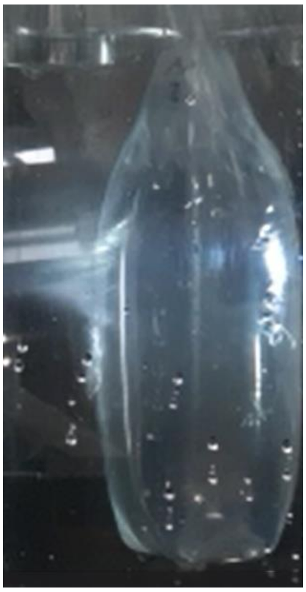	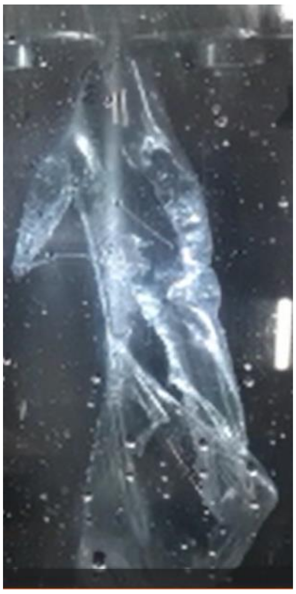	cracked in the area of the label
A1-11	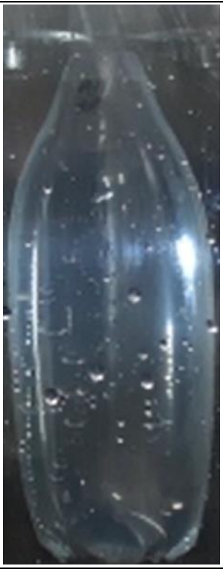	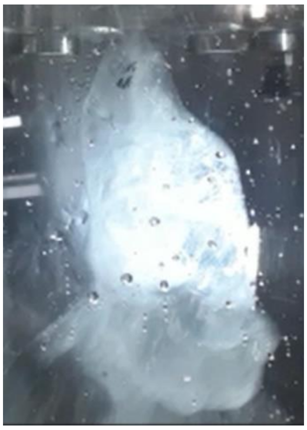	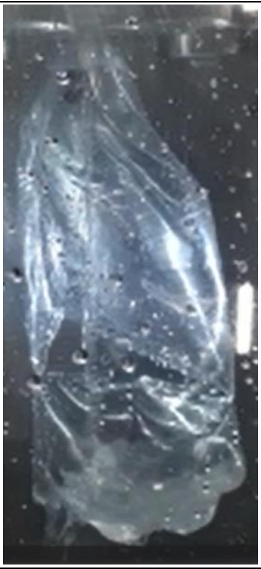	cracked in the area of the bottom

A2-2	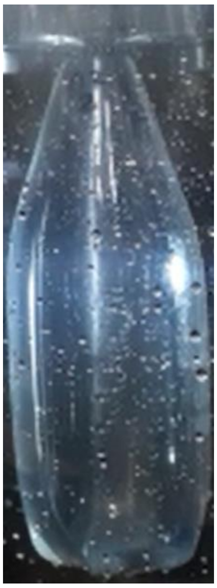	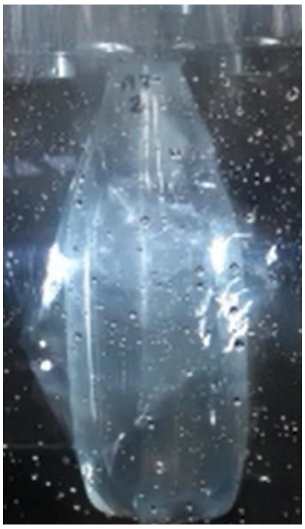	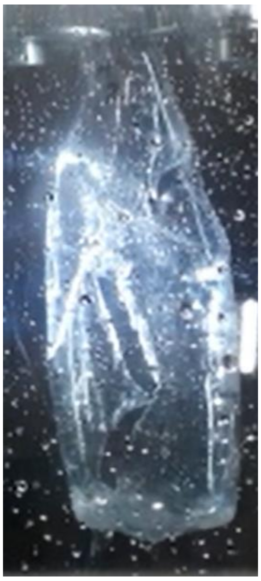	cracked in the area of the label
A2-14	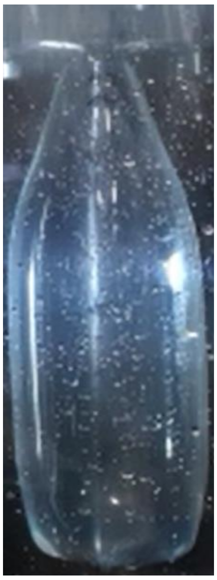	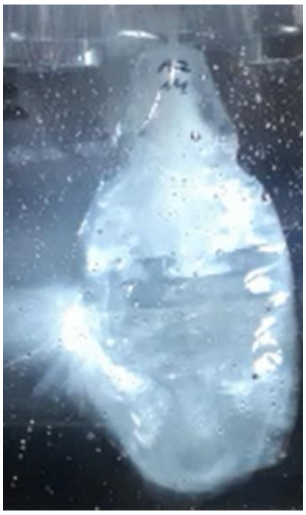	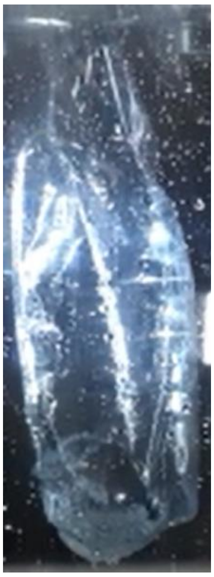	cracked in the area of the label
A3-9	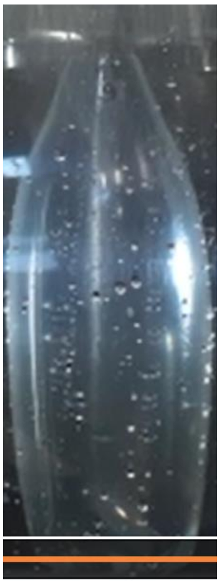	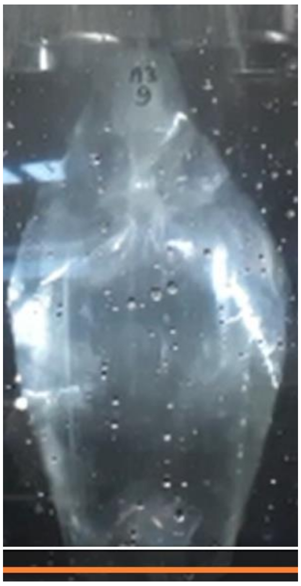	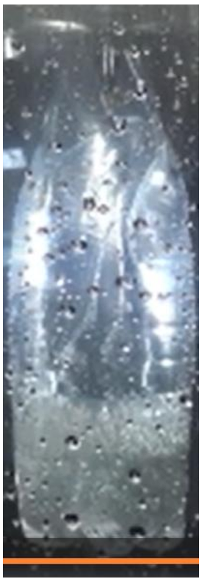	cracked in the area of the label

A3-15	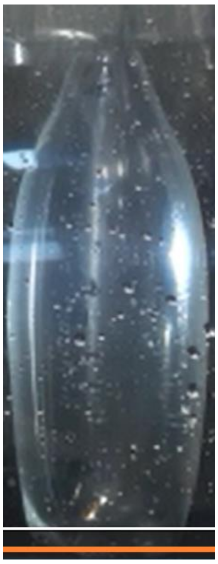	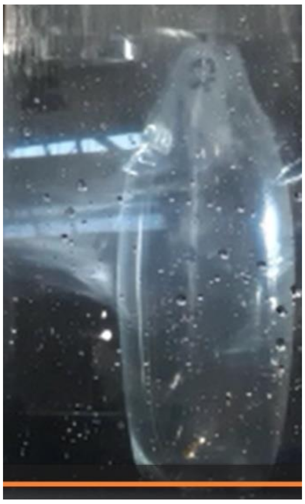	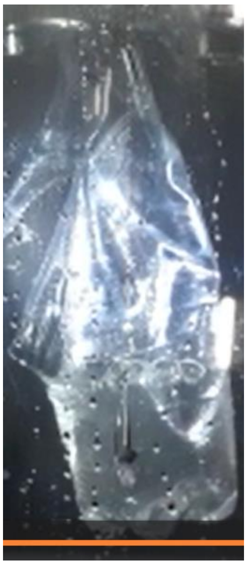	cracked in the area of the label near shoulders
A4-2	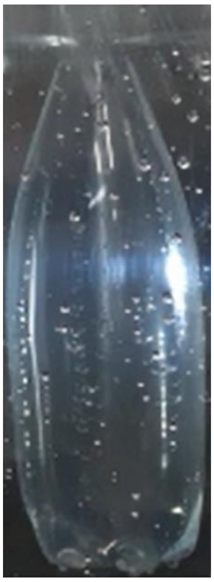	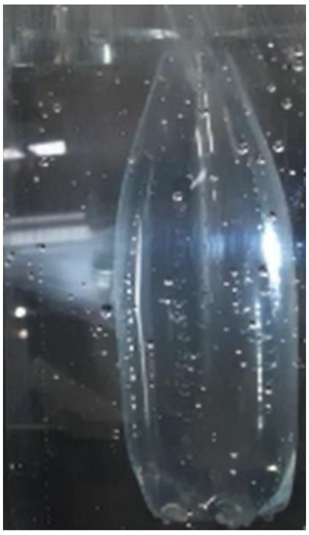	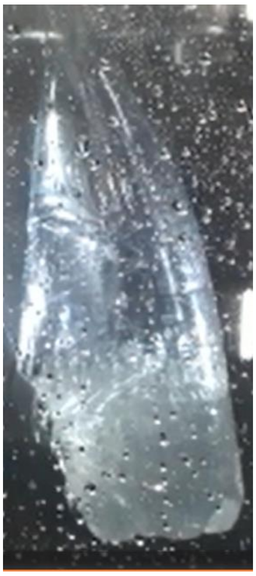	cracked in the area of the label
A4-13	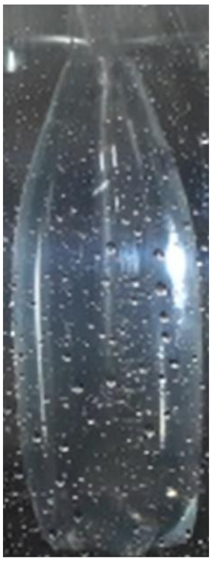	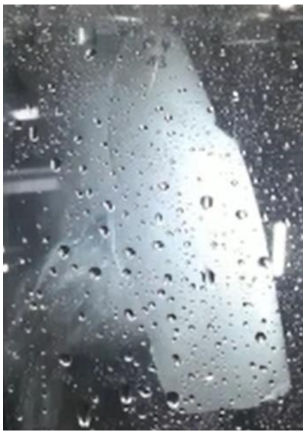	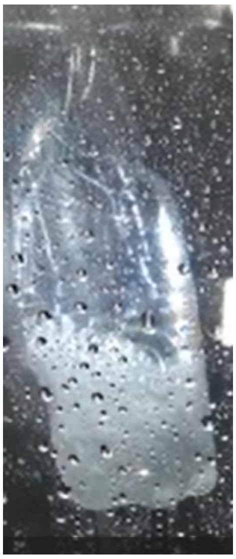	cracked in the area of the label

A5-4	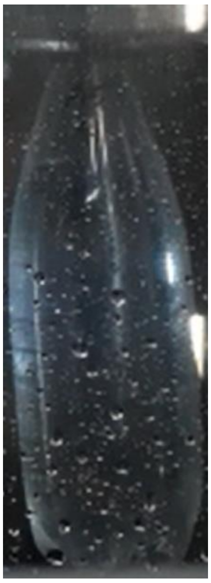	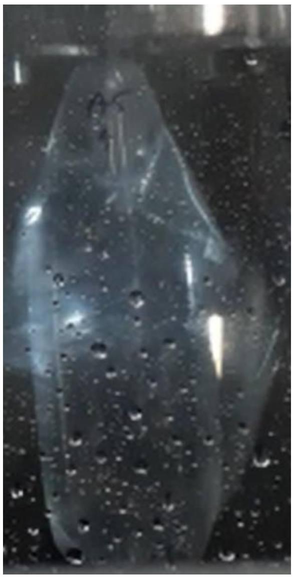	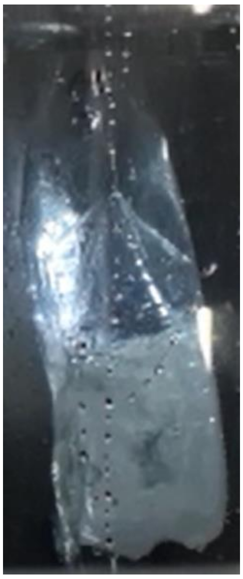	cracked in the area of the label
A5-14	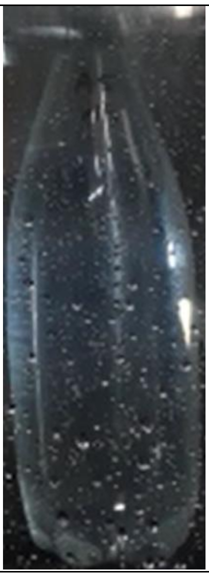	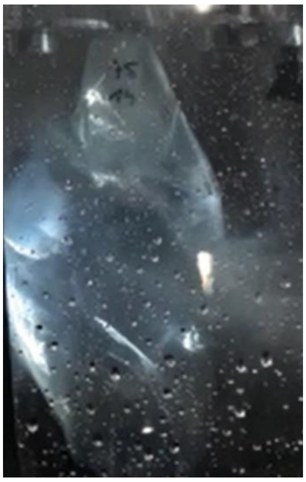	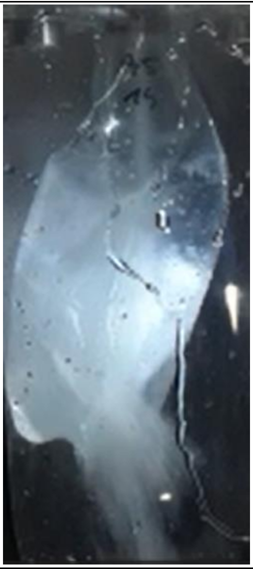	cracked in the area of the label
A6-3	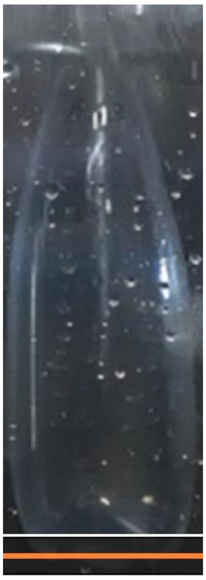	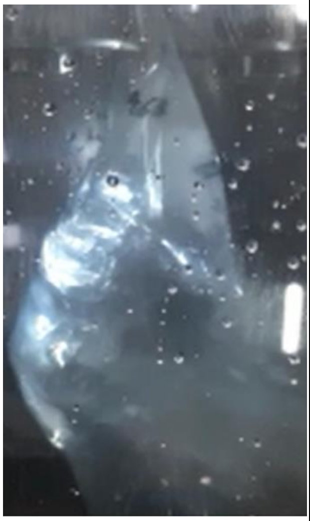	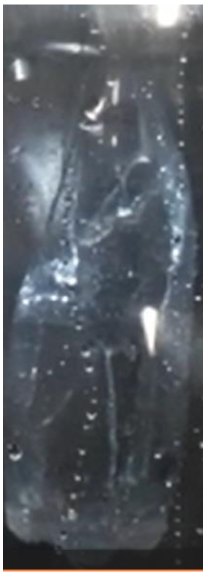	cracked in the area of the bottom

A6-7	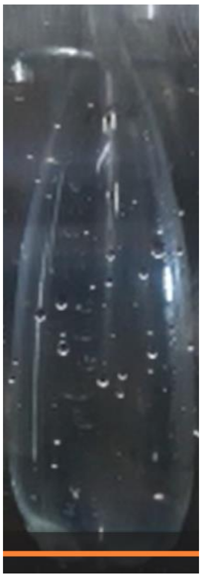	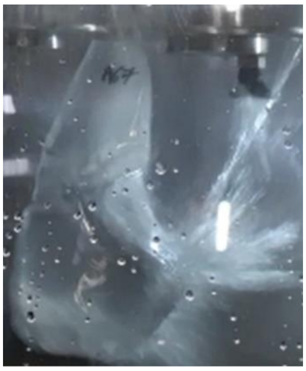	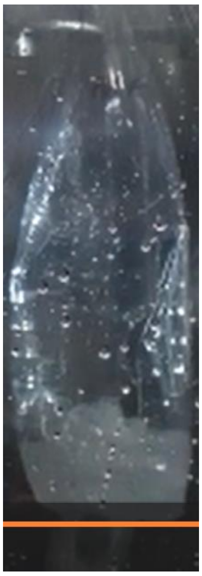	cracked in the area of the bottom
A7-6	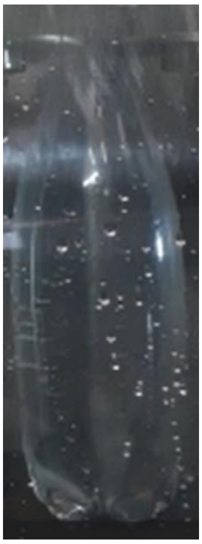	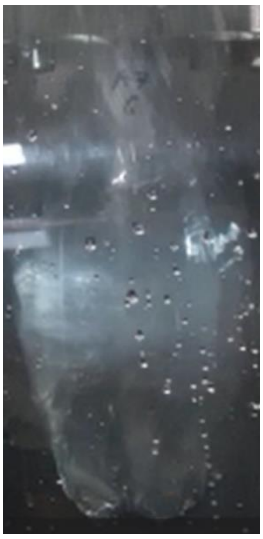	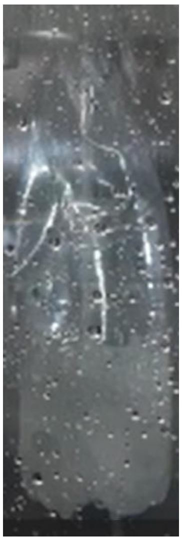	cracked in the area of the label
A7-14	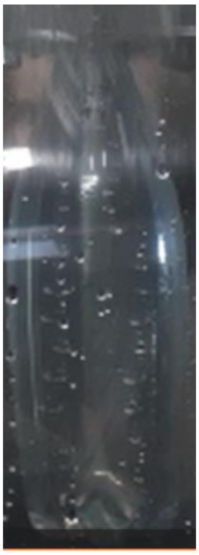	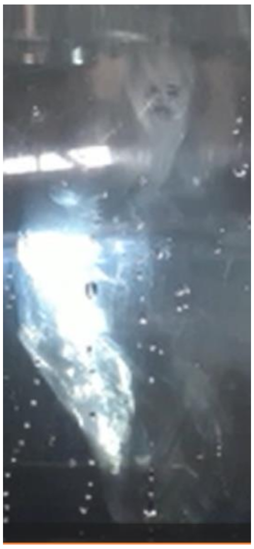	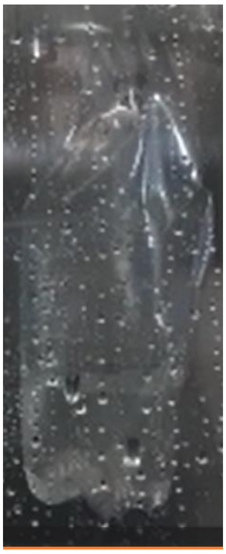	cracked in the area of the label

A8-8	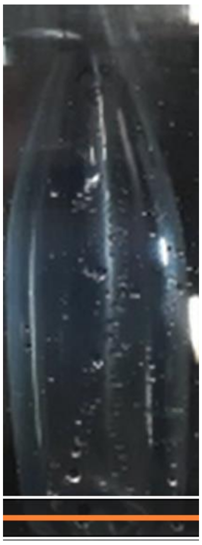	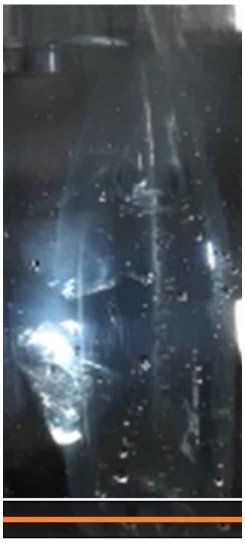	no picture	cracked in the area of the label
A8-9	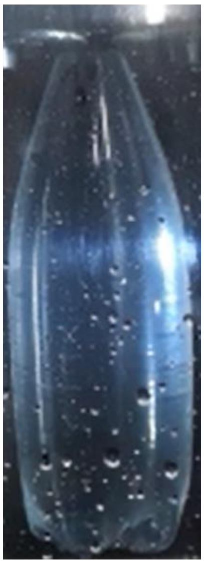	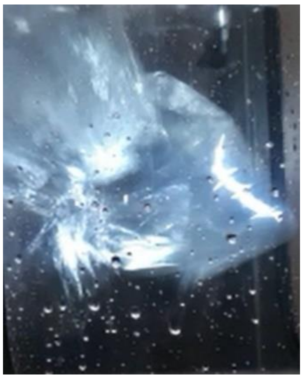	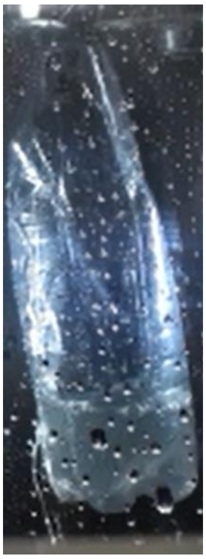	cracked in the area of the label
A9-13	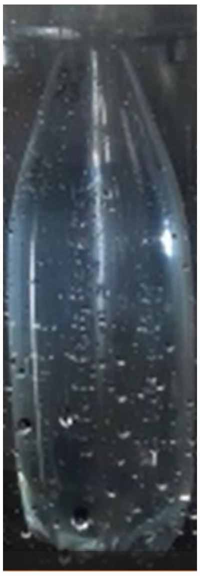	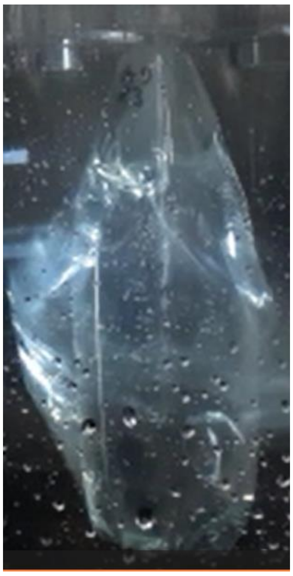	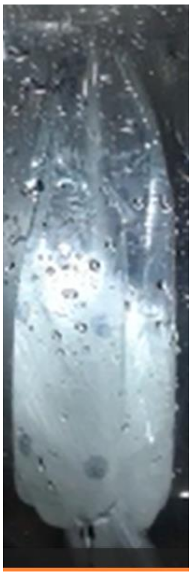	cracked in the area of the label

A9-15	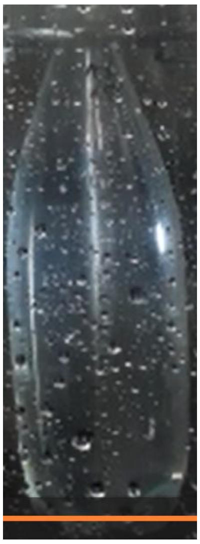	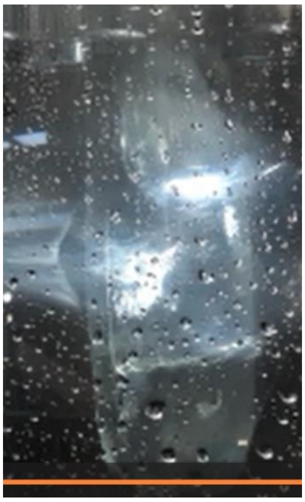	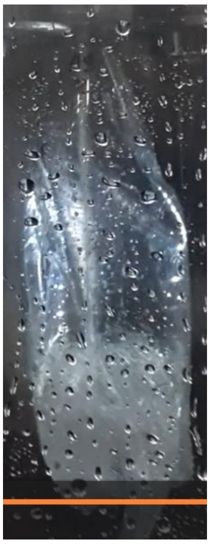	cracked in the area of the label
A10-7	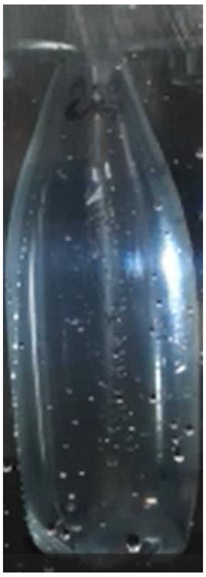	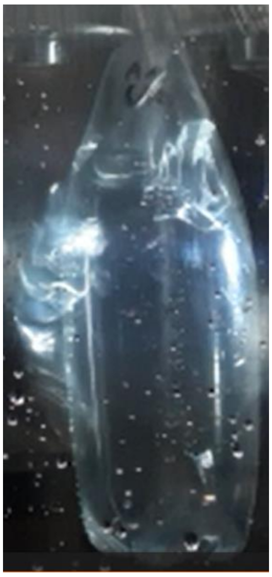	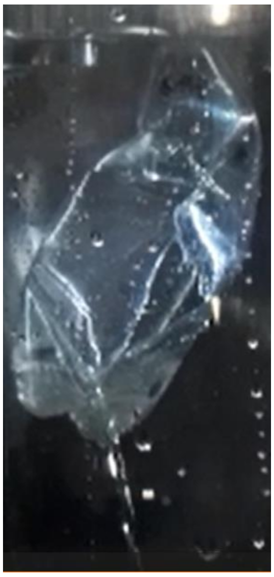	cracked in the area of the label near shoulders
A10-13	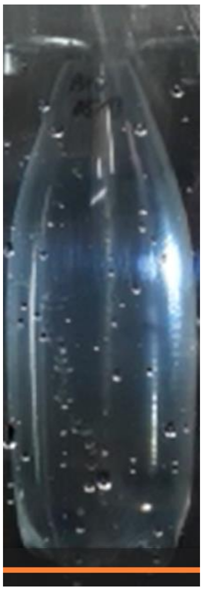	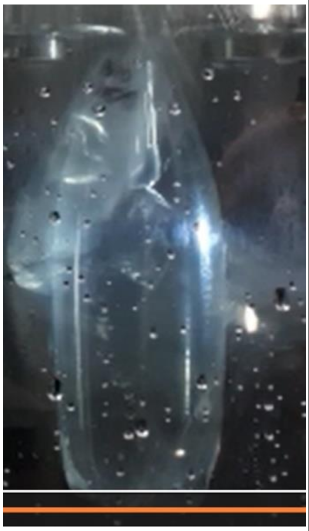	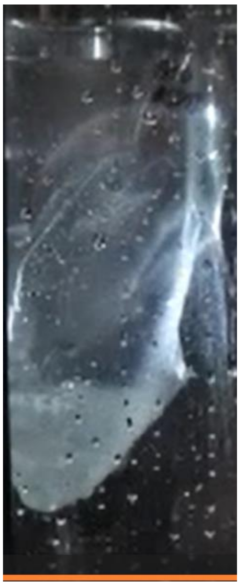	cracked in the area of the label near shoulders

A11-9	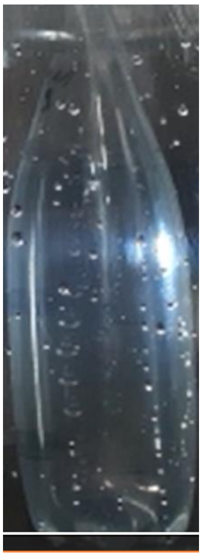	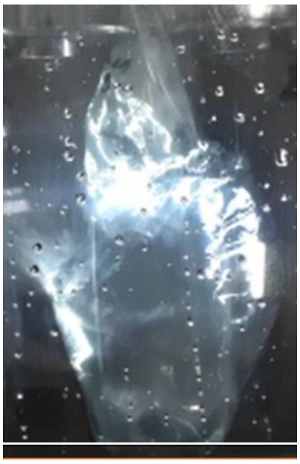	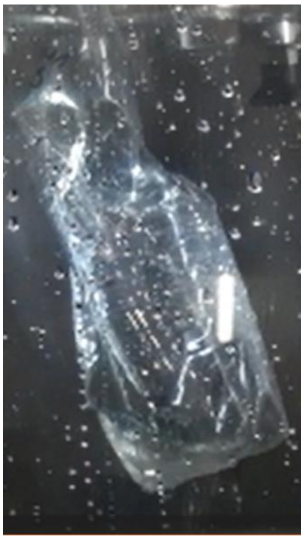	cracked in the area of the label
A11-10	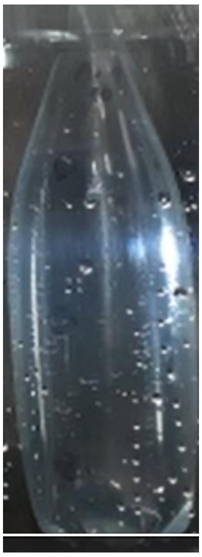	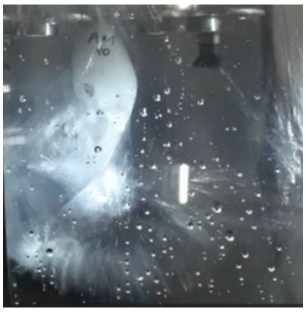	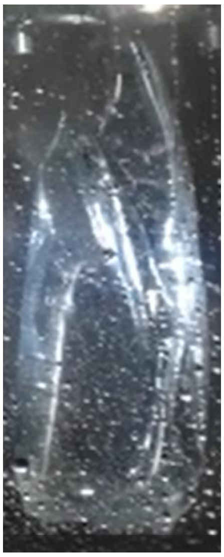	cracked in the area of the label
A12-1	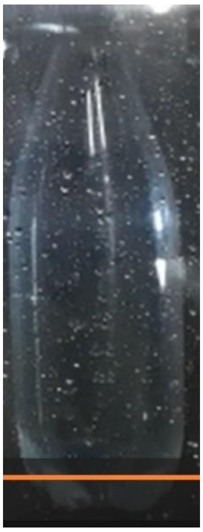	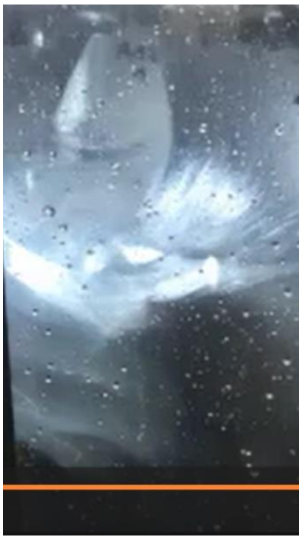	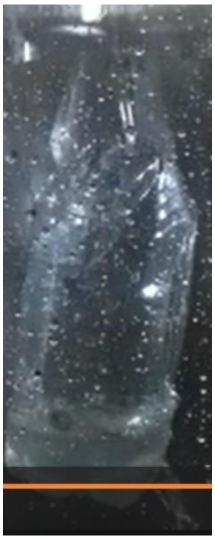	cracked in the area of the label

A12-12	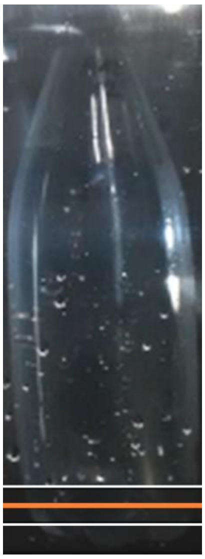	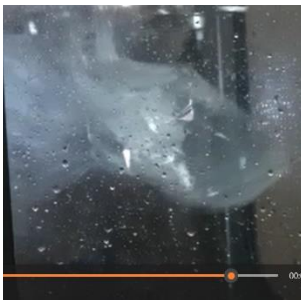	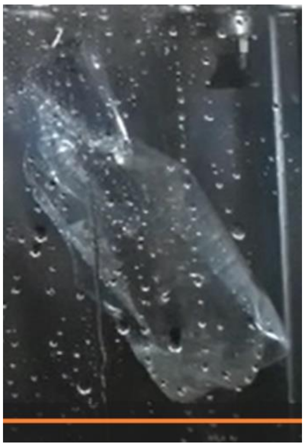	cracked in the area of the label near shoulders
A13-3	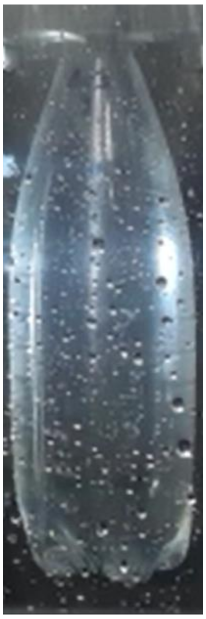	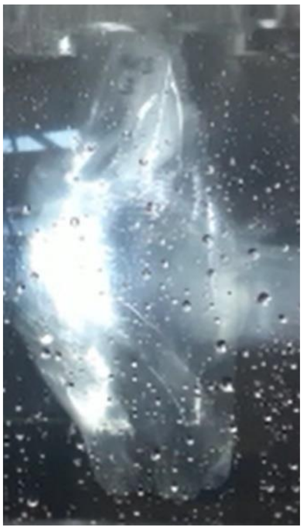	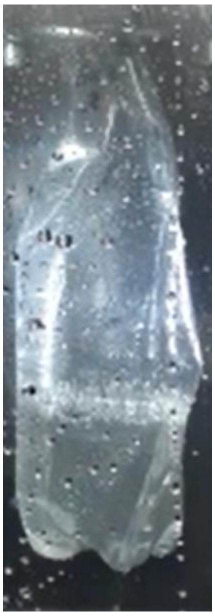	cracked in the area of the label
A13-15	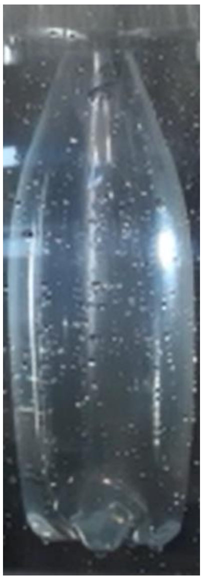	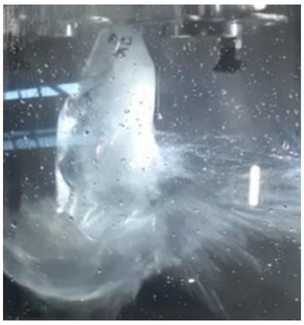	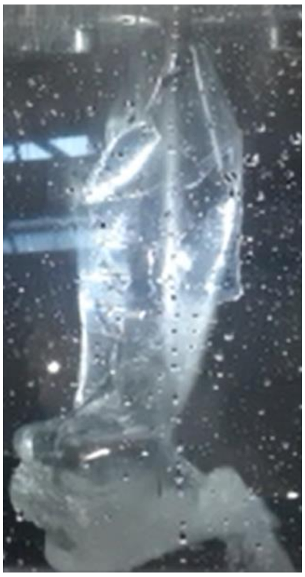	cracked in the area of the label near bottom

A14-3	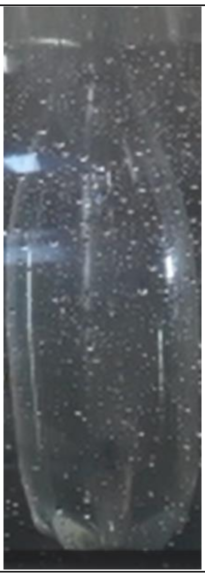	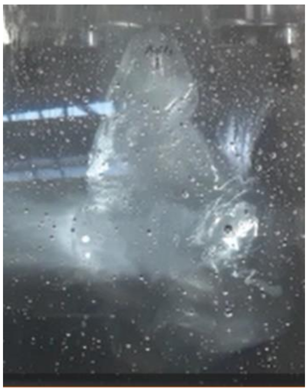	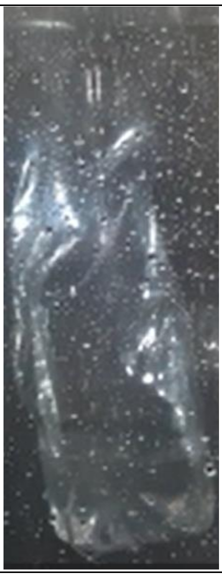	cracked in the area of the label
A14-10	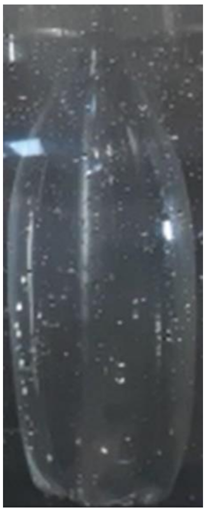	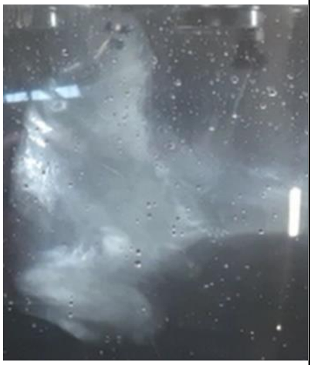	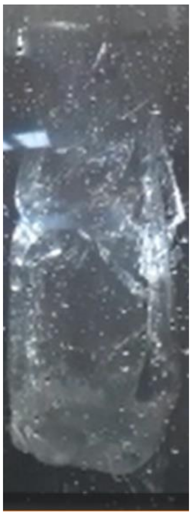	cracked in the area of the label
A15-1	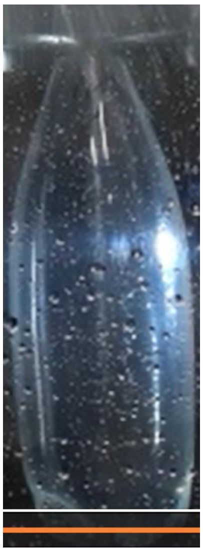	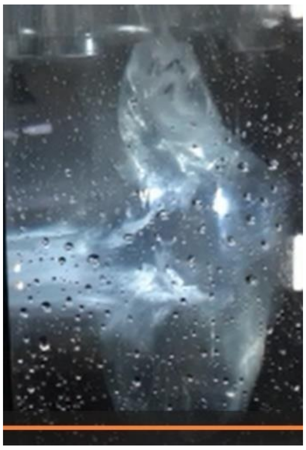	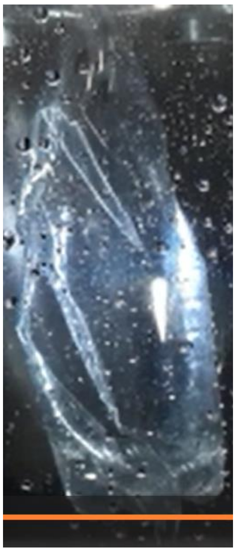	cracked in the area of the label

A15-15	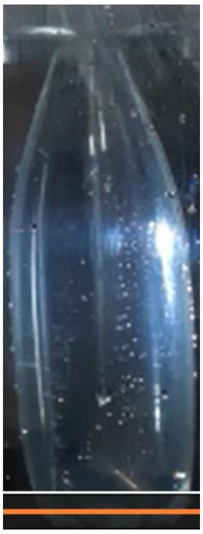	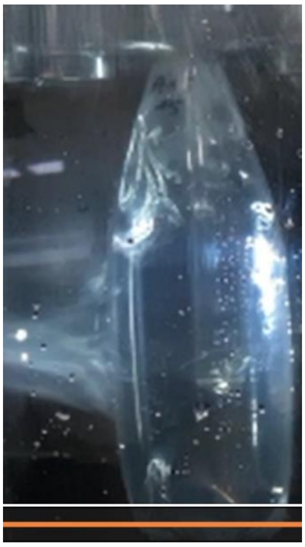	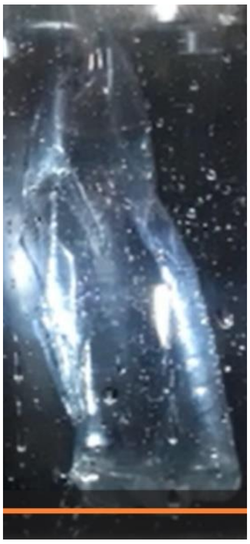	cracked in the area of the label
